# Controlled Selectivity through Reversible Inhibition of the Catalyst: Stereodivergent Semihydrogenation of Alkynes

**DOI:** 10.1021/jacs.2c04233

**Published:** 2022-07-15

**Authors:** Jie Luo, Yaoyu Liang, Michael Montag, Yael Diskin-Posner, Liat Avram, David Milstein

**Affiliations:** † Department of Molecular Chemistry and Materials Science, Weizmann Institute of Science, Rehovot 76100, Israel; ‡ Department of Chemical Research Support, Weizmann Institute of Science, Rehovot 76100, Israel

## Abstract

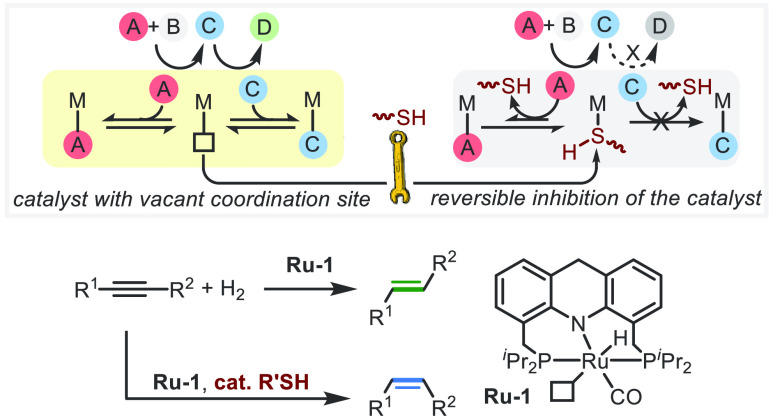

Catalytic semihydrogenation of internal alkynes using H_2_ is an attractive atom-economical route to various alkenes, and its stereocontrol has received widespread attention, both in homogeneous and heterogeneous catalyses. Herein, a novel strategy is introduced, whereby a poisoning catalytic thiol is employed as a reversible inhibitor of a ruthenium catalyst, resulting in a controllable H_2_-based semihydrogenation of internal alkynes. Both (*E*)- and (*Z*)-alkenes were obtained efficiently and highly selectively, under very mild conditions, using a single homogeneous acridine-based ruthenium pincer catalyst. Mechanistic studies indicate that the (*Z*)-alkene is the reaction intermediate leading to the (*E*)-alkene and that the addition of a catalytic amount of bidentate thiol impedes the *Z*/*E* isomerization step by forming stable ruthenium thiol(ate) complexes, while still allowing the main hydrogenation reaction to proceed. Thus, the absence or presence of catalytic thiol controls the stereoselectivity of this alkyne semihydrogenation, affording either the (*E*)-isomer as the final product or halting the reaction at the (*Z*)-intermediate. The developed system, which is also applied to the controllable isomerization of a terminal alkene, demonstrates how metal catalysis with switchable selectivity can be achieved by reversible inhibition of the catalyst with a simple auxiliary additive.

## Introduction

**Scheme 1:**
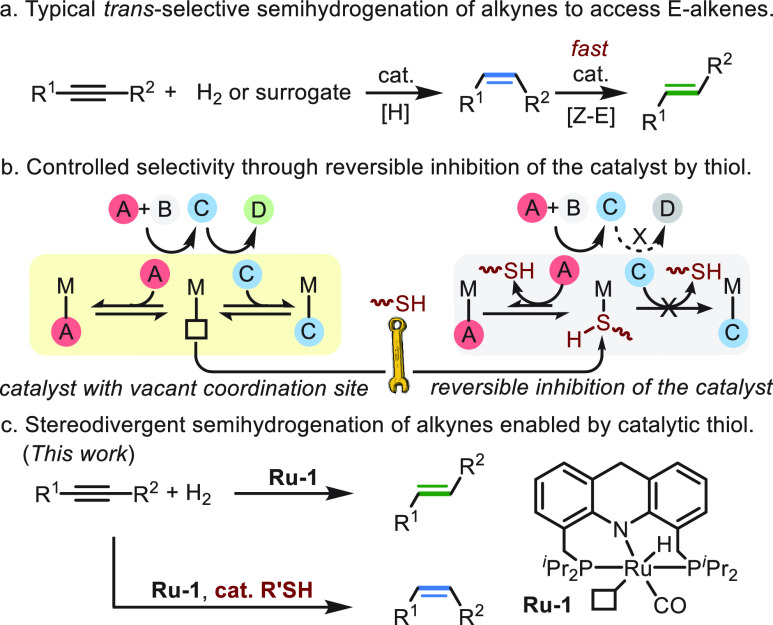
Controllable *E*/*Z* Semihydrogenation of Alkynes Enabled by Reversible Inhibition of the Catalyst

Many catalytic (de)hydrogenative reactions involve in situ generated intermediates, which are usually reactive under the catalytic conditions and are therefore seldom isolated as products.^1−7^ For example, the *trans*-selective catalytic semihydrogenation of alkynes typically begins with *cis*-hydrogenation, but the generated (*Z*)-alkene is only a kinetic intermediate, which is then rapidly isomerized into the thermodynamically more stable (*E*)-alkene product (Scheme 1a).^8−14^ If a strategy can be devised to slow down a specific reaction step, such as the *Z*-to-*E* isomerization in the *trans*-semihydrogenation of alkynes, the reactive intermediates can be stabilized and may even be isolable as end products from the same system, which would be of great interest and is highly advantageous. Nevertheless, the state-of-the-art methodologies to selectively access both (*E*)- and (*Z*)-alkenes through semihydrogenation of alkynes rely on the utilization of different catalysts.^15−22^ Some strategies involve the use of additives in order to switch the stereoselectivities of these transformations,^23−27^ but their mechanisms are unclear and they are usually limited to transfer semihydrogenation with H_2_ surrogates and also require stoichiometric amounts of additives, which inevitably generates waste and is neither atom-economical nor sustainable.

Thiol(ate)s are known to have very strong intrinsic affinity for transition metals and are therefore widely used as ligands,^28−31^ biological inhibitors,^32,33^ metal ion probes,^34−36^ and the end groups of self-assembled monolayers.^37,38^ However, in transition metal catalysis, this strong affinity of thiol(ate)s is usually problematic, since it can easily poison the catalysts by blocking their metal centers.^39−45^ For example, this poisoning effect of thiols deactivates the catalytically active Au sites in supported Au nanoparticles used for benzyl alcohol oxidation.^45^ Nevertheless, coordinated thiols can dissociate from metal centers and be displaced by other molecules.^46^ Therefore, we wondered whether adding a catalytic amount of thiol to a reaction mixture containing a given transition-metal-based catalyst could afford a protected form of this catalyst in situ, with the thiol acting as a reversible inhibitor of the catalyst.^47^ Ideally, this thiol protecting group would selectively open the coordination site only for targeted molecules, such as alkynes, and efficiently impede other unwanted reactions. This is in line with some previously reported homogeneous and heterogeneous catalytic systems, wherein different additives were used in order to tune the activity of the catalysts and improve their selectivity.^48−59^ Thus, in our proposed system, the simple addition of a catalytic amount of thiol could serve as a switch to control the selectivity of reactions by reversibly protecting the metal center (Scheme 1b).

Herein, we demonstrate the use of thiol poisoning as an effective means of achieving controllable semihydrogenation of alkynes with H_2_, in a process that is homogeneously catalyzed by an acridine-based PNP-type ruthenium pincer complex under very mild conditions (Scheme 1c). In the absence of thiol, this system enables the highly efficient *trans*-selective semihydrogenation of internal alkynes into *E*-alkenes, while in the presence of a catalytic amount of thiol it selectively affords *Z*-alkenes.

## Results and Discussion

### Establishment and Optimization of the Catalytic Reaction Conditions

**Figure 1:**
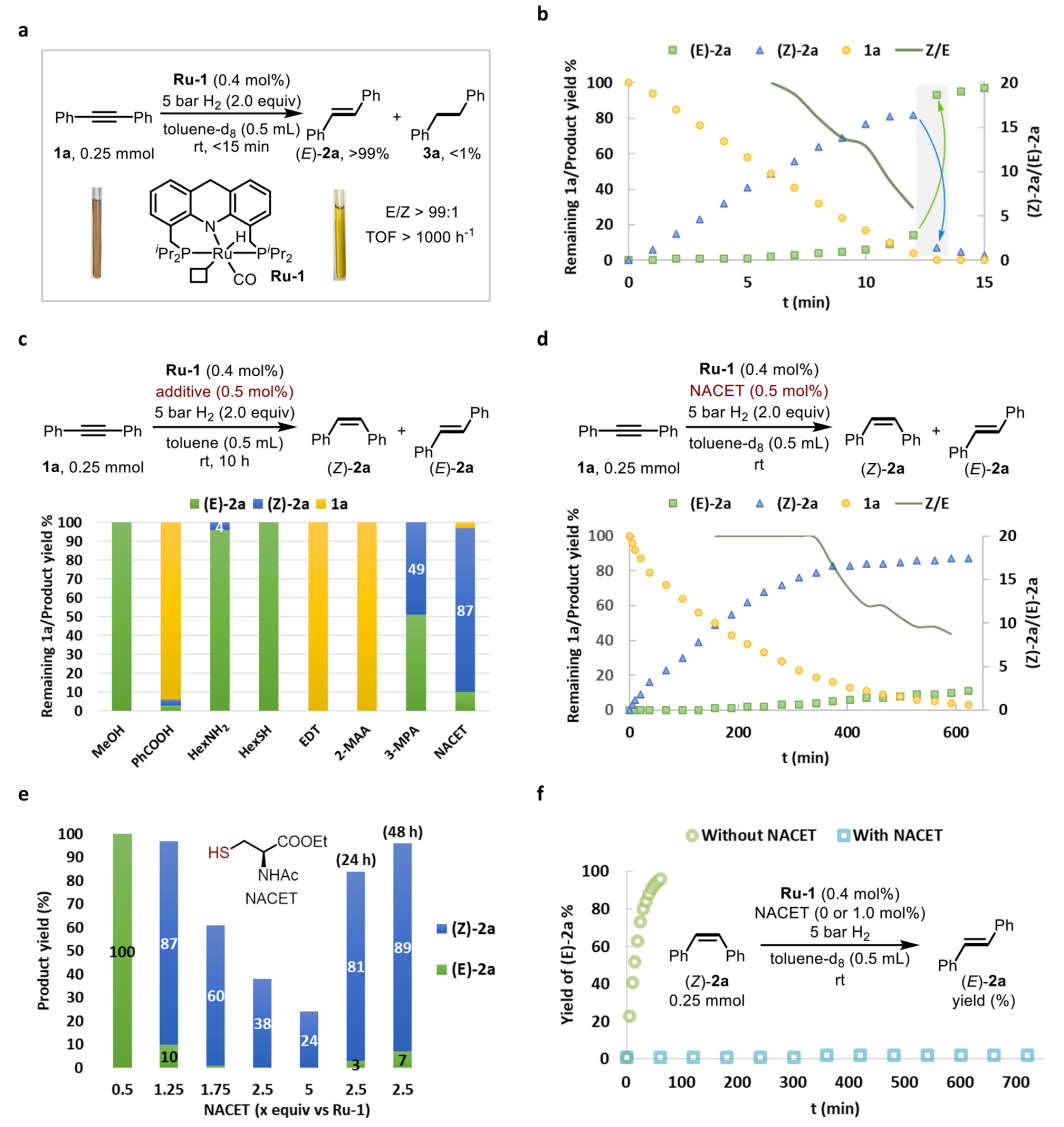
Aspects of controllable *E*/*Z* semihydrogenation of alkynes catalyzed by **Ru-1**. (a) *trans*-Semihydrogenation of **1a** catalyzed by **Ru-1**. (b) Kinetic profile of the *trans*-semihydrogenation of **1a** (the arrows serve to guide the eye through dramatic changes in product yields). (c) Additive screening under conditions similar to (a), with 0.5 mol % additive, 10 h. (d) Kinetic profile of the *cis*-semihydrogenation of **1a** in the presence of 0.5 mol % NACET. (e) Effect of the amount of added NACET on the selectivity of the semihydrogenation reaction under conditions similar to those depicted in (c). (f) Control experiments for the *Z*/*E* isomerization process.

Catalytic semihydrogenation of internal alkynes is an attractive route to access different alkenes for small-scale laboratory synthesis as well as large-scale industrial processes.^8−27,52−76^ Controlling the stereoselectivity and avoiding the formation of over-reduced alkane products are two major challenges facing the development of such reactions. A popular means of addressing these challenges is the commercially available Lindlar catalyst, which has been used for decades to produce (*Z*)-alkenes by *cis*-semihydrogenation of alkynes.^60^ In order to avoid over-reduction into alkanes, this system makes use of Pb(OAc)_2_ as an additive that intentionally poisons the active Pd sites,^76^ but this could raise serious concerns of toxic Pb leaching into the products, especially when food ingredients and pharmaceuticals are involved. Moreover, the application of the Lindlar catalyst is restricted by its limited *cis*-stereoselectivity and catalyst robustness, thus raising the necessity for further catalyst development.^71^ The acridine-based PNP-type ruthenium pincer complex **Ru-1** was found to be a highly active catalyst for the *trans*-semihydrogenation of alkynes and therefore provides a stereocomplementary approach to the Lindlar reduction. For example, in the presence of 0.4 mol % **Ru-1**, 0.25 mmol of diphenylacetylene (**1a**) in 0.5 mL of toluene-*d*_8_ was fully hydrogenated into (*E*)-stilbene within only 15 min at room temperature inside a 3 mL J. Young nuclear magnetic resonance (NMR) tube pressurized with 5 bar of hydrogen gas (2 equiv; Figure 1a). This translates into a TOF of more than 1000 h^–1^, which, to the best of our knowledge, represents the most efficient *trans*-semihydrogenation of any alkyne reported to date.^8−27,52−76^ As the reaction progressed, a clear color change from brown-red to yellow was observed, with the latter being that of **Ru-1**, and this visible change could be used as a reaction indicator.^12^ Notably, only negligible over-reduction into alkane **3a** was observed (<1%), possibly due to the very mild conditions employed.

The progress of the semihydrogenation reaction was monitored by NMR spectroscopy in order to extract the kinetic profiles of the various reaction components (Figure 1b; see the Supporting Information for procedures). Based on the measured data, **1a** was found to be consumed immediately upon introduction of H_2_, with no significant induction period, indicating the facile generation of the catalytically active species in this system. Moreover, (*Z*)-**2a** was observed to accumulate as the kinetic product and then be consumed as the NMR signals of its stereoisomer (*E*)-**2a** began to appear, suggesting that (*E*)-**2a** was being generated from (*Z*)-**2a** by *Z*/*E* isomerization. It should be noted that the rate in which (*E*)-**2a** was generated increased as the reaction progressed, with most of (*Z*)-**2a** being converted into (*E*)-**2a** within a very short interval near the end of the reaction. This presumably occurs after full conversion of the alkyne, thus indicating that the presence of alkyne impedes the isomerization step.^20^ These results also imply that in the absence of alkyne, **Ru-1** is a highly efficient catalyst for *Z*/*E* isomerization.

Given the above observations, it is likely that (*Z*)-alkene isomerization occurs at the vacant site of **Ru-1**, and molecules with higher affinity for the metal center, such as alkynes, are able to block this coordination site and thereby impede the isomerization process. However, the selectivity of the reaction toward (*Z*)-**2a** quickly dropped once alkyne conversion had reached 50% (see the *Z*/*E* curve in Figure 1b) and completely inverted at the end of the reaction.^26^ This prompted us to search for an auxiliary additive that could coordinate to the metal center in a way that would allow the main reaction to take place while impeding the isomerization step, thereby effectively halting the reaction at intermediate (*Z*)-**2a**.

To pursue this idea, we first examined the effect of oxygen-containing additives, such as methanol and benzoic acid (Figure 1c). Employing methanol as either an additive (0.5 mol %) or a solvent resulted in full conversion of **1a** into (*E*)-**2a**, whereas 1.25 equiv of benzoic acid (per catalyst) strongly inhibited the reaction, leading to less than 5% hydrogenation products after 10 h. In the latter case, it was found that a ruthenium–carboxylate complex^77,78^ was easily generated in the reaction mixture, but this species could not effectively activate H_2_ gas to regenerate the ruthenium hydride species (see Figure S24), which may account for the sluggish nature of the catalytic reaction in the presence of benzoic acid. Amines were also examined, since they are expected to bond more tightly to the ruthenium center than oxygen-based additives. Interestingly, when 0.5 mol % of hexylamine (HexNH_2_) was added along with the catalyst, 4% of (*Z*)-**2a** was observed by the end of the reaction, after full conversion of **1a** had been reached. This result supported our assumption that additives with higher metal affinity would improve the yield of the (*Z*)-**2a** intermediate obtained after completion of the reaction. However, further screening of different amine additives failed to improve the yield of (*Z*)-**2a** under similar reaction conditions (see Figure S32 for the screened amines).

Based on these preliminary results, it was decided to explore thiol additives, since they are known to strongly coordinate to Ru(II) centers.^79,80^ Nevertheless, ruthenium thiolate complexes that may be generated during the reaction are expected to readily activate H_2_, thereby regenerating the catalytically active ruthenium hydride species, as we have recently observed.^81^ Initially, 0.5 mol % of 1-hexanethiol (HexSH) was added to the reaction mixture under catalytic conditions similar to the previous experiments, but (*E*)-**2a** still formed as the only product. This indicated that HexSH is compatible with the hydrogenation reaction, but it is not effective enough as a catalyst inhibitor to impede the alkene isomerization process. Thus, various other thiols were screened, showing significant variations in the results. For example, when *N*-decyl 2-mercaptoacetamide (2-MAA) or ethanedithiol (EDT) was used as additives, no alkyne conversion was observed, but when butyl 3-mercaptopropionate (3-MPA) was employed, (*Z*)-**2a** was obtained in 49% yield, with a *Z*/*E* selectivity of 1:1. The different behavior of these thiols, which was not observed in the similarly structured amine additives, may be due to the tethered functional groups of the thiols (see Figure S32). Significantly, the best result was obtained when a cysteine derivative, *N*-acetylcysteine ethyl ester (NACET), was used, allowing us to improve the *Z*/*E* selectivity to 9:1 and achieve a yield of 87% for (*Z*)-**2a**, with 96% alkyne conversion.

To gain further insights into the effect of NACET, we monitored the progress of the catalytic process in the presence of this additive by NMR spectroscopy and constructed the corresponding kinetic profiles (Figure 1d). As indicated by the kinetic curves, despite the presence of thiol in the catalytic mixture, the reaction proceeded smoothly, exhibiting no induction period and reaching high conversion, with 87% of (*Z*)-**2a** being generated within 10 h. Prolonging the reaction time to 20 h had no significant deleterious effect on the yield of (*Z*)-**2a**, with 82% being detected in solution, alongside full conversion of **1a**. It should be noted that alkene isomerization does occur in the presence of thiol, but it is efficiently slowed down during the whole process (see the *Z*/*E* curve in Figure 1d). These results prompted us to investigate another important aspect of the NACET additive, namely, the influence of its amount relative to the catalyst **Ru-1** (Figure 1e). As expected, adding NACET at only 0.5 equiv relative to the catalyst did not prevent the isomerization of (*Z*)-**2a**, resulting in full conversion of **1a** into (*E*)-**2a**. Increasing the relative amount of thiol decreased the hydrogenation rate, as is evident from the significant drop in product yield, and this largely rules out an outer-sphere hydrogenation mechanism, which does not involve alkyne coordination to the metal center (also see Figure S28). At the same time, the selectivity of the reaction greatly benefits from increasing the amount of thiol. Thus, the catalytic reaction employing 2.5 equiv of NACET relative to **Ru-1** gave 84% of **2a** after 24 h, with an excellent *Z*/*E* selectivity of 27:1. Prolonging the reaction time to 48 h improved the yield of **2a** but reduced the selectivity (96%, *Z*/*E* = 13:1).

The effect of NACET on the rate of *Z*/*E* isomerization catalyzed by **Ru-1** was assessed by using (*Z*)-**2a** as the substrate and monitoring its conversion into the (*E*)-isomer in toluene-*d*_8_ under 5 bar of H_2_, in the absence and presence of the thiol, using in situ NMR spectroscopy (Figure 1f). The isomerization rates in these control experiments were slower than those in the actual catalytic runs, possibly because of the easier access of the catalytically active species in the presence of alkyne substrate (see Note S1 for details). Nevertheless, the remarkable effect of thiol-induced inhibition on the isomerization process is clearly apparent. Thus, in the absence of thiol, 96% of (*Z*)-**2a** were converted into (*E*)-**2a** within 1 h, whereas in the presence of 1 mol % NACET, the isomerization rate dropped by a factor of >500, resulting in less than 2% conversion after 12 h. Therefore, this thiol can serve as a highly reliable catalytic inhibitor, which prevents interactions between alkenes and the metal center in this catalytic system.

### Mechanistic Investigations

**Figure 2:**
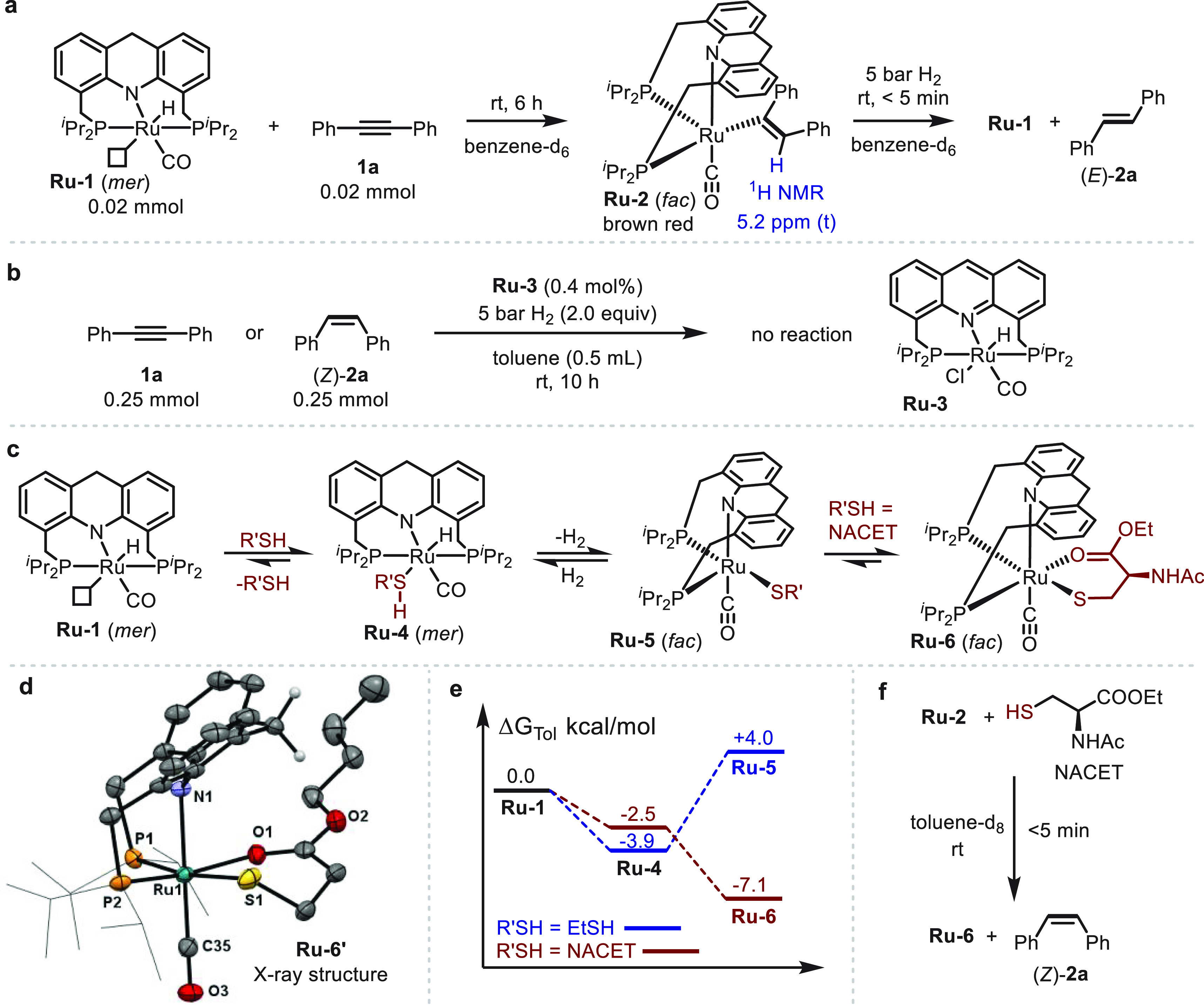
Mechanistic studies involving different steps of the reaction. (a) Generation of a ruthenium alkenyl species. (b) Control experiments using **Ru-3** as the catalyst. (c) Reactions between **Ru-1** and NACET. (d) X-ray crystal structure of **Ru-6′**, with isopropyl groups presented in wireframe style and most hydrogen atoms omitted for clarity. (e) Calculated potential energy surface of different catalytically relevant ruthenium species. (f) Transformation of **Ru-2** to **Ru-6** with NACET.

The catalytic species involved in the controllable *E*/*Z* semihydrogenation described above were investigated in detail, in order to better understand the reaction mechanism. When **Ru-1** was mixed with 1 equiv of alkyne **1a** in benzene or toluene, a new species appeared in the ^31^P{H} NMR spectrum upon standing at room temperature, as the signal belonging to **Ru-1** diminished. This species exhibited a very broad NMR signal ranging from 79 to 105 ppm, which indicates fluxional behavior. Interestingly, when the temperature of the toluene solution was lowered to −40 °C, the broad peak split into two distinct resonances at 76.3 and 95.6 ppm (Figure S10), indicating loss of molecular symmetry. Moreover, no hydride peak was observed for the new species in the ^1^H NMR spectrum, but a triplet at 5.2 ppm was detected, attributable to a vinylic hydrogen atom. These observations are consistent with the structure of the ruthenium alkenyl complex **Ru-2**, featuring a facially coordinated pincer ligand (Figure 2a). It should be noted that generation of this alkenyl species in solution was accompanied by a color change to red-brown, as is also observed during the initial stages of the catalytic reaction (Figure 1a). **Ru-2** was found to be unstable at room temperature in solution, gradually decomposing into a mixture of unidentified species, but in the presence of 5 bar H_2_, this complex quickly converted back into **Ru-1** at room temperature, with concomitant generation of (*E*)-**2a**. These results clearly indicate that the alkenyl species **Ru-2** is a reaction intermediate in the semihydrogenation of alkynes. In an attempt to also investigate the reactivity of **Ru-1** toward alkenes, we mixed this complex with either (*Z*)- or (*E*)-**2a**, but no new species were observed in solution. Instead, the ^31^P NMR spectra of these reaction mixtures only exhibited line broadening of the **Ru-1** peak, and this likely indicates a weak π-interaction between the alkenes and Ru center. In contrast to **Ru-1**, the aromatized complex **Ru-3** does not react with alkyne **1a** at room temperature and shows no catalytic activity vis-à-vis alkyne hydrogenation. Thus, when the semihydrogenation of alkyne **1a** was attempted with **Ru-3** under the same conditions used for **Ru-1**, no reaction was observed. **Ru-3** was also found to be unable to catalyze the *Z*/*E* isomerization of (*Z*)-**2a** (Figure 2b). Furthermore, using other ruthenium catalysts developed by our group for the hydrogenation of polar carbonyl groups, either very poor reactivity or selectivity was observed under the typical alkyne hydrogenation conditions (see Figure S31). These results highlight the unique reactivity of **Ru-1**, which may be ascribed to its readily available vacant site, as well as the increased reactivity of its hydride toward alkynes compared to other catalysts.

The catalytic species generated during alkyne semihydrogenation in the presence of NACET were also monitored by NMR spectroscopy in toluene. No detectable amounts of **Ru-1** or **Ru-2** were found in solution, but a new ruthenium complex was observed as the predominant species. This complex could be obtained independently by mixing **Ru-1** with NACET^43,81−83^ and was characterized by NMR spectroscopy as the ruthenium–hydrido–thiol complex **Ru-4** (exhibiting meridional coordination geometry of the pincer ligand; Figure 2c). When the solution containing this complex was heated at 100 °C for 10 min, a Ru–thiolate complex formed, accompanied by the release of H_2_ gas. However, in contrast to complex **Ru-5**, which contains a monodentate thiolate ligand and was previously reported as the reaction product of **Ru-1** and hexanethiol,^81^ the new thiolate complex, obtained with NACET, was identified as **Ru-6**, in which the thiolate ligand is coordinated in a bidentate mode. Importantly, **Ru-6** was also observed in the actual catalytic runs, albeit in small amounts, implying that it is involved in the reaction (see Figure S26). The exact structure of **Ru-6** was confirmed by X-ray crystallographic analysis of its analogue **Ru-6′**, which was prepared from **Ru-1** and butyl 3-MPA using the same procedure (Figure 2d). Interestingly, the coordinatively saturated **Ru-6** was shown to activate H_2_ at room temperature to generate the ruthenium hydride species *fac*-**Ru-4**,^81^ the facial isomer of **Ru-4**, and this transformation may be essential for ensuring catalytic turnover in our system (Figure S27).

The bidentate coordination of the thiolate ligand in **Ru-6** is expected to stabilize this intermediate. The transformation of **Ru-1** to **Ru-6** was computationally studied by density functional theory calculations using toluene as the model solvent, demonstrating that the generation of **Ru-6** is indeed the overall thermodynamically favorable outcome, to the extent of 7.1 kcal/mol (Figure 2e).^83^ It should also be noted that in a nonpolar solvent such as toluene, **Ru-6** (as well as **Ru-4**) can be further stabilized by hydrogen bonding between the amide groups of the coordinated and free NACET units, as computationally modeled in the gas phase (Figure S86).

In the actual catalysis, the stabilizing effects of NACET can drive the reaction forward to generate **Ru-6**, and may account for the enhanced selectivity observed with NACET as the additive, compared to the other examined thiols (Figure 2e). Moreover, **Ru-6** may also be generated during the catalytic reaction by direct protonation of the alkenyl species **Ru-2** by an incoming thiol, as shown by a control experiment (Figure 2f). This also implies that the acidity of the thiol^84,85^ is essential for quenching **Ru-2** to avoid its hydrogenolysis back to **Ru-1**, which is responsible for the *Z*/*E* isomerization of alkenes (also see Note S2).

### Proposed Mechanism

**Figure 3:**
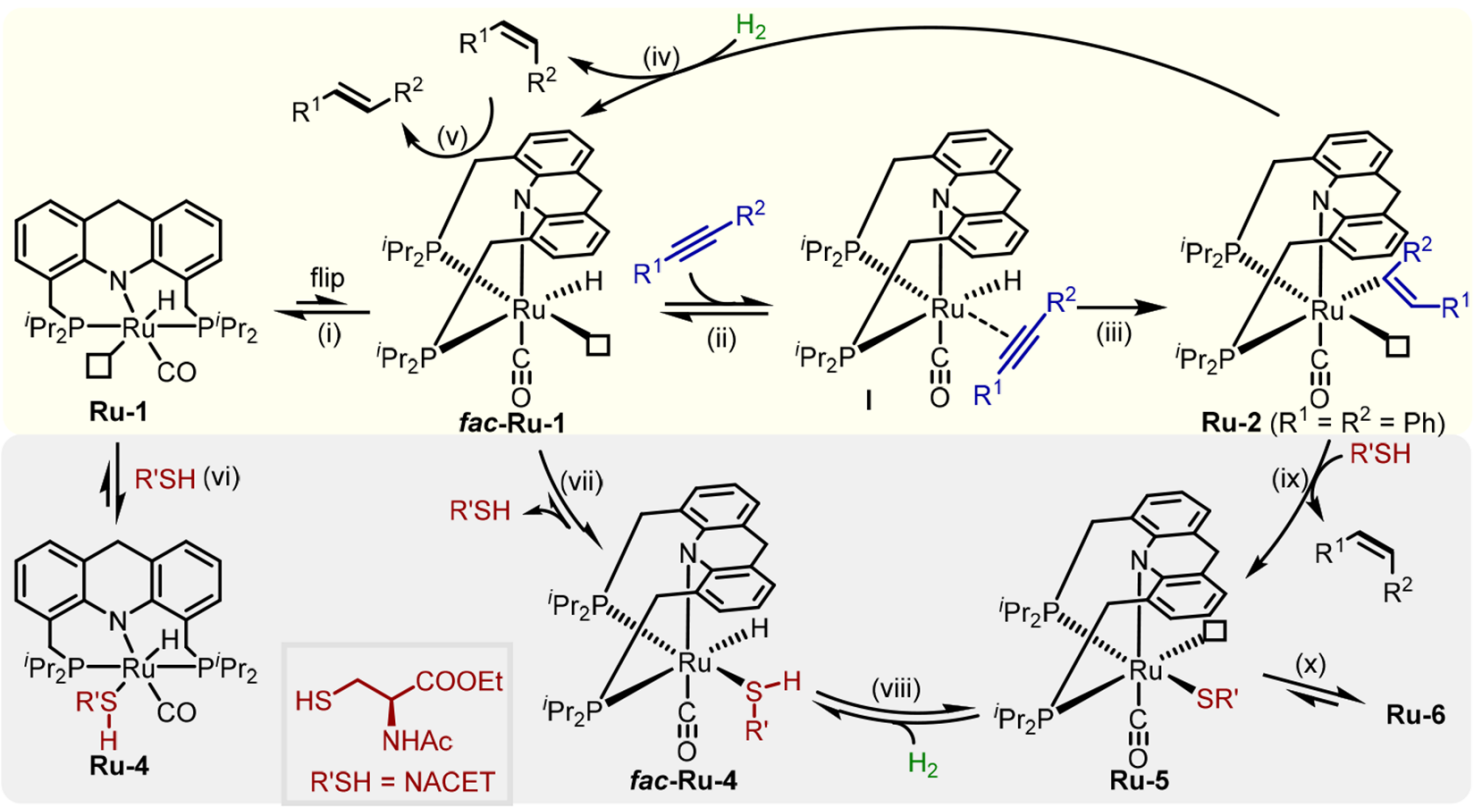
Proposed catalytic cycle for the semihydrogenation of alkynes catalyzed by **Ru-1** in the absence and presence of thiol.

Based on the above results, a mechanism is proposed for the controllable semihydrogenation of alkynes in the current catalytic system (Figure 3). According to our previous studies, as well as the current results (see Notes S1 and S2), *fac*-**Ru-1** is probably the actual catalytically active species, which is generated from **Ru-1** through a ring flip (step i), and exhibits a vacant coordination site *cis* to the hydride ligand.^86,87^ Initially, the alkyne inserts into the Ru–H bond to generate the alkenyl species **Ru-2** (steps ii and iii), which was fully characterized by NMR spectroscopy as part of the control experiments. Subsequently, this species can heterolytically split H_2_ to regenerate the ruthenium hydride species *fac*-**Ru-1** and release the (*Z*)-alkene product (kinetic intermediate, step iv). The latter can then further interact with the ruthenium center to undergo *Z*/*E* isomerization, thereby affording the final (*E*)-alkene product (thermodynamic product, step v). In the presence of thiol, the ruthenium species are poisoned through formation of stable ruthenium thiol(ate) complexes (**Ru-4**, **Ru-5**, and **Ru-6**; steps vi to x). In this manner, the thiol serves as a reversible inhibitor protecting the vacant site on the metal center. The affinity of the alkyne is high enough so that it is able to exchange with the thiol and insert into the Ru–H bond (steps vii, ii, and iii), thereby generating the ruthenium alkenyl species **Ru-2**, which can then be protonated by the thiol to form **Ru-6** along with the release of the (*Z*)-alkene (steps ix and x; see Figure S27 for control experiments). By contrast, the generated alkenes, which have much lower affinities for the metal center than do the alkyne or thiol, cannot significantly interact with the ruthenium center, as verified by the control experiments that directly investigated *Z*/*E* isomerization (Figure 1f). Notably, in the actual catalytic runs, the thiol not only impeded *Z*/*E* isomerization but also slowed down the reaction after significant alkyne conversion had been reached, thus pausing the reaction at the (*Z*)-intermediate stage and leading to excellent selectivity. While the added thiol is responsible for the selectivity of the reaction, its role in promoting H_2_ activation by the ruthenium thiolate complex to regenerate the Ru–H intermediate is also essential to ensure further turnover of the entire catalytic cycle.
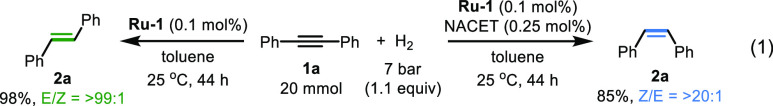
1

### Substrate Scope

**Figure 4:**
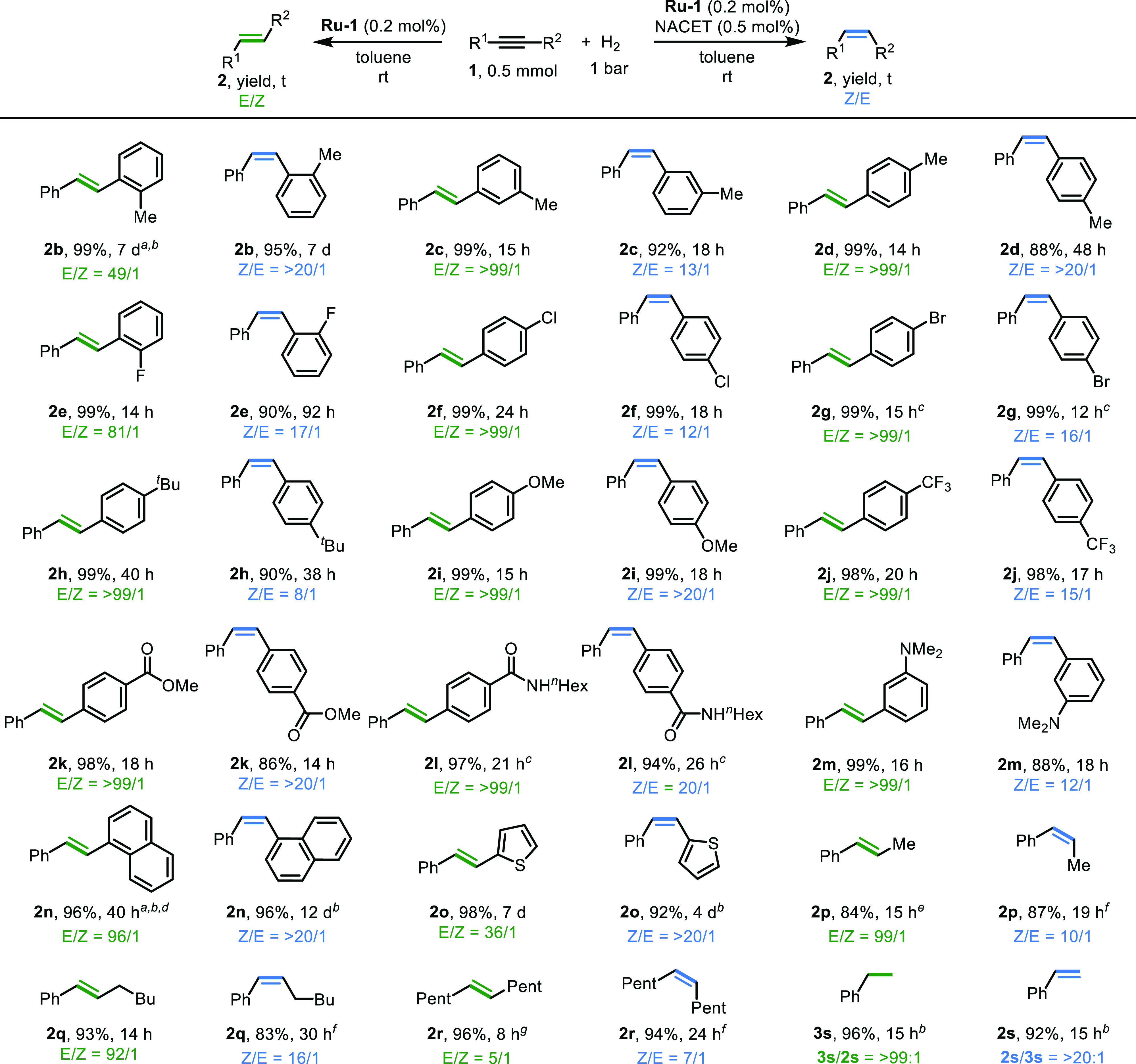
Substrate scope exploration. General conditions: **1** (0.5 mmol), **Ru-1** (0.2 mol %), NACET [0 mol % for (*E*)-isomer; 0.5 mol % for (*Z*)-isomer], toluene (1 mL), H_2_ (1 bar), room temperature. Product ratios and yields were determined by ^1^H NMR spectroscopy using 1,3,5-trimethoxybenezene (0.25 mmol) as an internal standard. Unless otherwise noted, no significant amounts of over-reduced alkane products were observed. ^a^40 °C. ^b^2 bar H_2_. ^c^Tetrahydrofuran as the solvent. ^d^4% alkane product. ^e^14% alkane product. ^f^NACET (1.0 mol %) was used. ^*g*^0 °C.

The practicality of our catalytic system was also examined. First, the procedure was scaled up to involve 20 mmol of alkyne **1a** in a 90 mL Fischer–Porter tube, resulting in the efficient formation of both (*E*)- and (*Z*)-**2a** using only 0.1 mol % of **Ru-1** in the absence and presence of a catalytic amount of the NACET additive, respectively (eq 1). Notably, no significant amounts of alkane **3a** were observed in either case, demonstrating that this system efficiently suppresses the unwanted over-reduction reaction. Subsequently, the substrate scope of this catalytic reaction was explored under very mild conditions, generally using 0.5 mmol of substrate under 1 bar of H_2_ at room temperature and employing 0.2 mol % **Ru-1** (Figure 4; see Table S1 for detailed conditions). It was found that substituents at different positions around the phenyl rings of diphenylacetylene did not affect the results, and the effect of thiol on these stereodivergent semihydrogenations was preserved, thereby allowing excellent selectivities to be achieved (**2b–2d**). An extended reaction time was required for the ortho-methylated substrate **1b**, possibly because of the increased steric hindrance around the triple C≡C bond. Our system was also found to be compatible with a wide range of functional groups. Thus, substituents like halogen atoms, the bulky ^*t*^Bu group, the electron-donating OMe group, the electron-withdrawing CF_3_ group, and even ester, secondary amide, and tertiary amine groups, were all well tolerated (**2e–2m**). Alkynes bearing other aryl moieties, such as naphthalene and thiophene, were also suitable substrates (**2n**, **2o**), albeit requiring extended reaction times. In addition to these diaryl internal alkynes, acetylenic groups with aliphatic substituents were also explored (**1p–1r**). These substrates usually exhibit higher reactivities, possibly due to decreased steric hindrance. Our controllable semihydrogenation methodology was also applied to phenyl acetylene **1s** under similar reaction conditions, resulting in a chemoselective hydrogenation. Interestingly, direct hydrogenation to alkane **3s** was observed in the absence of thiol, whereas addition of a catalytic amount of NACET allowed us to obtain styrene **2s** in >20:1 chemoselectivity. As previously reported, the Lindlar catalyst is not suitable for use with terminal alkynes.^55^ Thus, our current method, which involves switchable selectivity and exhibits excellent substrate compatibility, presents an alternative to Lindlar reduction.

### Controllable Isomerization of Alkenes Using Amine Additives

The current catalytic system was expanded to include the controllable isomerization of alkenes, highlighting the versatility of the reversible inhibition strategy. As mentioned above, our **Ru-1** complex is also a highly efficient catalyst for the isomerization of C=C double bonds. Thus, when 4-phenyl-1-butene **1-2t** was employed as the substrate under the alkene isomerization conditions, in the absence of H_2_ gas (eq 2), **3-2t** was obtained selectively after 48 h, following the C=C bond shift to the 3-position (92% yield, >20:1 for **3-2t/2-2t**).^88^ Interestingly, NACET was found to be too poisonous to allow for efficient isomerization in this case, but the addition of a catalytic amount of HexNH_2_ to **Ru-1** enabled it to selectively catalyze the isomerization of the C=C bond to the 2-position, affording product **2-2t** in 89% yield (>20:1 for **2-2t/3-2t**).
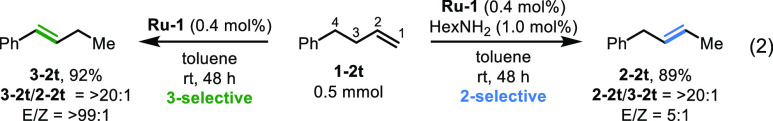
2

## Conclusions

In conclusion, we have introduced a thiol-enabled controllable H_2_-based semihydrogenation of alkynes as a means to afford various alkenes in excellent stereoselectivity using a single catalytic system involving a ruthenium pincer complex as the catalyst. According to this new methodology, the thiol serves as a switch that adjusts the selectivity of the system, such that the absence of thiol results in the *trans*-selective semihydrogenation of internal alkynes to give (*E*)-alkenes, whereas addition of thiol easily halts the reaction at the (*Z*)-alkene intermediate. Our mechanistic study indicates that the presence of thiol prevents the isomerization of alkenes by blocking the vacant coordination site on the catalyst, while selectively allowing the main process of alkyne hydrogenation to take place through generation of the ruthenium thiol(ate) catalyst. This bioinspired strategy of reversibly protecting the vacant site on the metal center to achieve catalysis with switchable selectivity is expected to have further applications in both homogeneous and heterogeneous catalyses.

## Supporting Information Available

Control experiments, experimental procedures, NMR spectra, and computational details (PDF)
