# Supplementary material for: Controlled Selectivity through Reversible Inhibition of the Catalyst: Stereodivergent Semihydrogenation of Alkynes
Source: J Am Chem Soc. 2022 Jul 15;144(29):13266–75. doi: 10.1021/jacs.2c04233 (PMC9374179; doi:10.1021/jacs.2c04233)
Supplement: Supplementary file 1 — ja2c04233_si_001.pdf [file ja2c04233_si_001.pdf]

Controlled Selectivity through Reversible Inhibition of the Catalyst: Stereodivergent Semihydrogenation of Alkynes

Jie Luo,[‡] Yaoyu Liang,[‡] Michael Montag, Yael Diskin-Posner,[§] Liat Avram,[§] David Milstein*

[‡]These authors contributed equally to this work.

Department of Molecular Chemistry and Materials Science and §Department of Chemical Research Support, Weizmann Institute of Science, Rehovot, 76100, Israel

E-mail: david.milstein@weizmann.ac.il

Table of Contents

Supplemental Notes	S2
Supplemental Experimental Procedures	S5
1. General considerations	S5
2. Characterization of ruthenium complexes	S6
3. Control experiments	S23
4. General experimental procedures	S29
5. Selected crude NMR spectra	S40
6. Characterization of substrate **1l**	S64
7. Single crystal X-ray diffraction analysis	S66
8. Computational details	S67
Supplemental References	S92

Supplemental Notes

Note S1. Different rates of Z-E isomerization

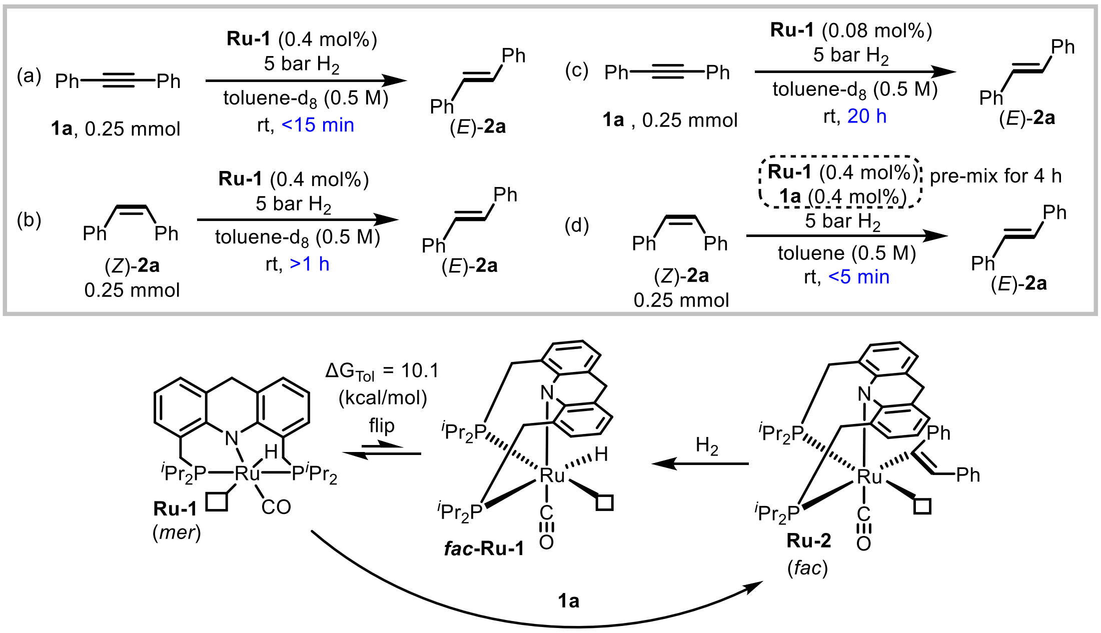

Figure S1. Different rates of Z-E isomerization process.

Different Z-E isomerization rates were observed under different conditions without thiol. The whole actual catalytic reaction, which contains the Z-E isomerization process, only lasted <15 min (Figure S1, eq. a) but the independent test of Z-E isomerization starting from (*Z*)-**2a** took more than 1 h under the similar conditions (Figure S1, eq. b). In addition, decreasing the catalyst loading to 1/5 (0.08 mol%) greatly slowed down the reaction (more than 80 times), mainly because of the slower isomerization process (Figure S1, eq. c).

It is proposed that the concentration of *fac*-**Ru-1** in the system, which is the actual catalytic species, causes the different isomerization rates. In the actual catalysis, the catalyst **Ru-1** (*mer*) fully converted into ruthenium alkenyl species **Ru-2** (*fac*) promoted by the large excess amounts of alkyne substrate, which underwent further hydrogenolysis to generate *fac*-**Ru-1**. Such high concentration of *fac*-**Ru-1** quickly catalyzed the isomerization process of the alkene before it flipped back to **Ru-1** (*mer*). In the control experiment of the isomerization test (Figure S1, eq. b), only a small

percentage of *fac*-**Ru-1** was generated from the flip of **Ru-1** (*mer*) ($\Delta G_{mer\ \mathrm{to}\ fac\ \mathrm{in\ Tol}}$ = 10.1 kcal/mol), thus the isomerization rate decreased compared to the actual catalysis involving alkynes.

To verify this proposal, another control experiment of Z-E isomerization was carried out but in the presence of a catalytic amount of **1a** (Figure S1, eq. d, premix with **Ru-1** for 4 h). Expectedly, the catalysis was complete within 5 min (color change to yellow occurred within <10 s). These results support the proposal that the available concentration of *fac*-**Ru-1** affected the isomerization rate.

Note S2. The Z-E isomerization process in the presence of thiol in actual catalysis

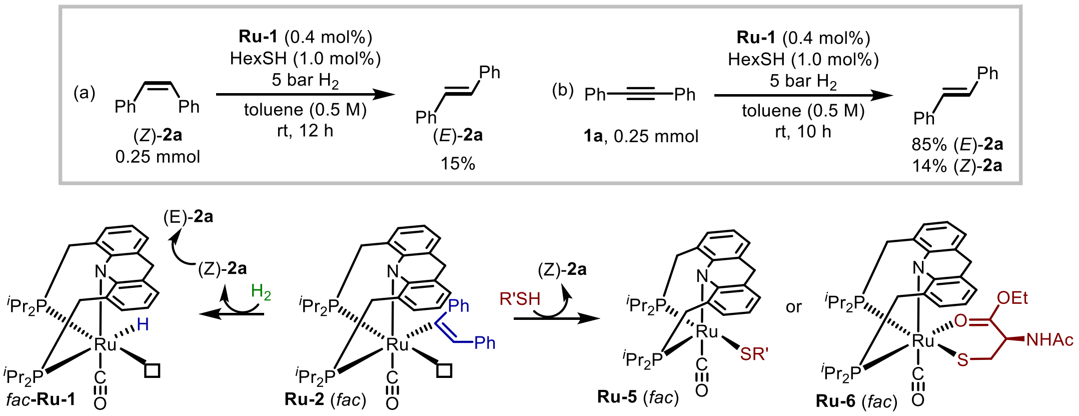

Figure S2. Z-E isomerization process in actual catalysis.

It was found that hexanethiol impeded the rate of Z-E isomerization efficiently as well in an independent control experiment (Figure S2, eq. a). However, in the actual catalysis, it seems that the presence of hexanethiol didn't protect (Z)-**2a** well from isomerization (Figure S2, eq. b). An explanation for these contradictory results is that most of Z-E isomerization happened during the hydrogenation of the alkyne, that is, before the full consumption of the alkyne substrate. In the presence alkyne, *fac*-**Ru-1** is easier to form through the hydrogenolysis of alkenyl species **Ru-2** to conduct the quick Z-E isomerization catalysis (see proposal in Note S1 that *fac*-**Ru-1** after ring flip is the actual catalyst). Thus, if the alkenyl species **Ru-2** couldn't be quickly quenched by thiols, *fac*-**Ru-1** might survive for a relative long time during the catalysis, catalyzing the generation of (E)-**2a** through Z-E isomerization (Figure S28).

These results also support our proposal in the manuscript as to why NACET functioned differently from HexSH. In the actual catalysis in the presence of thiols, the generation of *fac*-**Ru-1** by hydrogenolysis competes with the generation of ruthenium thiolate complexes **Ru-5** or **Ru-6** from the alkenyl species **Ru-2**. Thus, in the case of NACET, the thermo-stability of the thiolate complex **Ru-6** ($\Delta\Delta G_{Tol}$ = -11.1 cal/mol compared to **Ru-5** with EtSH) and the acidity of NACET (pKa ~8.2 *vs* 9.4 of 3-MPA *vs* 10.6 of HexSH) can promote the transformation of alkenyl species **Ru-2** to **Ru-6** instead of *fac*-**Ru-1**, and thus impedes the Z-E isomerization during the reaction.

Supplemental Experimental Procedures

1. General considerations

All experiments with metal complexes and phosphine ligands were carried out under an atmosphere of purified nitrogen in a Vacuum Atmosphere glovebox equipped with a MO 40-2 inert gas purifier or using standard Schlenk techniques. All solvents were reagent grade or better. All non-deuterated solvents were purified according to standard procedures under argon atmosphere. Deuterated solvents were degassed with argon and directly used. All solvents were degassed with argon and kept in the glove box over 3Å molecular sieves. All ^{1}H NMR, ^{13}C NMR or ^{31}P NMR spectra were recorded on a Bruker AVANCE III 300MHz, 400MHz and AVANCE III HD 500 MHz NMR spectrometer and reported in ppm (δ). Chemical shifts were referenced to the residual solvent peaks (^{1}H NMR, ^{13}C NMR) or an internal standard (1,3,5-trimethoxybenzene) or an external standard of phosphoric acid (85% solution in D_2O) at 0.0 ppm (^{31}P NMR). NMR spectroscopy abbreviations: br, broad; s, singlet; d, doublet; t, triplet; q, quartet; m, multiplet. GC-MS was carried out on HP 6890 / HP 5973 (MS detector) instruments equipped with a 30 m column (Restek 5MS, 0.32 mm internal diameter) with a 5% phenylmethylsilicone coating (0.25 mm) and helium as carrier gas. IR spectra were recorded on Thermo Nicolet 6700 FT-IR. Analytical TLC was performed on Merck silica gel 60 F254 plates. Room temperature indicated in this work ranges from 18 ^{o}C to 24 ^{o}C.

Complexes **Ru-1**,[1] **Ru-4**,[2] **Ru-5**,[2] thiols 2-MAA,[3] NACET[4] were prepared according to literature procedures. Alkynes used in this reaction are either commercial or prepared by the coupling of aryl iodides with the corresponding terminal alkynes (including unreported **1l**).[5]

The E/Z stereoselectivity of the crude reaction mixture was analyzed by GC-MS, and further confirmed based on the NMR integrals of two products. The integrals of single peaks (without overlap) in NMR were corrected by auto linear correction in Mestnova and the peaks of non-deuterated toluene in some cases are not fully displayed (measuring parameters: d1 = 30 s, NS ≥ 2).

2. Characterization of ruthenium complexes

Generation of alkenyl species Ru-2

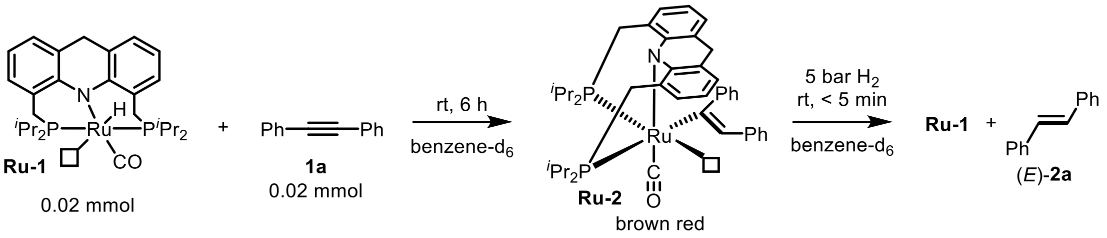

To a J. Young NMR tube was added complex **Ru-1** (11.4 mg, 0.02 mmol), alkyne **1a** (3.6 mg, 0.02 mmol) and benzene-d_6 (0.5 mL) in a N_2 glovebox. Immediate analysis of the sample indicated that $^{31}P\{^1H\}$ NMR signal of the complex broadened, but its chemical shift did not change, likely indicating a weak π-interaction between the alkyne and ruthenium center. The NMR tube was rotated at room temperature for 6 h, with the color changing from orange to red brown. A new species **Ru-2** was generated as the major species at this point with little (*Z*)-**2a** observed by ^1H NMR. **Ru-2** was not stable, and after NMR measurements (12 h), some decomposition was observed along with the increased signal of (*Z*)-**2a**. The generation of (*Z*)-**2a** may come from the deprotonation of the ligand by the Ru-C(sp^2) bond. Then the J. Young NMR tube was pressurized with 5 bar of H_2. A quick color change from red brown back to orange was observed with the regeneration of **Ru-1** in the solution, as verified by NMR.

Due to the fluxional behavior, the phosphorus signal of **Ru-2** is very broad (79.3-104.6 ppm). **Ru-2** was proposed as a *fac*-isomer with double bond in (Z)-configuration according to that (i) VT-NMR showed two different phosphorus peaks at low temperature (*vide infra*); (ii) (*Z*)-alkene was generated initially in the system.

Ru-2: ^1H NMR (400 MHz, C_6D_6) δ 7.14 – 7.08 (m, 2H, ArH), 7.06 – 6.94 (m, 10H, ArH), 6.94 – 6.83 (m, 4H, ArH), 5.21 (t, *J* = 3.1 Hz, 1H, Ru-C=C*H*), 3.50 (q, *J* = 17.0 Hz, 2H, ArCH_2Ar), 2.96 (t, *J* = 12.7 Hz, 2H, ArCH_2P), 2.53 (dd, *J* = 12.1, 6.6 Hz, 2H, ArCH_2P), 2.27 – 2.12 (m, 2H, PC*H*), 1.99 (br, 2H, PC*H*), 1.27 – 0.96 (m, 24H, PCH(CH_3)$_2$). ^{13}C NMR (101 MHz, C_6D_6) δ 204.50 (t, *J* = 14.9 Hz, Ru-*C*O), 155.27 (s,

ArC), 134.51 (s, ArC), 131.94 (s, ArC), 131.13 (s, ArC), 130.67 (s, ArC), 129.30 (s, ArC), 128.64 (s, ArC), 127.70 (s, ArC), 126.70 (s, ArC), 126.39 (s, ArC), 125.20 (s, ArC), 124.17(s, Ru-C=*C*H), 123.18 (s, ArC), 122.48 (s, Ru-*C*=CH), 118.45 (s, ArC), 34.63 (s, ArCH_2P), 29.06 (d, *J* = 18.5 Hz, ArCH_2P), 28.73 (located by HSQC, P*C*H), 20.99 (d, *J* = 4.6 Hz, PCH(CH_3)$_2$), 20.23 (s, PCH(CH_3)$_2$), 19.09 (s, PCH(CH_3)$_2$), 18.53 (d, *J* = 6.1 Hz, PCH(CH_3)$_2$). ^{31}P NMR (162 MHz, $CDCl_3$) δ 104.57 – 79.30 (m).

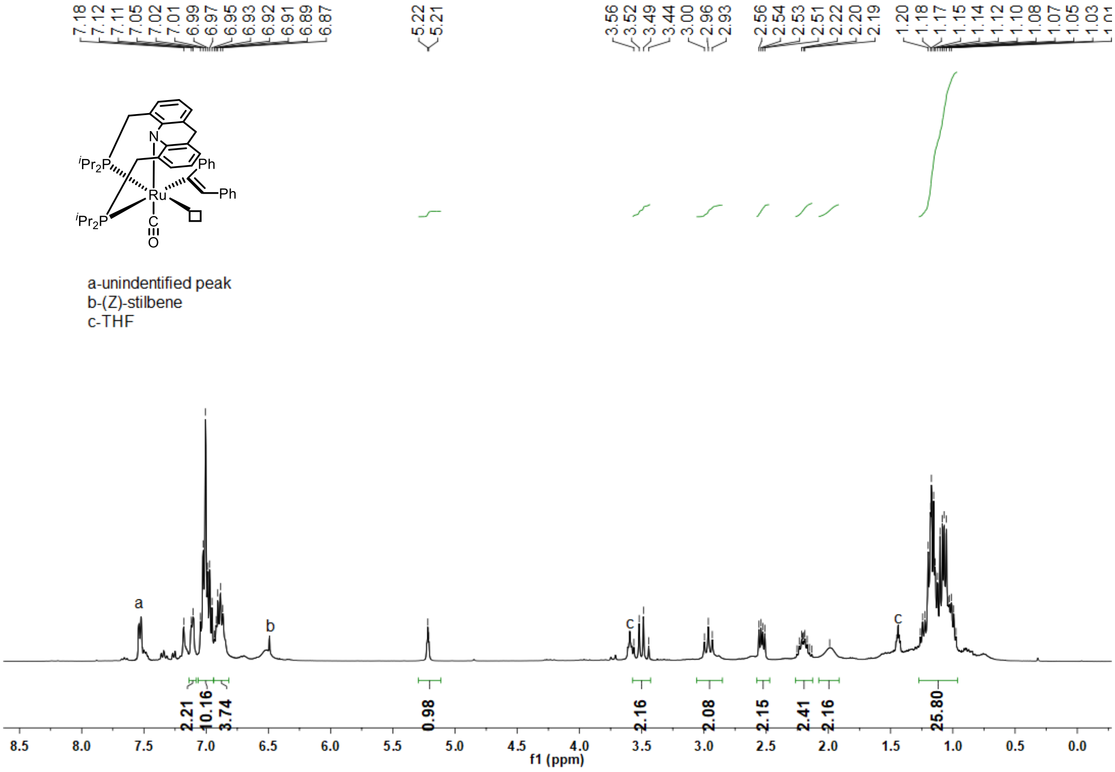

Figure S3. ^{1}H NMR spectrum of the **Ru-2** C_6D_6.

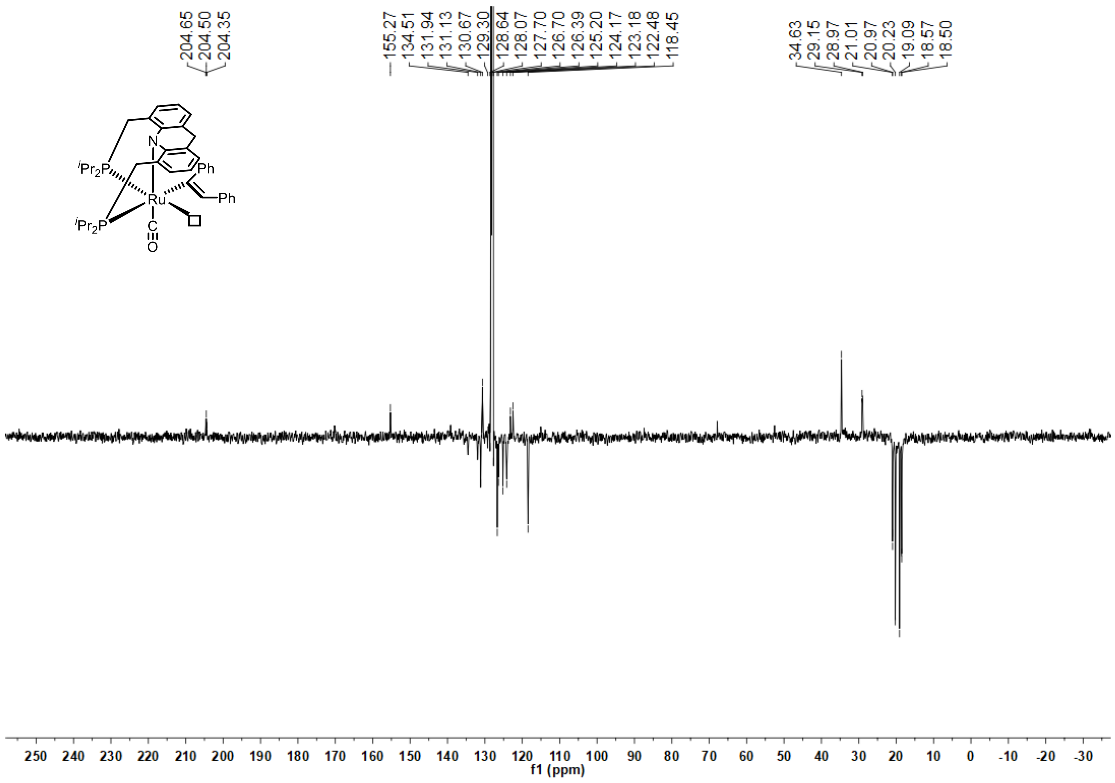

Figure S4. ^{13}C-DEPTQ NMR spectrum of **Ru-2** in C_6D_6.

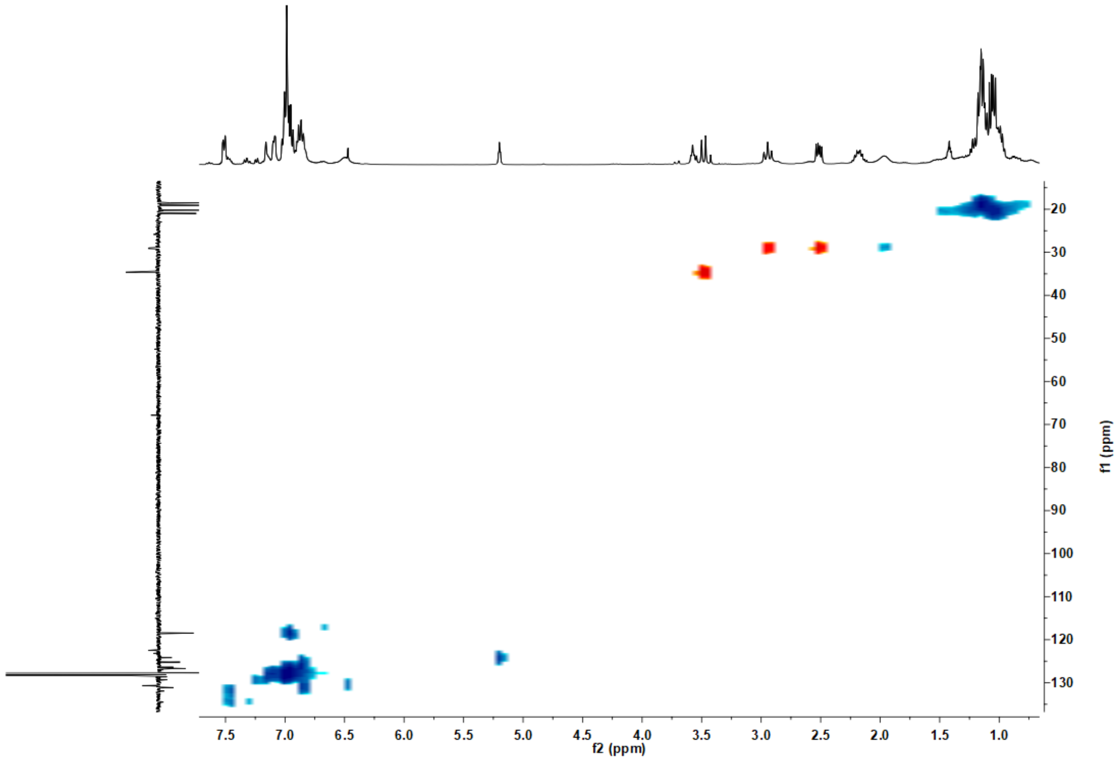

Figure S5. HSQC spectrum of **Ru-2** in C_6D_6.

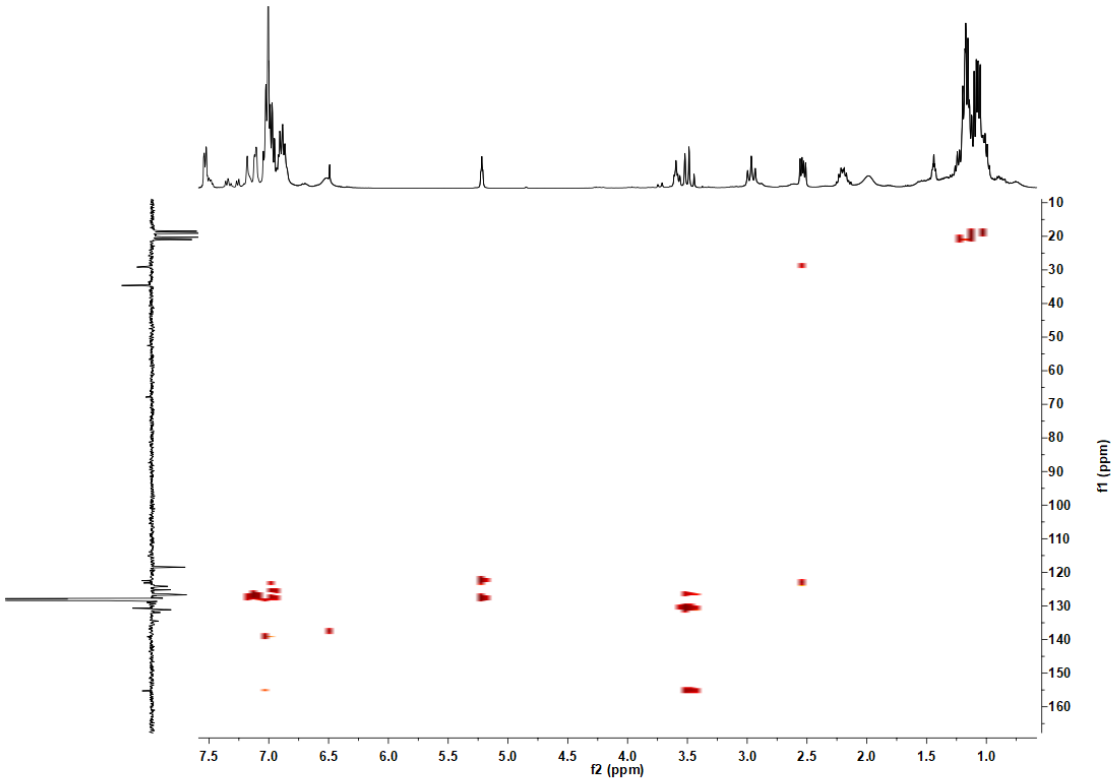

Figure S6. HMBC spectrum of **Ru-2** in C_6D_6.

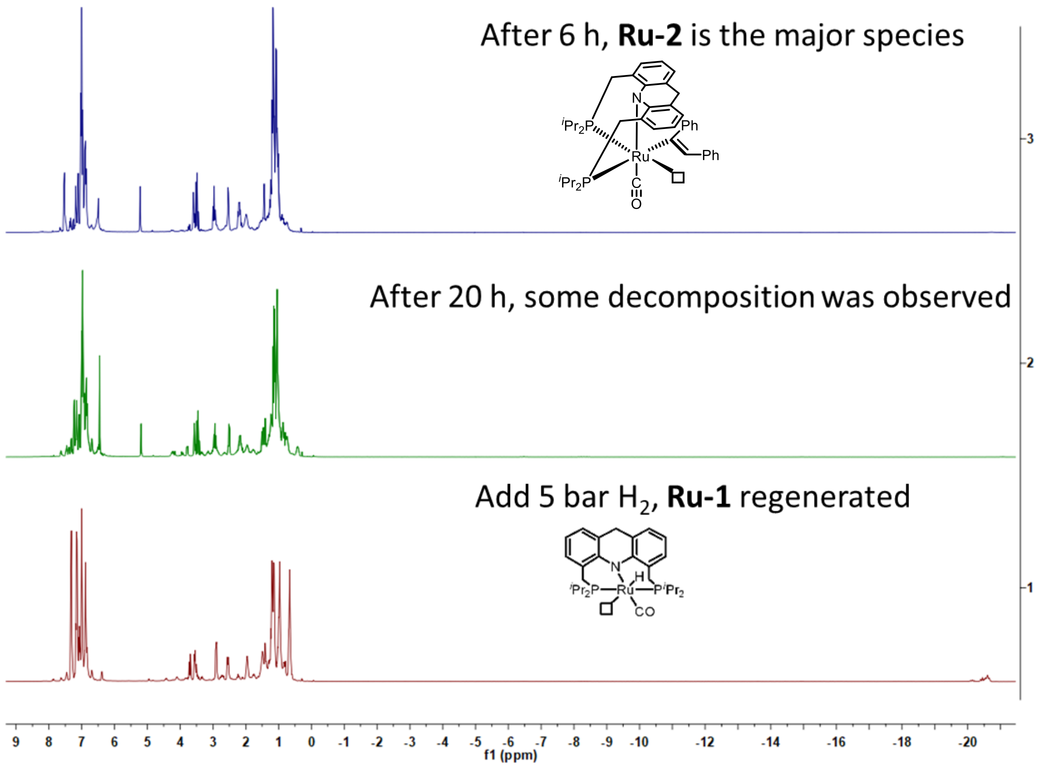

Figure S7. Decomposition of **Ru-2** and its conversion to **Ru-1** under H_2 (^{1}H NMR).

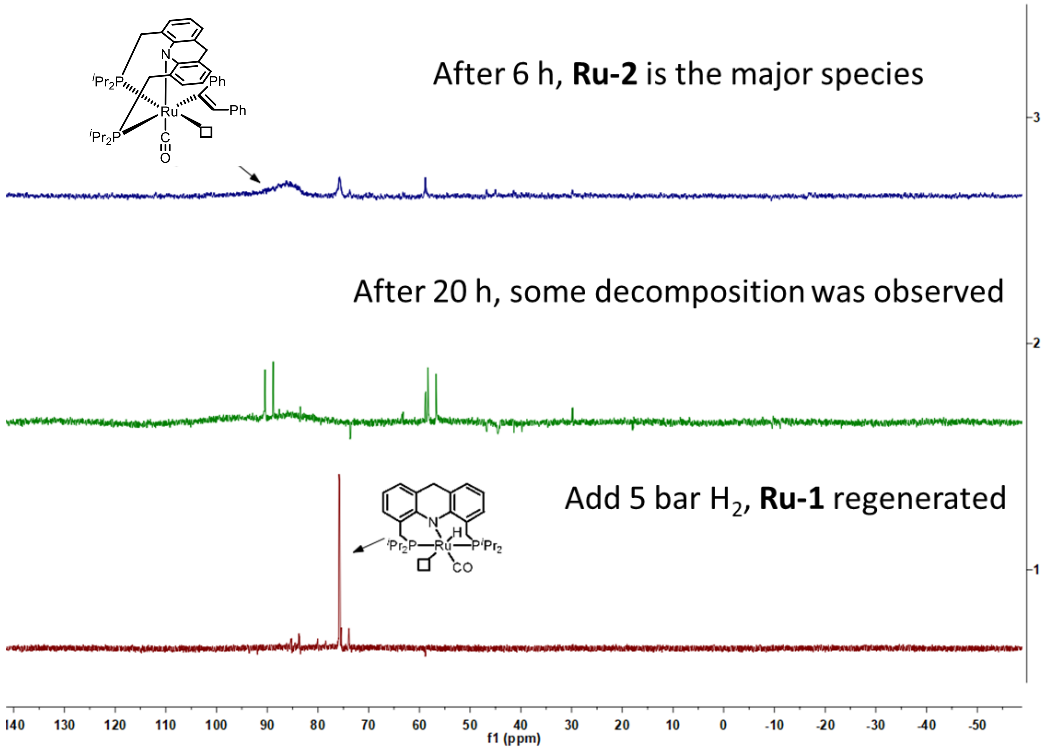

Figure S8. Decomposition of **Ru-2** and its conversion to **Ru-1** under H_2 (^{31}P NMR).

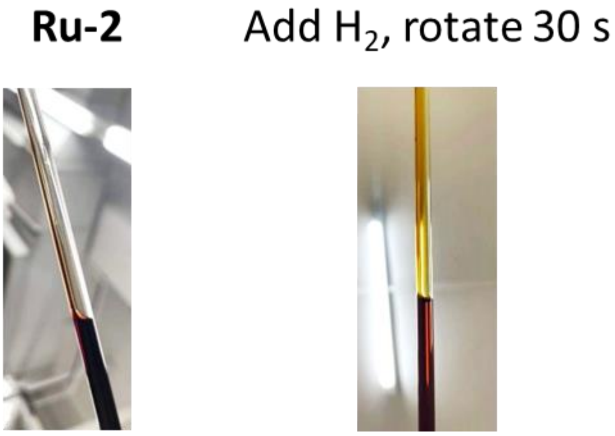

Figure S9. Evident color change from red brown to orange yellow upon the addition of H_2 pressure.

VT-NMR of alkenyl species Ru-2 and its reaction with NACET

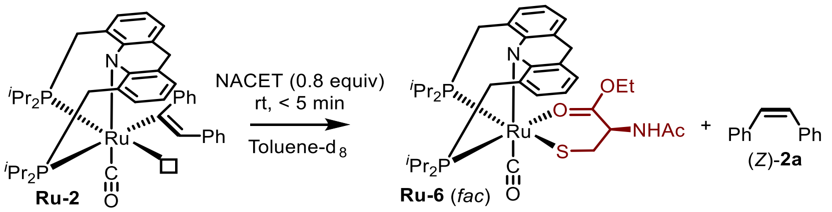

To a J. Young NMR tube was added complex **Ru-1** (11.4 mg, 0.02 mmol), alkyne **1a** (3.6 mg, 0.02 mmol) and toluene-d_8 (0.5 mL) in a N_2 glovebox. The NMR tube was rotated at room temperature for 6 h, and then measured at rt, 0 °C, -20 °C, -40 °C, and rt again. Two different phosphorus peaks were observed upon lowering the measuring temperature, which supported the *fac*-conformation of the complex. After the measurement, the J. Young NMR tube was charged with NACET (0.8 equiv) and the resulting solution was analyzed by NMR, indicating that the majority of **Ru-2** transformed into **Ru-6** along with the accumulation of (Z)-**2a**.

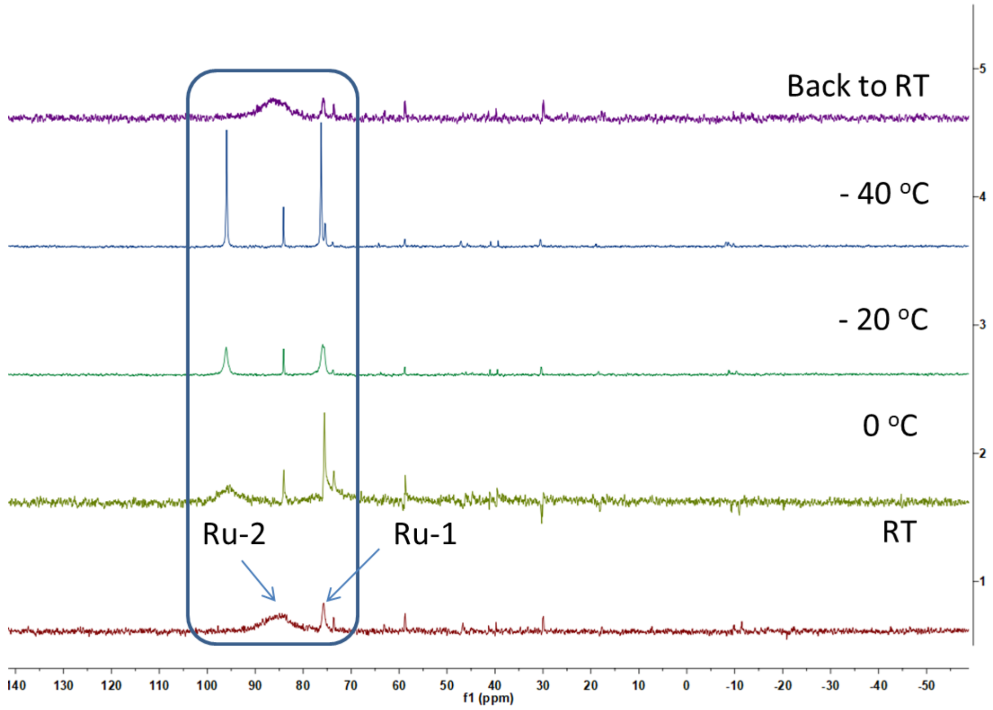

Figure S10. VT-NMR of ruthenium alkenyl species **Ru-2** (^{31}P NMR).

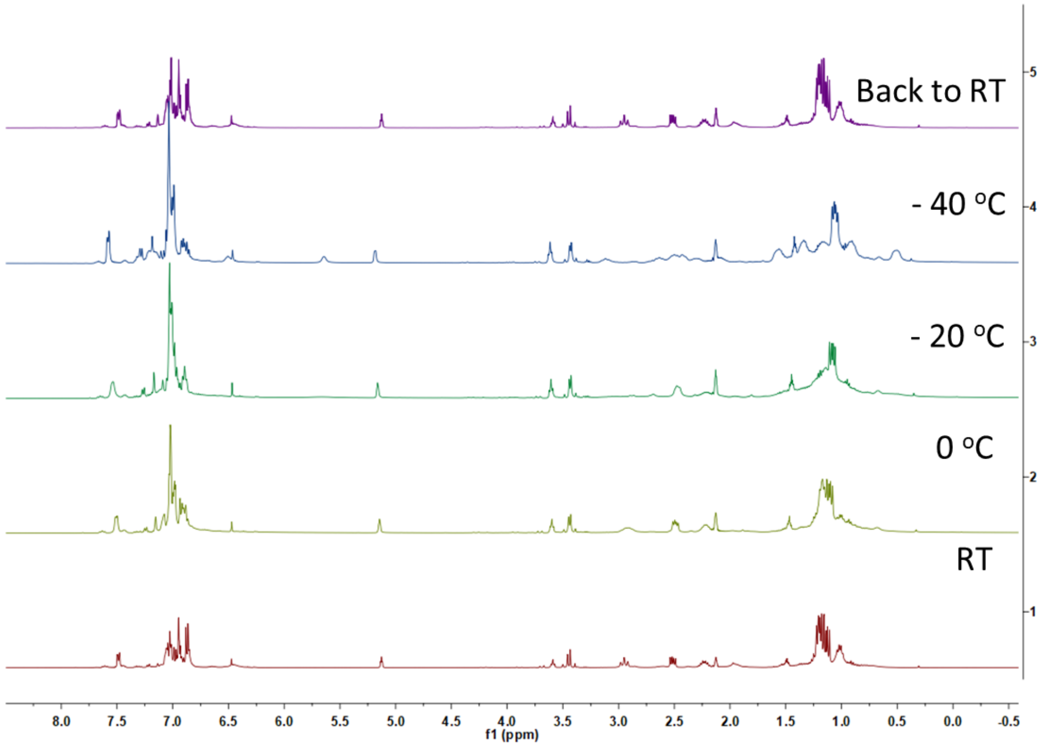

Figure S11. VT-NMR of ruthenium alkenyl species **Ru-2** (^{1}H NMR).

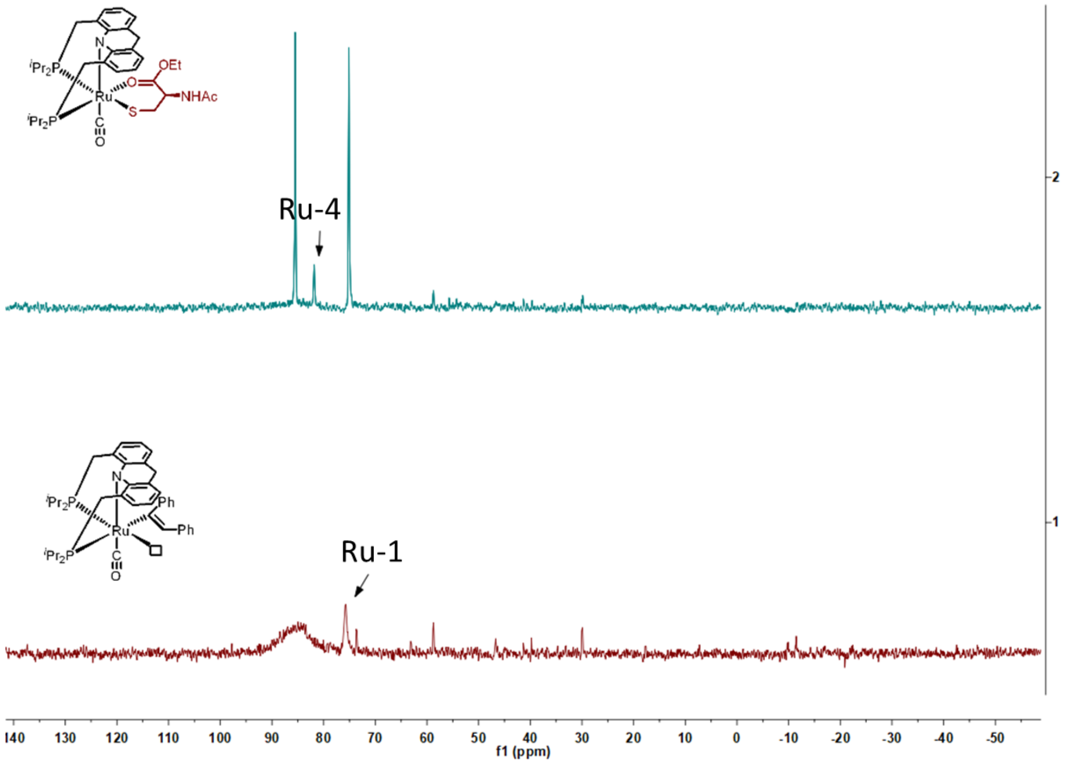

Figure S12. Conversion of alkenyl species **Ru-2** to **Ru-6** upon the addition of NACET (^{31}P NMR; **Ru-4** was from unreacted **Ru-1**).

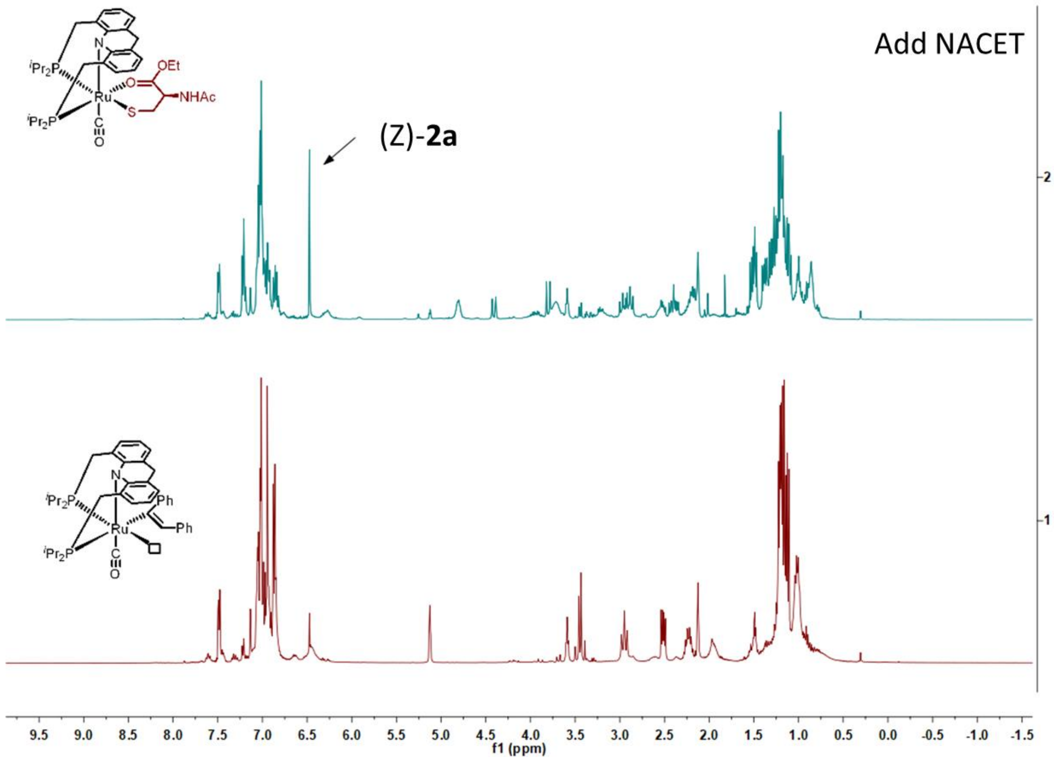

Figure S13. Conversion of alkenyl species **Ru-2** to **Ru-6** and (Z)-**2a** upon the addition of NACET (^{1}H NMR).

Preparation of Ru-4 and Ru-6

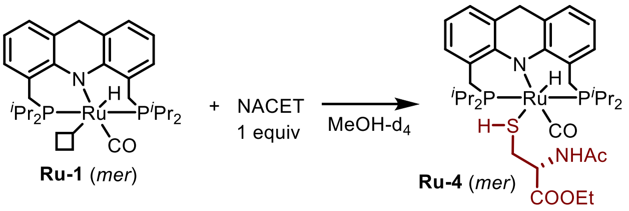

To a J. Young NMR tube was added complex **Ru-1** (11.4 mg, 0.02 mmol), NACET (3.8 mg, 0.02 mmol) and MeOH-d_4 (0.5 mL) in a N_2 glovebox. The NMR tube was rotated at room temperature for 2 h (it takes time to dissolve **Ru-1** in MeOH), with the color changing from orange to light green. NMR analysis indicated the generation of **Ru-4**, in accordance with our previous observations.[2] Due to the existence of the chiral center in the complex, multiple couplings were observed.

Ru-4: IR (cm^{-1}): 2939, 1907, 1742, 1660, 1030. ^{1}H NMR (400 MHz, MeOD) δ 7.13 – 6.95 (m, 6H, ArH), 4.62 (d, *J* = 18.5 Hz, 1H, ArCH_2Ar), 3.99 (q, *J* = 7.0 Hz, 2H, COOCH_2CH$_3$), 3.91 – 3.87 (m, 1H,C*H*NHAc), 3.81 (d, *J* = 18.1 Hz, 1H, ArCH_2Ar), 3.28 – 3.19 (m, 2H, CH_2P), 2.61 – 2.49 (m, 2H, PC*H*(CH$_3$)$_2$), 2.19 (d, *J* = 9.2 Hz, 1H, N*H*), 1.85 (s, 3H, NHCOCH_3), 1.83 – 1.78 (m, 1H, S*H*), 1.35 – 1.01 (m, 29H, PCH(CH_3)$_2$, PC*H*(CH$_3$)$_2$, COOCH$_2$CH_3), -11.84 (t, *J* = 28.3 Hz, 1H, Ru-H). ^{13}C NMR (101 MHz, MeOD) δ 209.47 (t, *J* = 14.5 Hz, Ru-CO), 173.50 (s, *C*OOEt), 173.15 (s, NHCO), 141.45 (dd, *J* = 22.8, 5.0 Hz), 129.82 (d, *J* = 3.1 Hz, ArC), 128.51 (d, *J* = 26.3 Hz, ArC), 128.34 (d, *J* = 14.9 Hz, ArC), 127.12 (s, ArC), 125.55 (s, ArC), 61.84 (s, COO*C*H$_2$CH$_3$), 58.12 (s, *C*HNH), 34.16 (s, Ar*C*H$_2$Ar), 32.38 (ddd, *J* = 54.0, 12.9, 5.3 Hz, *C*H$_2$P), 27.61 (ddd, *J* = 40.7, 22.5, 8.4 Hz, P*C*H(CH$_3$)$_2$), 26.29 (t, *J* = 5.8 Hz, PCH(*C*H$_3$)$_2$), 25.79 (t, *J* = 6.1 Hz, PCH(*C*H$_3$)$_2$), 22.31 (s, NHCO*C*H$_3$), 22.23 (d, *J* = 27.3 Hz, PCH(*C*H$_3$)$_2$), 20.62 (ddd, *J* = 25.1, 5.9, 2.3 Hz, P*C*H(CH$_3$)$_2$), 20.10 (d, *J* = 25.3 Hz, PCH(*C*H$_3$)$_2$), 16.85 (dd, *J* = 6.2, 4.8 Hz, PCH(*C*H$_3$)$_2$), 14.56 (s, COOCH$_2$*C*H$_3$). ^{31}P NMR (162 MHz, MeOD) δ 81.38 (d, *J* = 34.8 Hz).

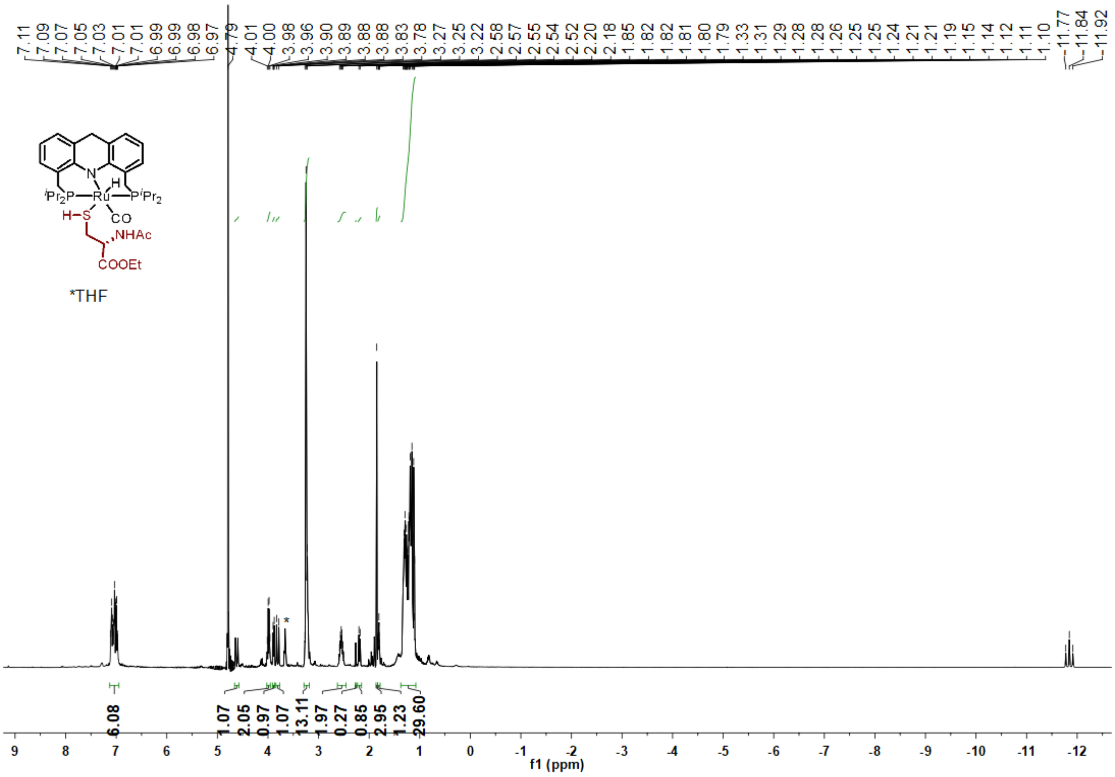

Figure S14. ^{1}H NMR spectrum of **Ru-4** in CD_3OD.

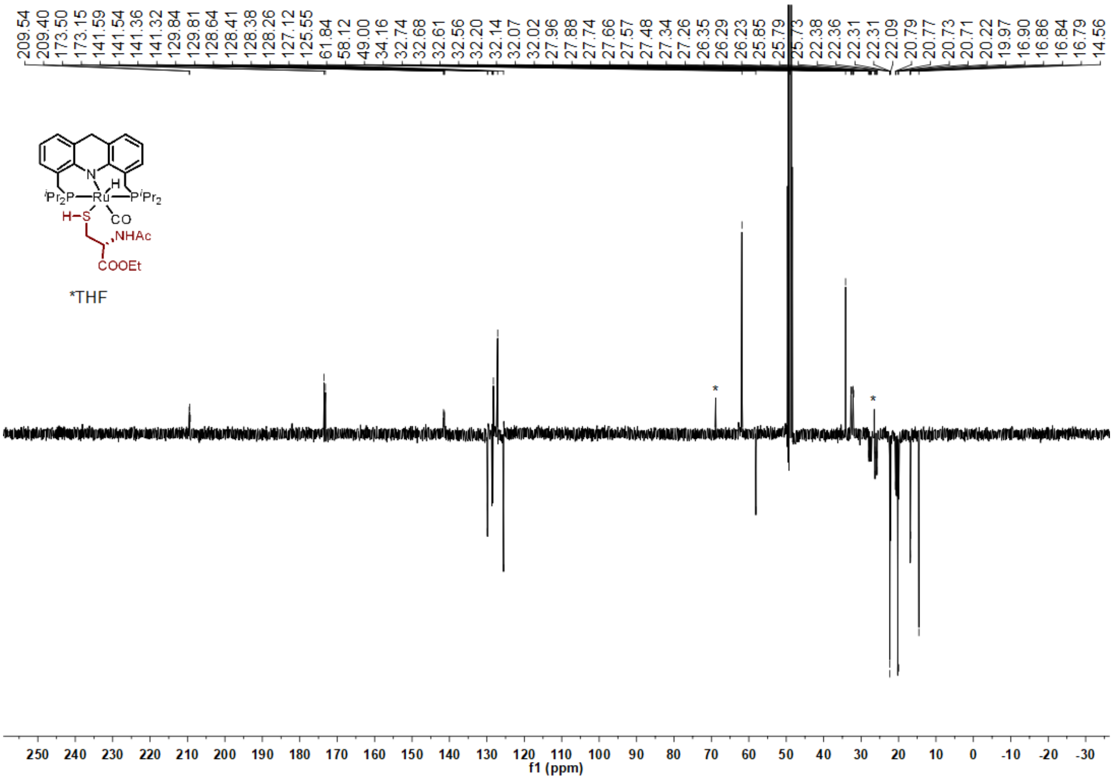

Figure S15. ^{13}C-DEPTQ NMR spectrum of **Ru-4** in CD_3OD.

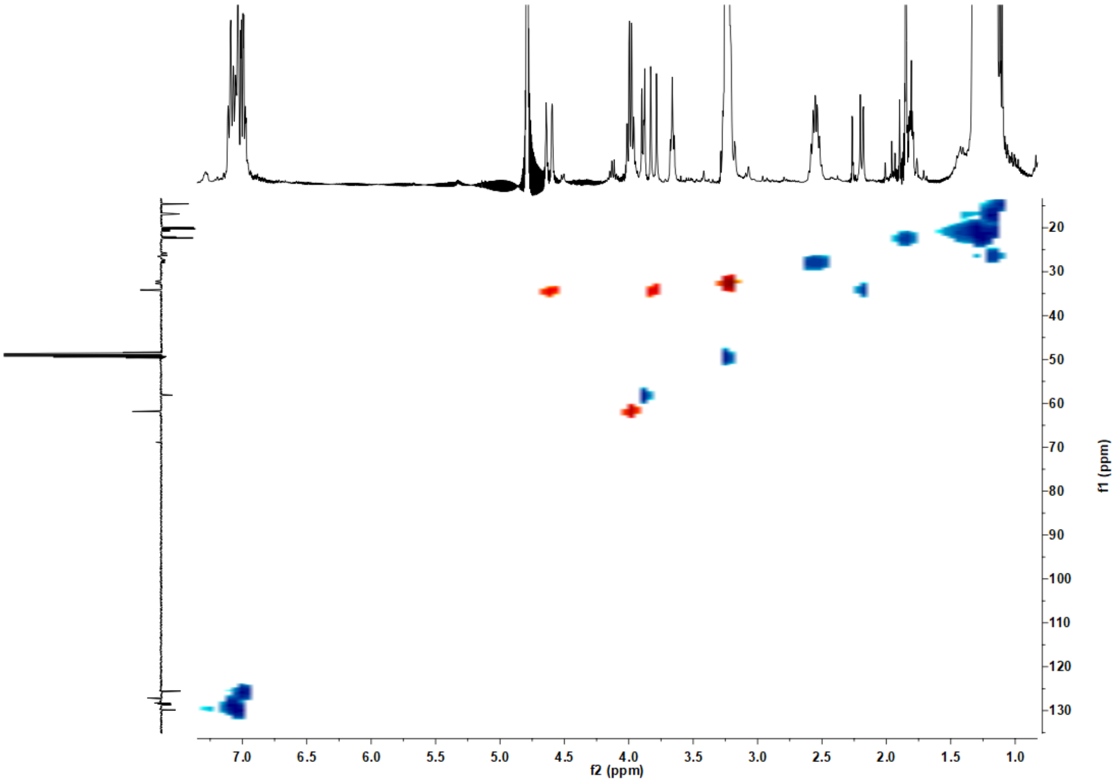

Figure S16. HSQC spectrum of **Ru-4** in CD_3OD.

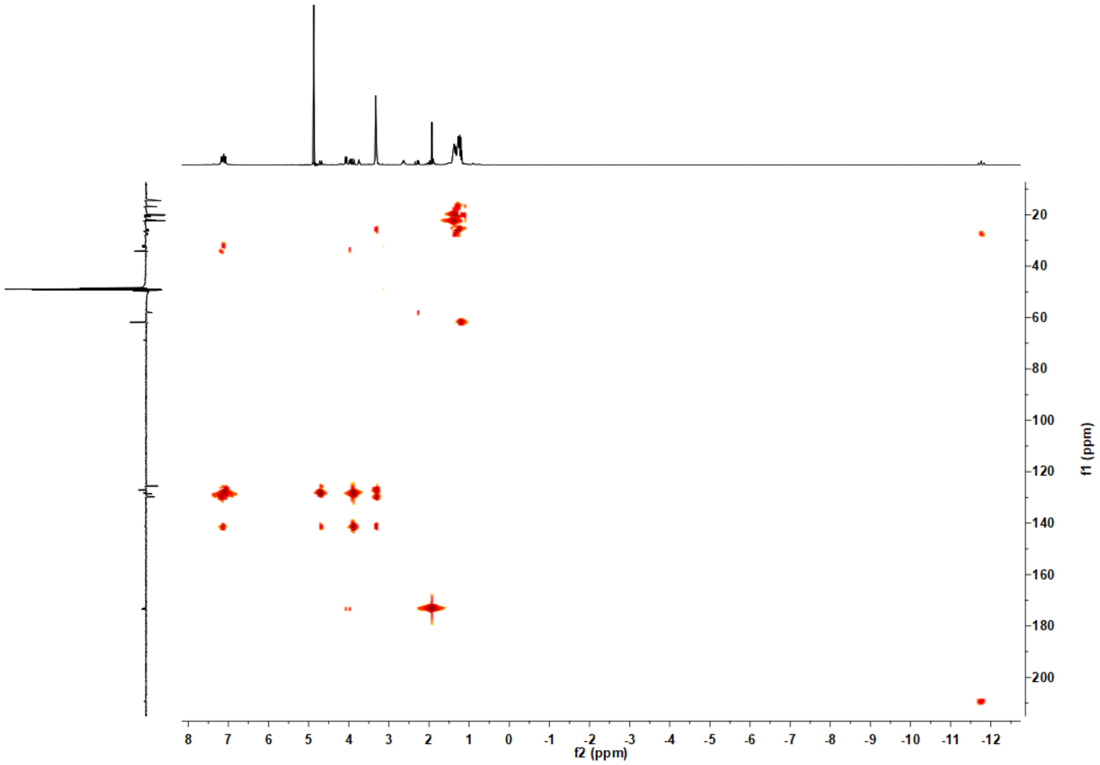

Figure S17. HMBC spectrum of **Ru-4** in CD_3OD.

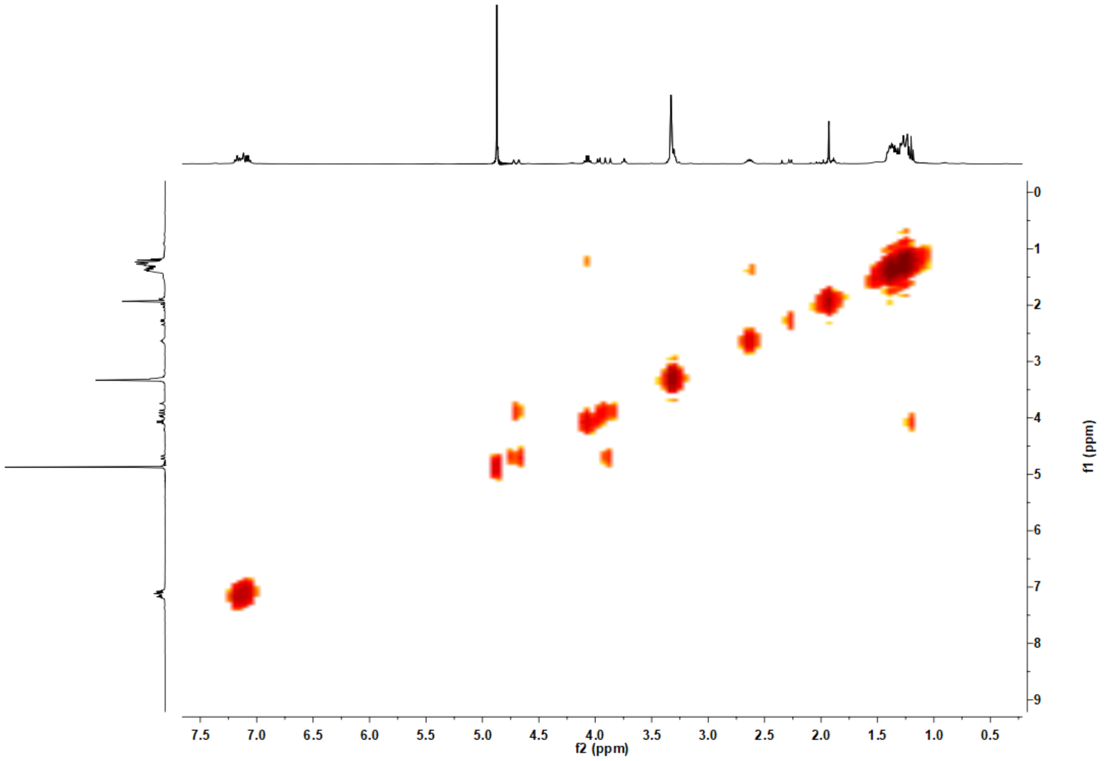

Figure S18. COSY spectrum of **Ru-4** in CD_3OD.

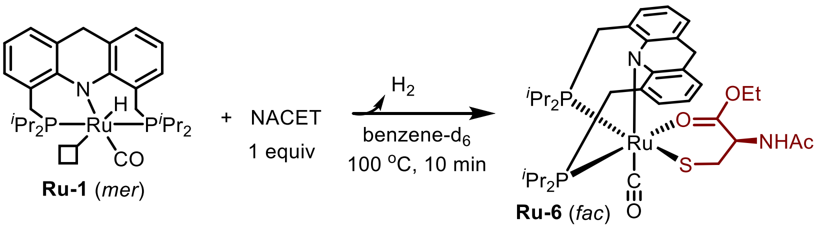

To a J. Young NMR tube was added complex **Ru-1** (11.4 mg, 0.02 mmol), NACET (3.8 mg, 0.02 mmol) and benzene-d_6 (0.5 mL) in a N_2 glovebox. The NMR tube was heated at 100 ^oC for 10 min with occasional release of the generated H_2. The color of the solution changed from orange to dark green upon heating, and finally turned to brown when the solution was cooled down to room temperature. NMR analysis indicated the generation of **Ru-6** as the major species, with a bidentate thiolate binding to the ruthenium center. The generation of **Ru-6** by heating **Ru-1** and NACET is not a clean reaction, with around 10% **Ru-4** and some unidentified species from the thermal decomposition of **Ru-6** in the solution. Due to the existence of chiral centers in the complex, multiple couplings were observed.

During the heating the color of the solution was dark green, which is in accordance to the color of a ruthenium thiolate complex (monodentate **Ru-5**).[2] The color of the solution quickly converted into brown upon cooling, implying the coordination of an additional group to the ruthenium center. Similar color change was also observed employing butyl 3-mercaptopropionate as the additive.

Ru-6: IR (cm^{-1}):2962, 1920, 1736, 1653, 1540, 1022. ^{1}H NMR (400 MHz, C_6D_6) δ 7.26 (d, J = 6.7 Hz, 1H, ArH), 7.11 – 6.93 (m, 4H, ArH), 6.91 – 6.84 (m, 1H, ArH), 6.39 (d, J = 6.9 Hz, 1H, N*H*Ac), 4.97 – 4.85 (m, 1H, C*H*NHAc), 4.47 (d, J = 15.6 Hz, 1H, ArCH_2Ar), 3.84 (d, J = 15.5 Hz, 1H, ArCH_2Ar), 3.60 (dd, J = 14.0, 6.9 Hz, 2H, COOCH_2CH$_3$), 3.43 (dd, J = 13.2, 4.8 Hz, 1H, SCH_2), 3.02 – 2.83 (m, 3H, PCH_2, PCH(CH$_3$)$_2$), 2.70 – 2.56 (m, 1H, SCH_2), 2.40 (ddd, J = 18.2, 12.2, 7.0 Hz, 4H, PCH_2), 2.24 – 2.04 (m, 3H, PCH(CH$_3$)$_2$), 1.51 (dd, J = 14.9, 7.3 Hz, 3H, PCH(CH_3)$_2$), 1.36 – 1.25 (m, 6H, PCH(CH_3)$_2$), 1.23 – 0.97 (m, 18H, PCH(CH_3)$_2$, NHCOCH_3), 0.74 (t, J = 7.0 Hz, 3H, COOCH$_2$CH_3). ^{13}C NMR (101 MHz, C_6D_6) δ206.54 (dd, J = 17.4, 12.1

Hz, Ru-CO), 172.82 (s, *C*OOCH$_2$CH$_3$), 172.58 (s, NHCO), 155.64 – 153.35 (m, ArC), 131.02 – 130.81 (m, ArC), 127.69 (s, ArC), 127.16 (d, *J* = 4.9 Hz, ArC), 126.23 (s, ArC), 125.53 (d, *J* = 1.2 Hz, ArC), 124.14 (d, *J* = 4.4 Hz, ArC), (d, *J* = 4.7 Hz, ArC), 118.37 (s, ArC),, 117.64 (s, ArC), 61.78 (s, COO*C*H$_2$CH$_3$), 56.98 (s, *C*HNHAc), 36.98 (s, S*C*H$_2$), 35.60 (s, Ar*C*H$_2$Ar), 33.77 – 33.06 (m, P*C*H(CH$_3$)$_2$), 29.34 (d, *J* = 23.2 Hz, P*C*H$_2$), 28.63 (d, *J* = 17.6 Hz, P*C*H$_2$), 28.23 (d, *J* = 31.3 Hz, P*C*H(CH$_3$)$_2$), 24.13 (d, *J* = 20.6 Hz, P*C*H(CH$_3$)$_2$), 22.59 (s, PCH(*C*H$_3$)$_2$), 21.13 (d, *J* = 2.6 Hz, PCH(*C*H$_3$)$_2$), 20.87(s, PCH(*C*H$_3$)$_2$), 20.08 – 19.52 (m, PCH(*C*H$_3$)$_2$, NHCO*C*H$_3$), 19.32 (d, *J* = 3.6 Hz, PCH(*C*H$_3$)$_2$), 13.87 (s, COOCH$_2$*C*H$_3$). ^{31}P NMR (162 MHz, C$_6$D$_6$) δ 85.72 (d, *J* = 8.8 Hz), 75.46 (d, *J* = 7.6 Hz).

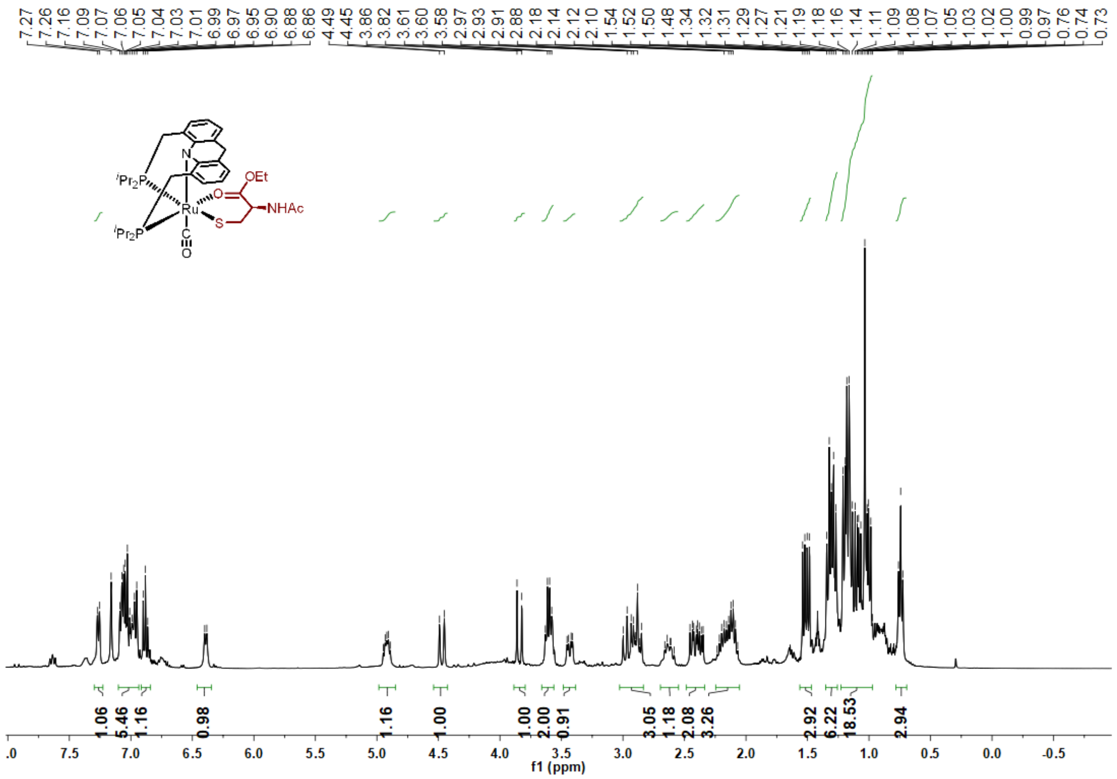

Figure S19. ^{1}H NMR spectrum of **Ru-6** in C$_6$D$_6$.

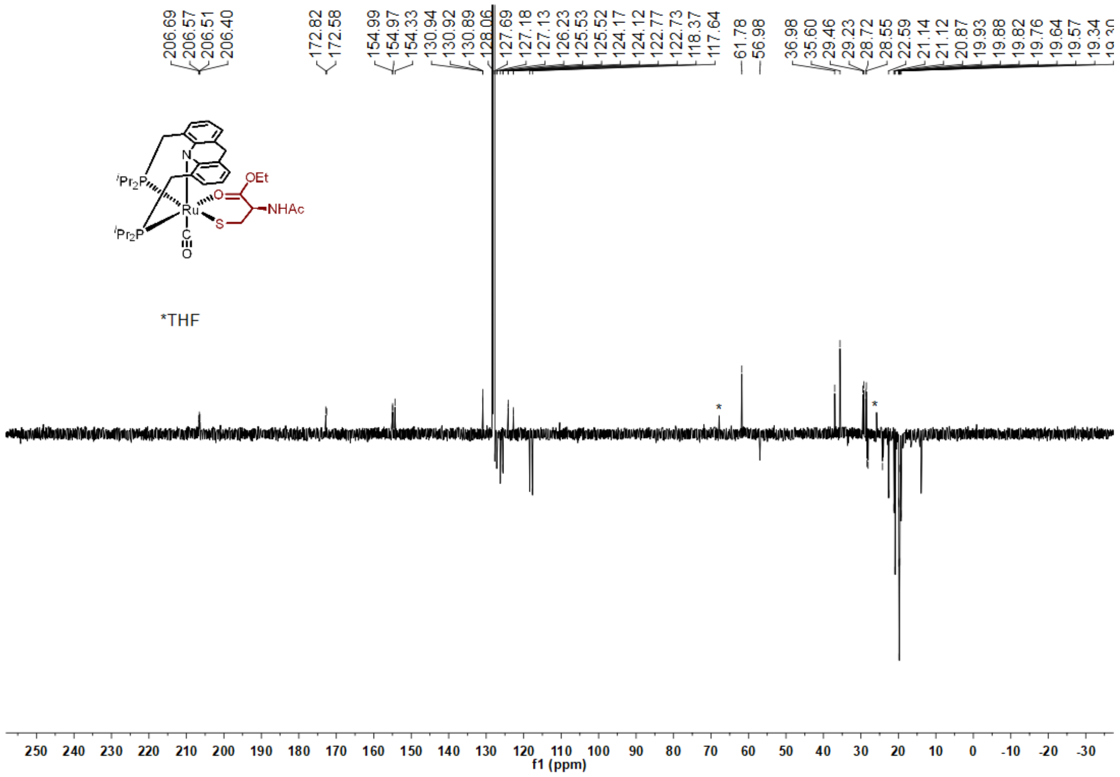

Figure S20. ^{13}C-DEPTQ NMR spectrum of **Ru-6** in C_6D_6.

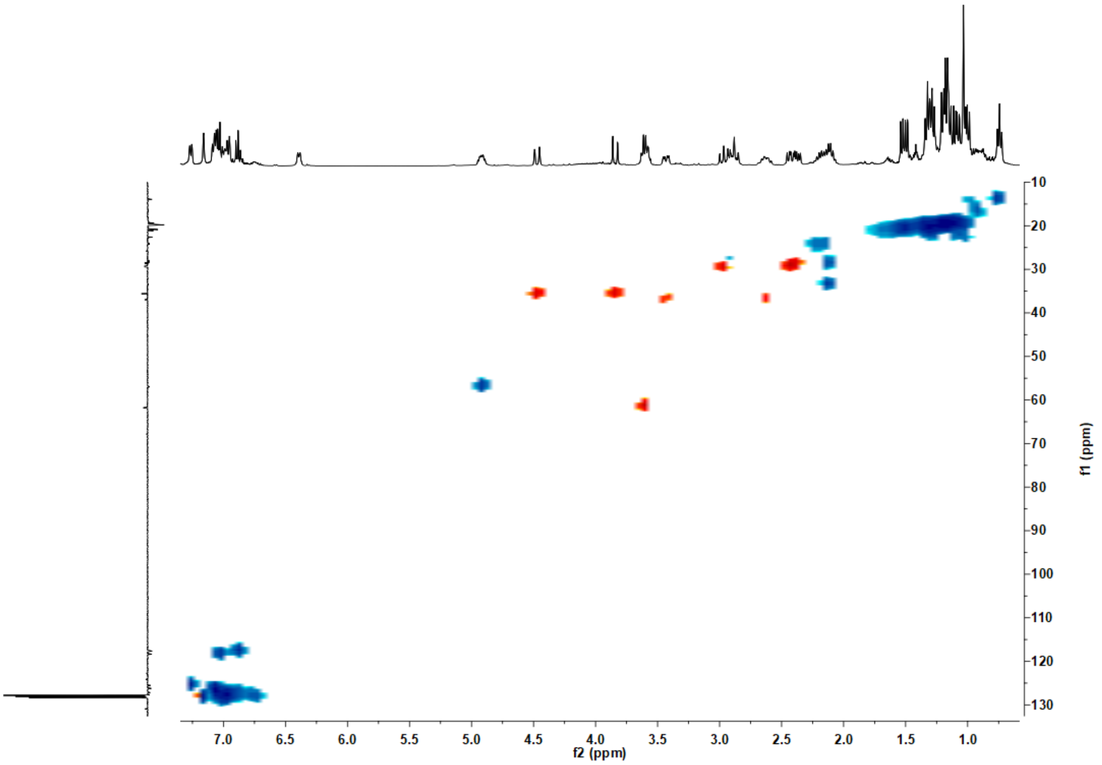

Figure S21. HSQC spectrum of **Ru-6** in C_6D_6.

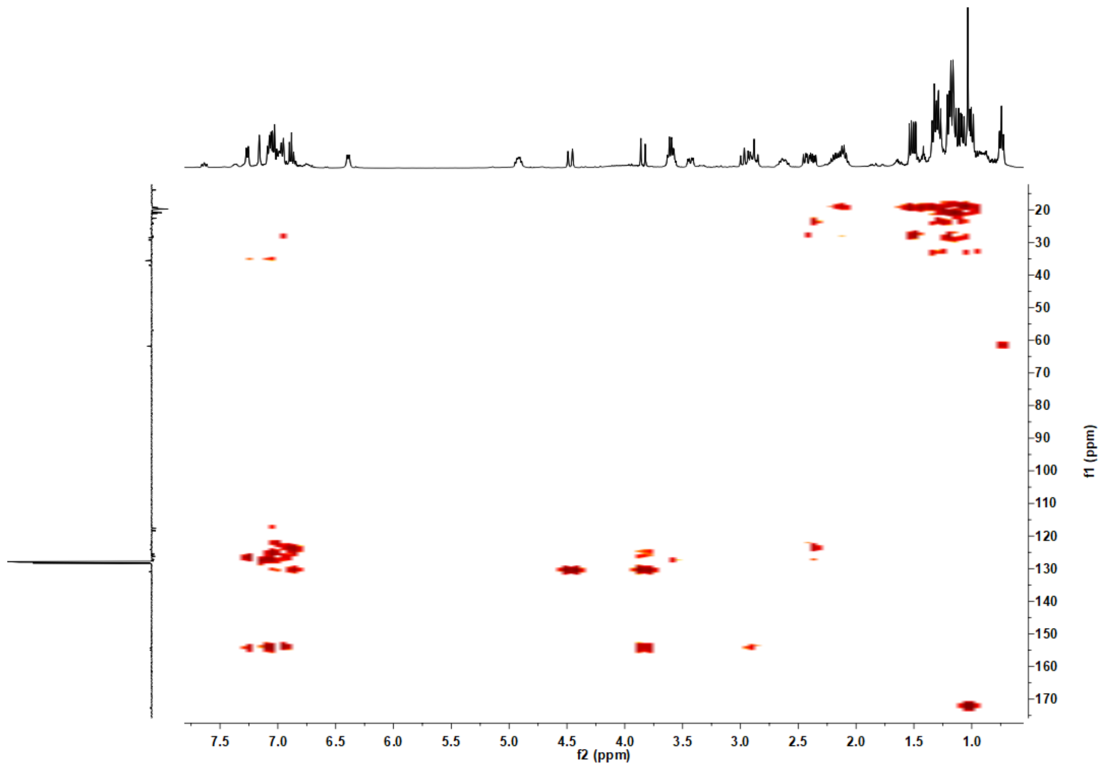

Figure S22. HMBC spectrum of **Ru-6** in C_6D_6.

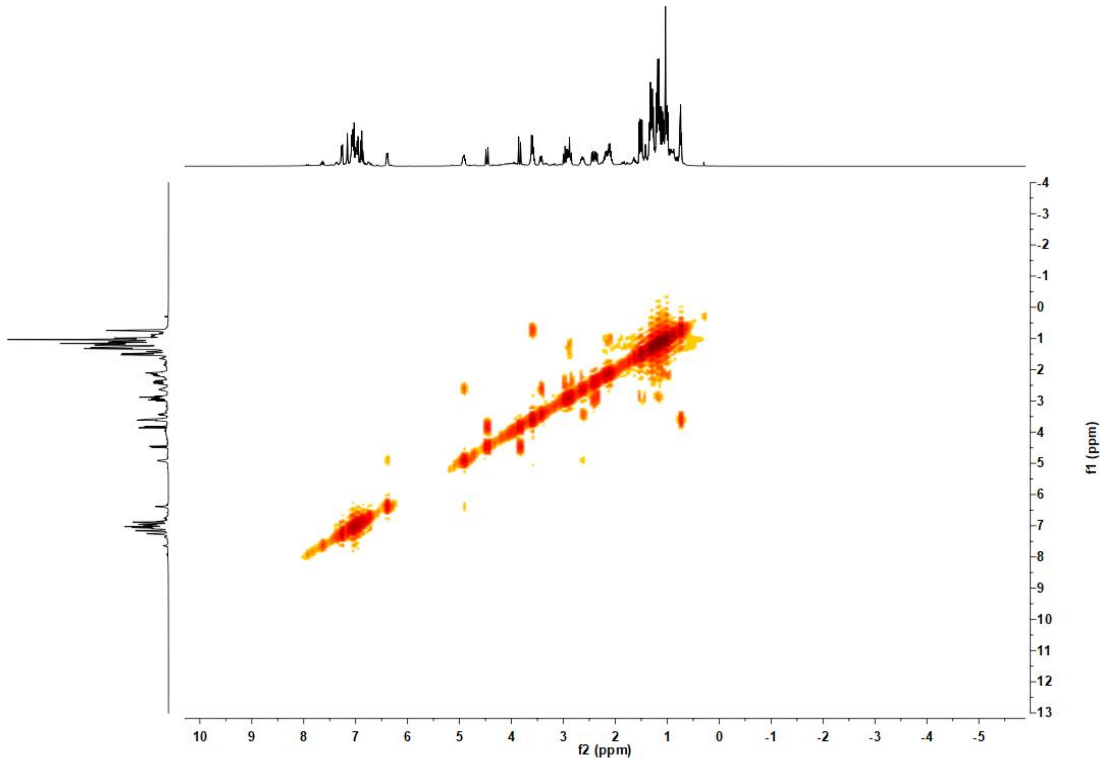

Figure S23. COSY spectrum of **Ru-6** in C_6D_6.

Ru-6' (with butyl 3-MPA): IR (cm^{-1}): 2959, 1929, 1731, 1671, 1435. ^{1}H NMR (500 MHz, C_6D_6) δ 7.19 – 7.14 (m, 1H, ArH), 7.06 – 7.02 (m, 1H, ArH), 7.01 – 6.97 (m, 2H, ArH), 6.95 – 6.89 (m, 2H, ArH), 4.39 (d, *J* = 15.7 Hz, 1H, ArCH_2Ar), 3.85 (d, *J* = 15.8 Hz, 1H, ArCH_2Ar), 2.91 (t, *J* = 13.1 Hz, 2H, SCH_2), 2.74 (br, 2H, COOCH_2), 2.52 – 2.38 (m, 4H, COOCH$_2$CH_2, CH_2COO), 2.38 – 2.28 (m, 2H, PC*H*(CH$_3$)$_2$), 2.14 – 2.05 (m, 2H, PC*H*(CH$_3$)$_2$), 1.39 (dd, *J* = 14.5, 7.3 Hz, 6H, PCH(CH_3)$_2$), 1.29 – 1.11 (m, 14H, PCH(CH_3)$_2$, PCH(CH_3)$_2$, COOCH$_2$CH$_2$CH_2), 1.06 (dd, *J* = 11.8, 7.3 Hz, 6H, PCH(CH_3)$_2$), 0.77 (t, *J* = 7.3 Hz, 3H, COOCH$_2$CH$_2$CH$_2$CH_3). ^{13}C NMR (126 MHz, C_6D_6) δ 205.70 (t, *J* = 14.2 Hz, Ru-CO), 154.89 (s, ArC), 134.30 (s, ArC), 134.15 (s, ArC), 130.81 (s, ArC), 128.89 (s, ArC), 128.78 (d, *J* = 6.9 Hz, ArC), 127.47 (d, *J* = 3.9 Hz), 125.98 (s, ArC), 122.93 (d, *J* = 3.1 Hz, ArC), 118.03 (s, ArC), 67.83 (s, COO*C*H$_2$), 65.45 – 64.59 (m, *C*OOCH$_2$), 35.63(s, Ar*C*H$_2$Ar), 31.18 (d, *J* = 17.2 Hz, P*C*H(CH$_3$)$_2$), 30.83(s, COOCH$_2$CH$_2$*C*H$_2$), 28.93 (s, SCH$_2$), 28.77 (s, *C*H$_2$COO), 26.23 (d, *J* = 26.1 Hz, P*C*H(CH$_3$)$_2$), 20.98 (s, PCH(*C*H$_3$)$_2$), 19.76 (s, PCH(*C*H$_3$)$_2$), 19.61 (dd, *J* = 8.4, 4.5 Hz, PCH(*C*H$_3$)$_2$), 19.28 (s, COOCH$_2$*C*H$_2$CH$_2$), 13.89 (s, COOCH$_2$CH$_2$CH$_2$*C*H$_3$). ^{31}P NMR (202 MHz, C_6D_6) δ 81.59.

3. Control experiments

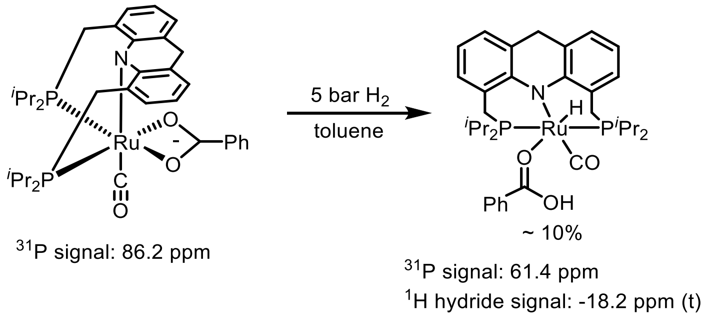

To a J. Young NMR tube was added the carboxylate complex[6] (3.5 mg, 0.005 mmol) and toluene (0.5 mL) in a N_2 glovebox. The NMR tube was taken out of the box and pressurized with 5 bar of H_2, and allowed to rotate at room temperature for 5 min. ^{31}P NMR measurement of the sample indicate the generation of a new species, which was attributed to a hydride species (Ru-H observed in ^{1}H NMR), but in only ~10% according to phosphorus integrals in ^{31}PNMR. The results indicate the activation of H_2 by the ruthenium carboxylate complex is difficult. Note: **Ru-5** or **Ru-6** can activate H_2 and transform into **Ru-4** immediately under 5 bar of H_2.

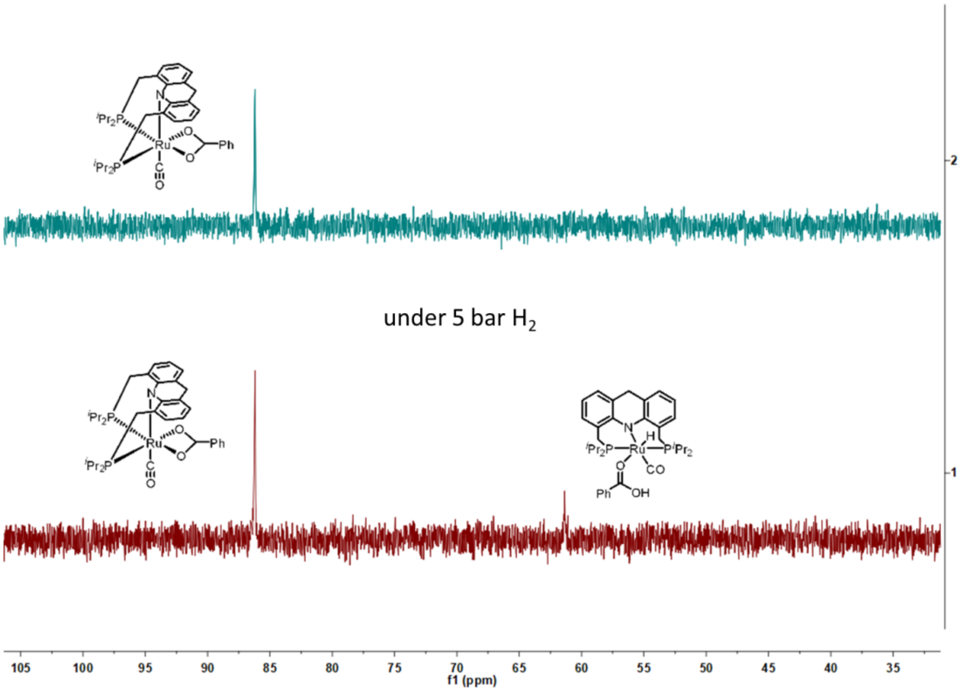

Figure S24. Sluggish activation of H_2 by the ruthenium carboxylate complex (^{31}P NMR)

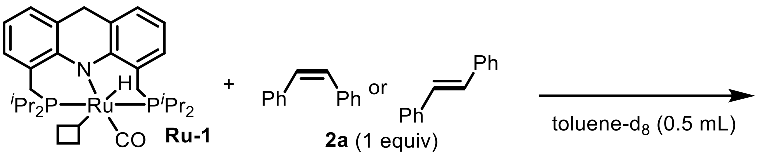

To a J. Young NMR tube was added **Ru-1** (11.4 mg, 0.02 mmol), **2a** (3.6 mg, 0.02 mmol) and toluene-d$_8$ (0.5 mL) in a N_2 glovebox. The NMR tube was taken out of the box and allowed to rotate at room temperature for 1 min. ^{31}P NMR and ^{1}H NMR measurements of the sample indicated no shift of the signals of **Ru-1** but with little broadening, especially of the hydride signal and the protons of phosphorus substituents.

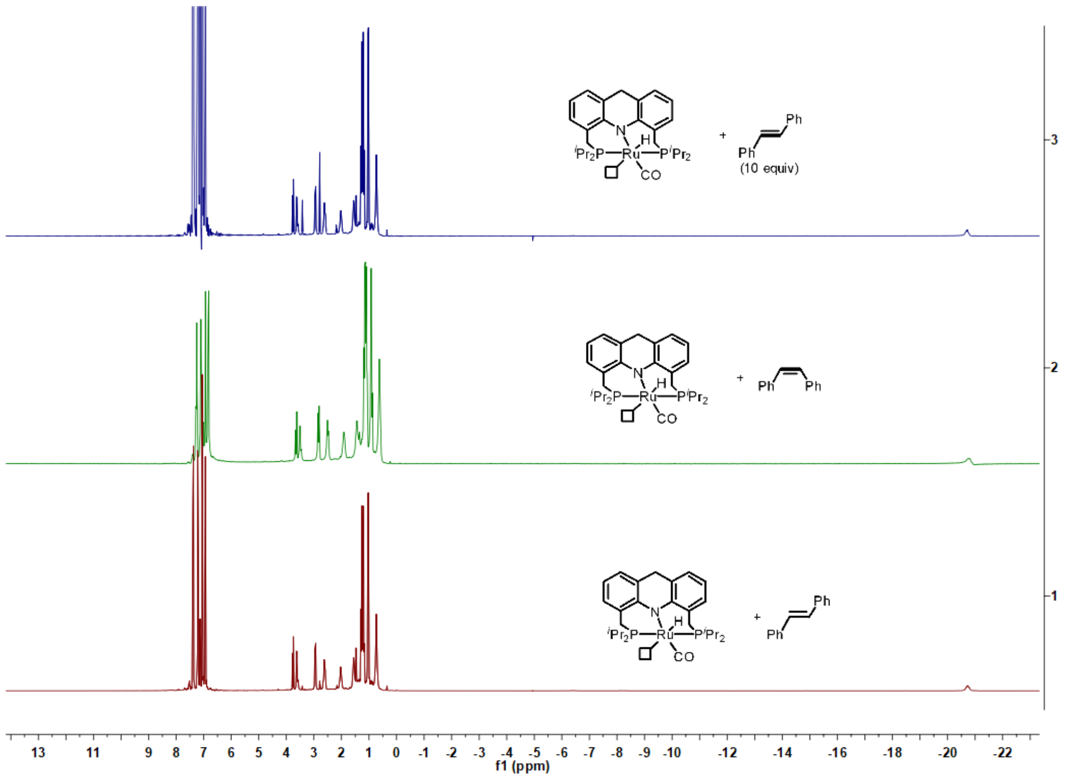

Figure S25. Weak interaction between **Ru-1** and alkenes (^{1}H NMR)

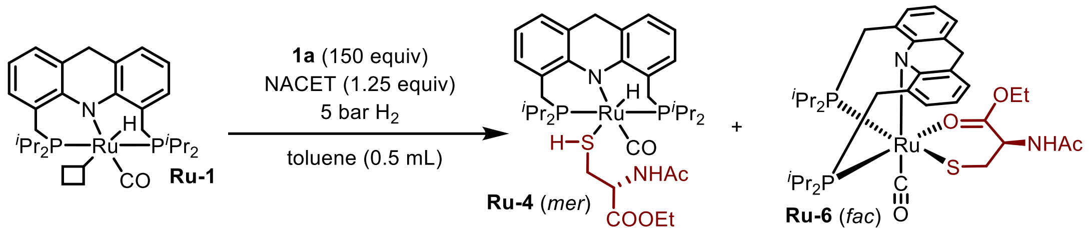

To a J. Young NMR tube was added **Ru-1** (2.8 mg, 0.005 mmol), NACET (1.2 mg, 1.25 equiv), **1a** (0.13 g, 150 equiv) and toluene (0.5 mL) in a N_2 glovebox. The NMR tube was taken out of the box and pressurized with 5 bar of H_2 and allowed to rotate at room temperature for 30 min. ^{31}P NMR measurement (NS>600) of the sample indicated the generation of **Ru-4** as the major species, with little signals of **Ru-6**.

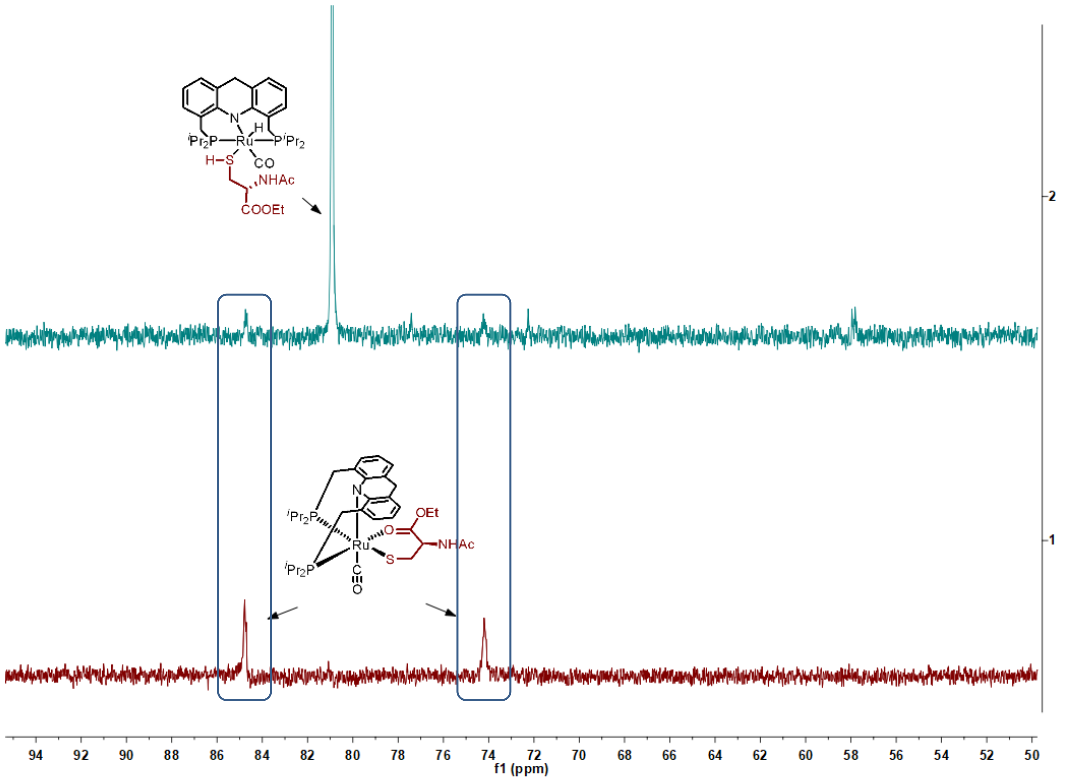

Figure S26. Monitoring the ruthenium species in actual catalysis with NACET (^{31}P NMR)

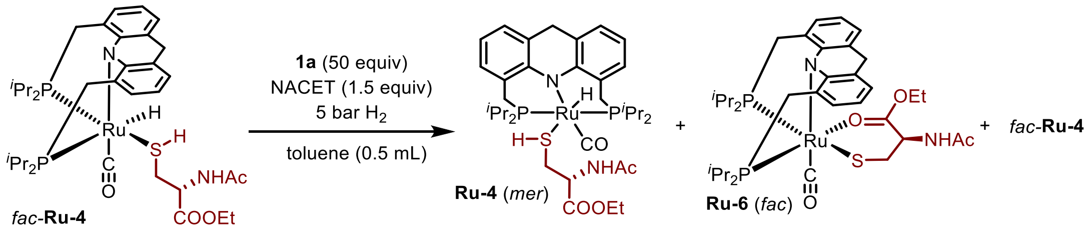

To a J. Young NMR tube was added **Ru-1** (2.8 mg, 0.005 mmol), NACET (2.4 mg, 2.5 equiv), and toluene (0.5 mL) in a N_2 glovebox. The NMR tube was taken out of the box and heated at 100 °C for 5 min, generating **Ru-6**. The tube was pressurized with 5 bar of H_2, and *fac*-**Ru-4** was immediately generated (Figure S27).

The tube was transferred back to the glovebox, after release of H_2 gas in glovebox, **1a** (44.5 mg 50 equiv) was quickly added and the tube was taken out of the box and pressurized with 5 bar of H_2. Immediate ^{31}P NMR measurement of the sample indicated the generation of **Ru-6**, **Ru-4** (*mer*) with some *fac*-**Ru-4** still in the solution. ^{1}H NMR also indicated the generation of (Z)-**2a** at this point. No species other than **Ru-4** was observed when alkene (Z)-**2a** was added following similar procedures, indicating poor affinity of the alkene toward the catalyst.

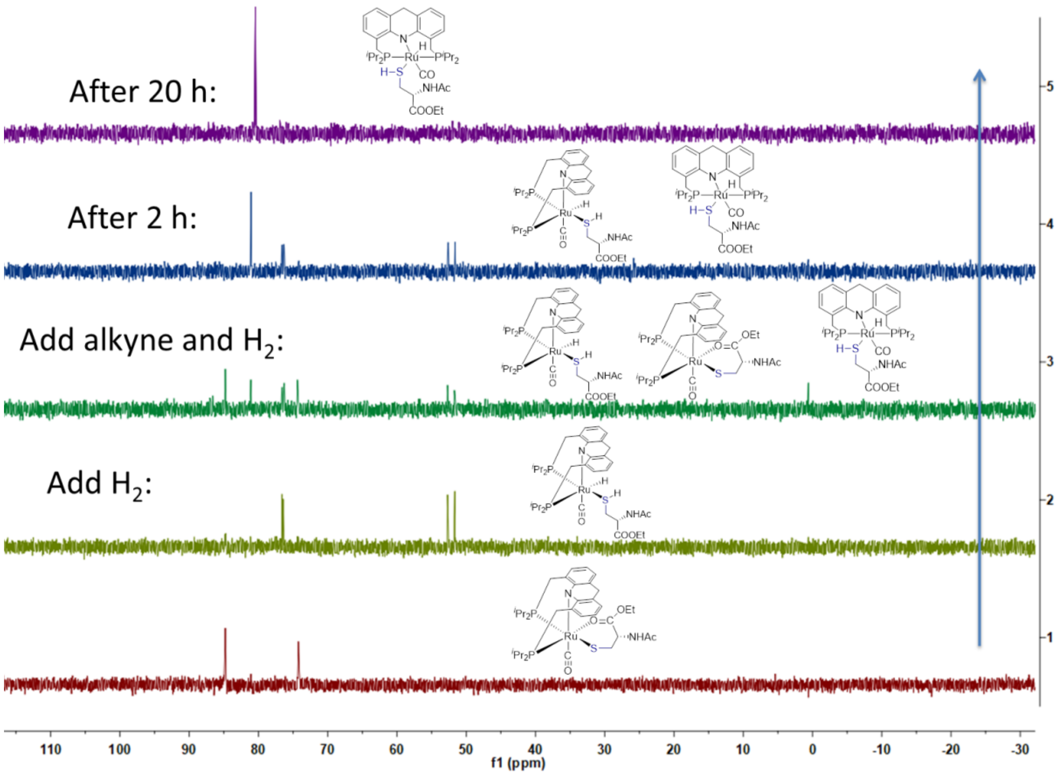

Figure S27. Monitoring the ruthenium species in catalysis with **Ru-6** as the catalyst (^{31}P NMR)

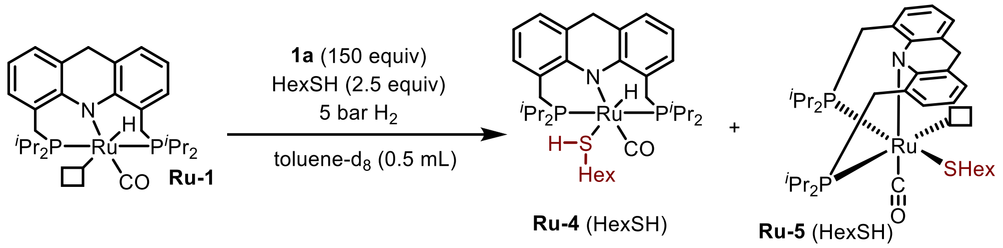

To a J. Young NMR tube was added **Ru-1** (2.8 mg, 0.005 mmol), hexanethiol (1.8 μL, 2.5 equiv), **1a** (44.5 mg, 50 equiv) and toluene-d$_8$ (0.5 mL) in a N_2 glovebox. The NMR tube was taken out of the box and measured by NMR, indicating the generation of **Ru-4** (HexSH) and **Ru-5** (HexSH). The tube was pressurized with 5 bar of H_2 and allowed to rotate once. Immediate color change from green to red brown was observed. After standing for 5 min, the red brown color changed back to light green gradually, with the top solution still keeping red brown (Figure S28). ^{31}P NMR measurement of the sample only detected **Ru-4** (HexSH) and **Ru-5** (HexSH) during the reaction (Figure S29). However, from the ^{1}H NMR we could observe the vinylic proton of **Ru-2**, although in a small amount (Figure S30). In addition, the red brown colour observed during the reaction, which is characteristic of **Ru-2**, also supports that the alkenyl species appeared during the reaction. These results imply an inner-sphere hydrogenation of alkynes in the presence of thiol.

Note: As mentioned above, **Ru-2** has a very broad ^{31}P signal, thus it's difficult to detect, especially in a small amount, by ^{31}P NMR.

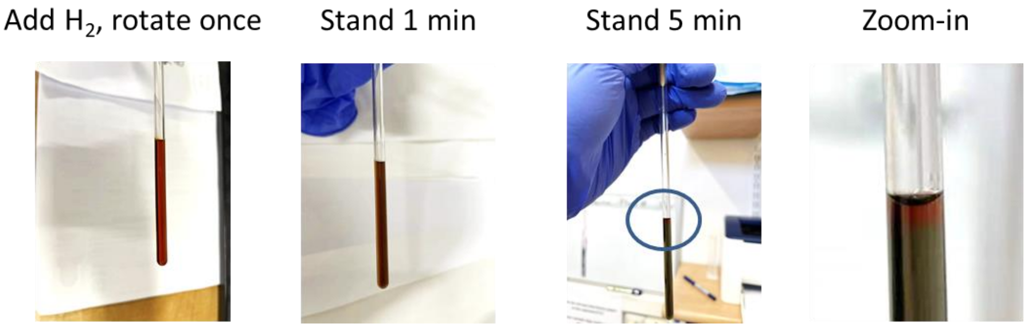

Figure S28. Evident color change employing HexSH as the additive.

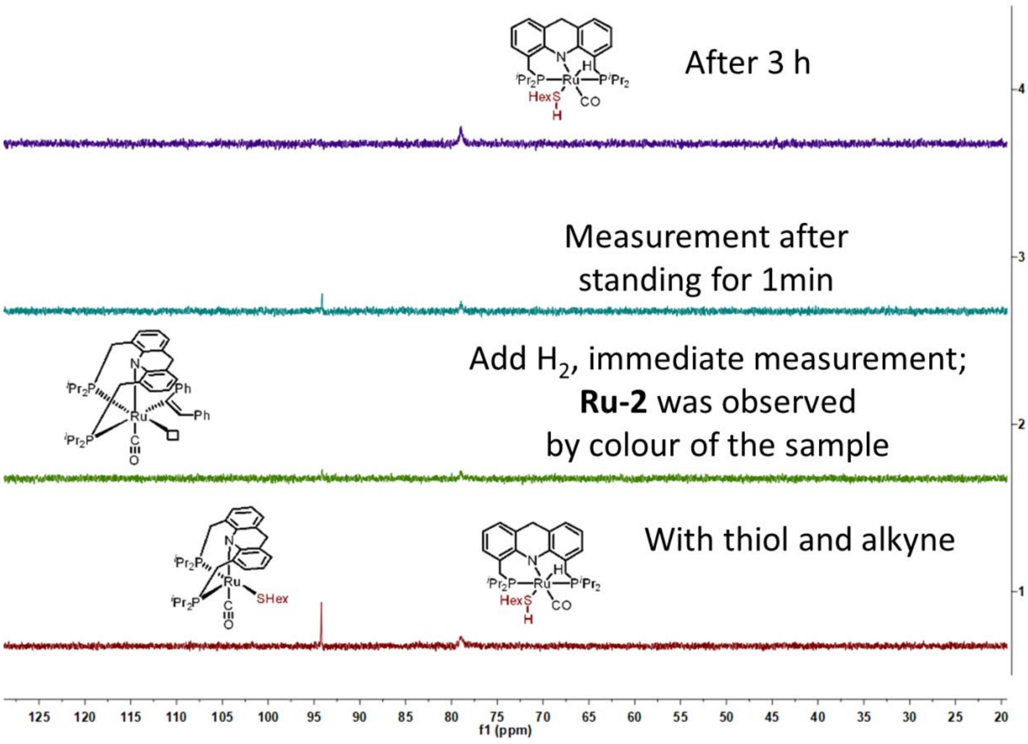

Figure S29. Monitoring the ruthenium species in actual catalysis with HexSH (^{31}P NMR, same number of scans for all spectra)

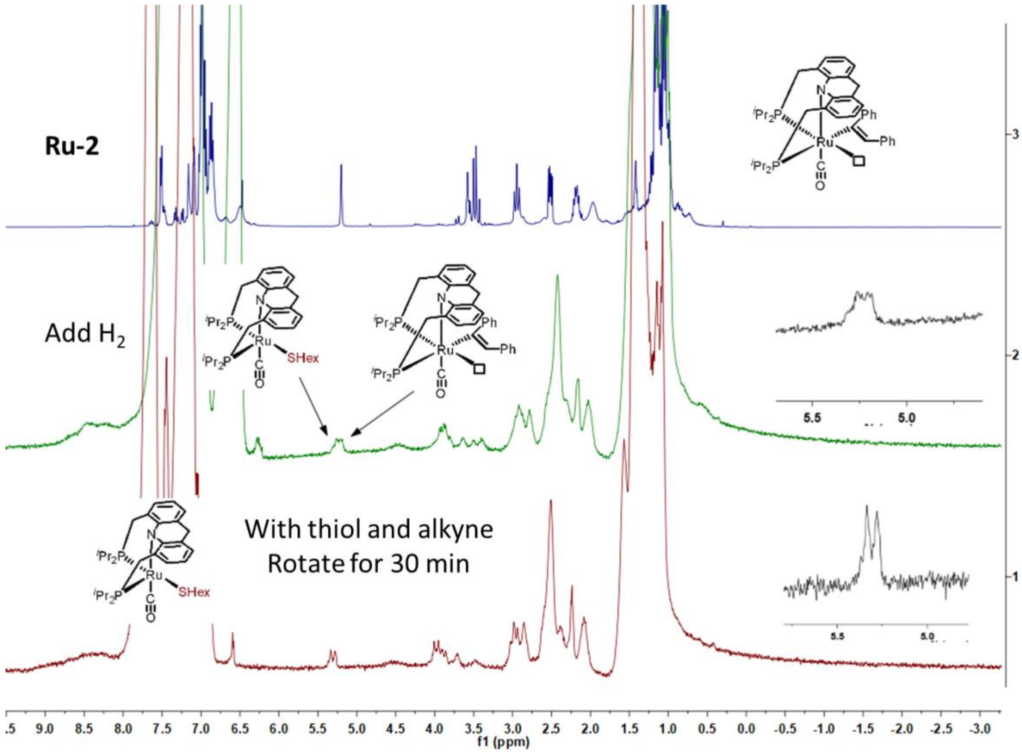

Figure S30. Monitoring the ruthenium species in actual catalysis with HexSH (^{1}H NMR)

4. General experimental procedures

Typical procedure for controllable Z/E semihydrogenation of alkynes.

NMR experiments (0.25 mmol):

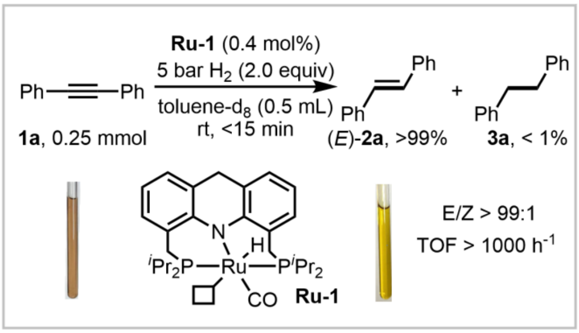

Trans-semihydrogenation of alkynes: In a N_2-filled glovebox, a 3 mL high pressure J-Young NMR tube was charged with **1a** (44.5 mg, 0.25 mmol), internal standard 1,3,5-trimethoxybenzene (42.0 mg, 0.25 mmol), **Ru-1** (1.0 μmol, 0.6 mg in 0.4 mL toluene-d_8), toluene-d_8 (0.1 mL). The tube was taken out of the glovebox, rotated for 10 min, and pressurized with 5 bar of H_2, and was rotated at room temperature. An evident colour change to yellow was observed within 15 min. Then the tube was analyzed by NMR, indicating the full conversion of **1a** and the generation of (E)-**2a**.

Note: It was found a little higher TOF (>1150 h^{-1}) could be achieved using a bigger tube with 10 mL volume. In addition, the rotation of the NMR tube is essential to the conversion of the NMR experiments of the hydrogenation reactions, possibly due to the solvation rate of H_2 into the solution. Thus, the abscissa of the kinetic profiles of the reaction only counts the rotation time (except Figure 1f). In addition, a little longer reaction period was required for the monitoring than the actual catalysis, possibly because of the disruption of the rotation of the NMR tube (especially the step of isomerization, see Note S1). NMR experiments in Figure 1f (independent Z-E isomerization tests) were carried out without the rotation of the tube, and measured by *in-situ* NMR.

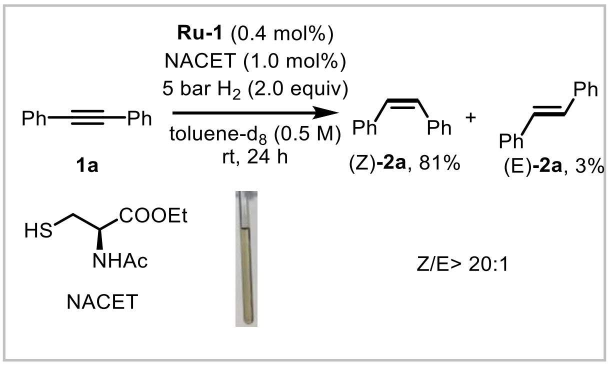

Cis-semihydrogenation of alkynes: In a N_2-filled glovebox, a 3 mL high pressure J-Young NMR tube was charged with **1a** (44.5 mg, 0.25 mmol), internal standard 1,3,5-trimethoxybenzene (42.0 mg, 0.25 mmol), NACET (2.5 μmol, 0.5 mg in 0.1 mL toluene-d_8), and **Ru-1** (1.0 μmol, 0.6 mg in 0.4 mL toluene-d_8). The tube was taken out of the glovebox, rotated for 10 min, and pressurized with 5 bar of H_2, and was rotated at room temperature. The colour of the solution kept unchanged for the whole reaction period (light green). After 24 h, the tube was analyzed by NMR, indicating 86% conversion of **1a** and the generation of 81% (Z)-**2a** and 3% (E)-**2a**.

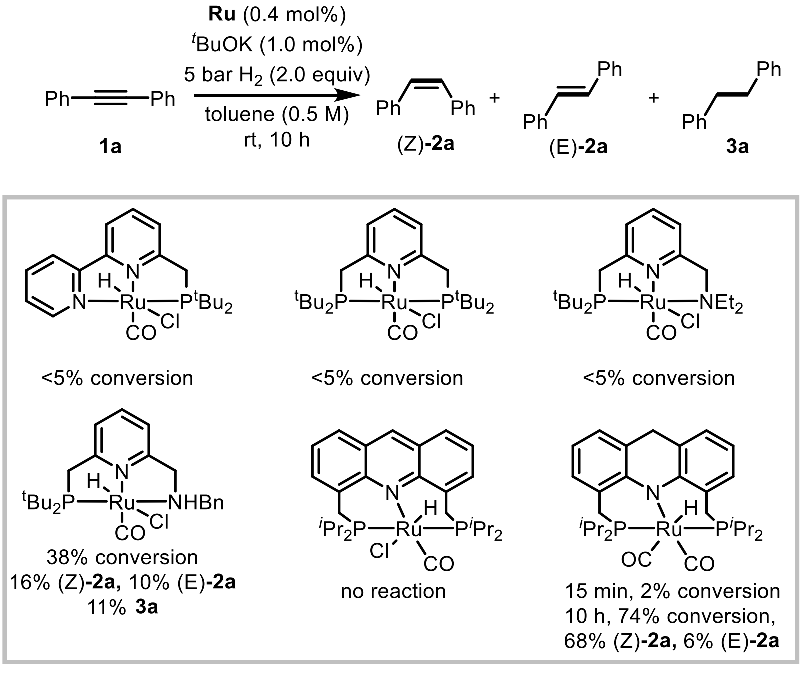

Figure S31. Catalysts test.

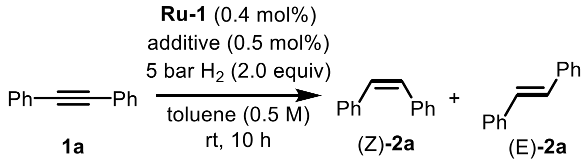

MeOH	MeOH (solvent)	PhCOOH	HexSH	PhSPh
Full conversion<br>(Z)-**2a**, n.d.<br>(E)-**2a**, >99%	Full conversion<br>(Z)-**2a**, n.d.<br>(E)-**2a**, >99%	<5% conversion<br>(Z)-**2a**, <3%<br>(E)-**2a**, <3%	Full conversion<br>(Z)-**2a**, n.d.<br>(E)-**2a**, >99%	Full conversion<br>(Z)-**2a**, n.d.<br>(E)-**2a**, >99%
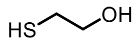 BME	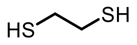 EDT	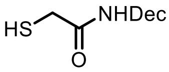 2-MAA	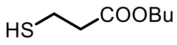 3-MPA	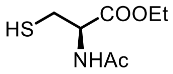 NACET
Full conversion<br>(Z)-**2a**, 41%<br>(E)-**2a**, 59%	No reaction<br>(Z)-**2a**, n.d.<br>(E)-**2a**, n.d.	No reaction<br>(Z)-**2a**, n.d.<br>(E)-**2a**, n.d.	Full conversion<br>(Z)-**2a**, 49%<br>(E)-**2a**, 50%	96% conversion<br>(Z)-**2a**, 87%<br>(E)-**2a**, 10%
$HexNH_2$	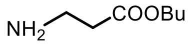	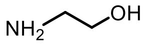	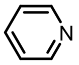	PPh_3
Full conversion<br>(Z)-**2a**, 4%<br>(E)-**2a**, 96%	Full conversion<br>(Z)-**2a**, n.d.<br>(E)-**2a**, >99%	Full conversion<br>(Z)-**2a**, n.d.<br>(E)-**2a**, >99%	Full conversion<br>(Z)-**2a**, n.d.<br>(E)-**2a**, >99%	Full conversion<br>(Z)-**2a**, n.d.<br>(E)-**2a**, >99%

Figure S32. Additives test.

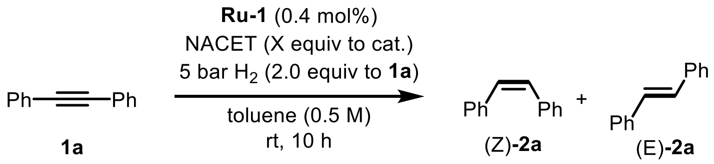

0.5 equiv	1.25 equiv	2.5 equiv	5 equiv	2.5 equiv (24 h)	2.5 equiv (48 h)
Full conversion<br>(Z)-**2a**, n.d.<br>(E)-**2a**, >99%	96% conversion<br>(Z)-**2a**, 87%<br>(E)-**2a**, 10%	38% conversion<br>(Z)-**2a**, 38%<br>(E)-**2a**, n.d.	24% conversion<br>(Z)-**2a**, 24%<br>(E)-**2a**, n.d.	83% conversion<br>(Z)-**2a**, 81%<br>(E)-**2a**, 3%	87% conversion<br>(Z)-**2a**, 89%<br>(E)-**2a**, 7%

Figure S33. NACET amounts test.

0.5 mmol scale experiments:

Trans-semihydrogenation of alkynes: In a N_2-filled glovebox, a 90 mL Fischer-Porter tube was charged with internal standard 1,3,5-trimethoxybenzene (42.0 mg, 0.25 mmol), alkyne (0.5 mmol), **Ru-1** (1.0 μmol, 0.6 mg in 0.8 mL toluene), toluene (0.2 mL) and a stirring bar. The tube was taken out of the glovebox and pressurized with 1 bar of H_2, and resulting mixture was stirred at rt. The reaction proceeded while the color of the solution reached yellow as an indicator. Then the

tube's valve was slowly opened to release the hydrogen gas. The resulting solution was analyzed by GC-MS and NMR.

Cis-semihydrogenation of alkynes: In a N_2-filled glovebox, a 90 mL Fischer-Porter tube was charged with internal standard 1,3,5-trimethoxybenzene (42.0 mg, 0.25 mmol), alkyne (0.5 mmol), **Ru-1** (1.0 μmol, 0.6 mg in 0.8 mL toluene), NACET (2.5 μmol, 0.5 mg in 0.2 mL toluene) and a stirring bar. The tube was taken out of the glovebox and pressurized with 1 bar of H_2, and the resulting mixture was stirred at rt. The reaction proceeded, and samples were withdrawn at regular intervals, and analyzed by GC-MS and NMR.

Table S1 Variations of reaction conditions of each substrate

(*E*)-Products	variations	(*Z*)-Products	variations
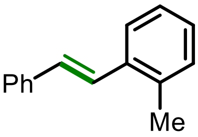<br>**2b**, 99%<br>E/Z = 49/1	2 bar of H_2<br>40 ^oC, 7 d	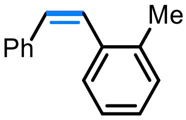<br>**2b**, 95%<br>Z/E = 47/1	7 d
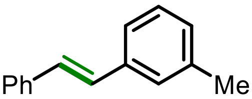<br>**2c**, 99%<br>E/Z = >99/1	15 h	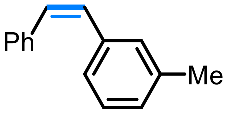<br>**2c**, 92%<br>Z/E = 13/1	18 h
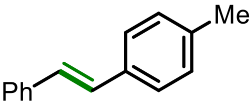<br>**2d**, 99%<br>E/Z = >99/1	14 h	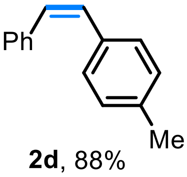<br>**2d**, 88%<br>Z/E = 21/1	48 h
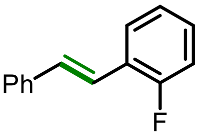<br>**2e**, 99%<br>E/Z = 81/1	14 h	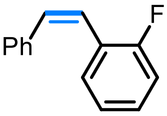<br>**2e**, 90%<br>Z/E = 17/1	92 h

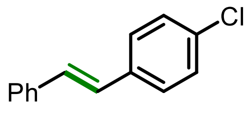<br>**2f**, 99%<br>E/Z = >99/1	24 h	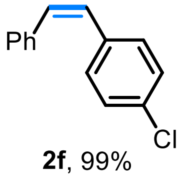<br>**2f**, 99%<br>Z/E = 12/1	18 h
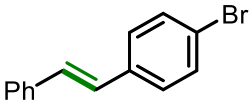<br>**2g**, 99%<br>E/Z = >99/1	15 h<br>In 1 mL THF	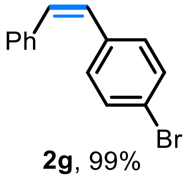<br>**2g**, 99%<br>Z/E = 16/1	12 h<br>In 1 mL THF
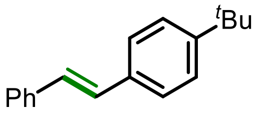<br>**2h**, 99%<br>E/Z = >99/1	40 h	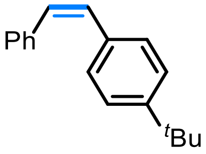<br>**2h**, 90%<br>Z/E = 8/1	38 h
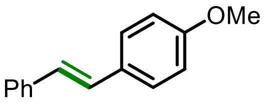<br>**2i**, 99%<br>E/Z = >99/1	15 h	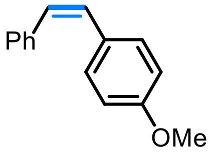<br>**2i**, 99%<br>Z/E = 49/1	18 h
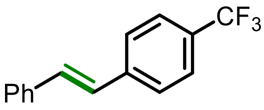<br>**2j**, 98%<br>E/Z = >99/1	20 h	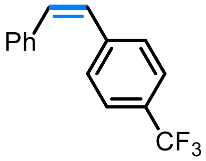<br>**2j**, 98%<br>Z/E = 15/1	17 h
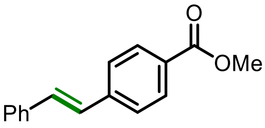<br>**2k**, 98%<br>E/Z = >99/1	18 h	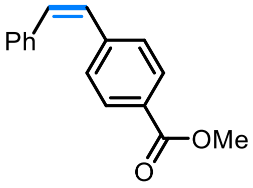<br>**2k**, 86%<br>Z/E = 41/1	14 h
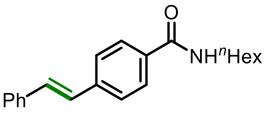<br>**2l**, 97%<br>E/Z = >99/1	21 h<br>In 2 mL THF<br>97% isolated yield	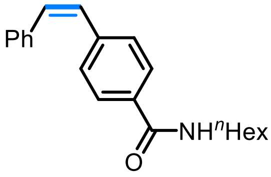<br>**2l**, 94%<br>Z/E = 20/1	26 h<br>In 2 mL THF;<br>99% isolated yield after 29 h, with Z/E = 11:1<br>(full conversion)

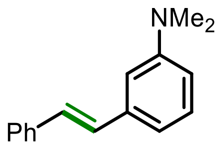 **2m**, 99%<br>E/Z = >99/1	16 h	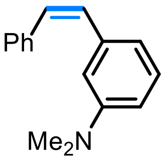 **2m**, 88%<br>Z/E = 12/1	18 h
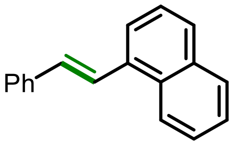 **2n**, 96%<br>E/Z = 96/1	2 bar of H_2<br>40 °C, 40 h<br>4%<br>over-reduction	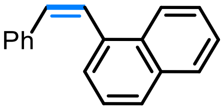 **2n**, 96%<br>Z/E = >99/1	2 bar of H_2<br>12 d
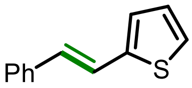 **2o**, 98%<br>E/Z = 36/1	7 d	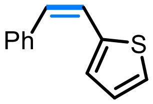 **2o**, 92%<br>Z/E = 22/1	2 bar of H_2<br>4 d
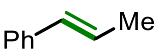 **2p**, 84%<br>E/Z=99/1	15 h<br>without stirring<br>14%<br>over-reduction	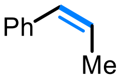 **2p**, 87%<br>Z/E=10/1	NACET<br>(1.0 mol%)<br>19 h<br>without stirring
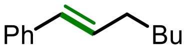 **2q**, 93%<br>E/Z = 92/1	14 h	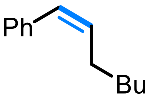 **2q**, 83%<br>Z/E = 16/1	NACET<br>(1.0 mol%)<br>30 h
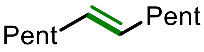 **2r**, 96%<br>E/Z=5/1	0 °C, 8 h	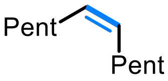 **2r**, 94%<br>Z/E=7/1	NACET<br>(1.0 mol%)<br>24 h
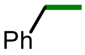 **3s**, 96%<br>**3s**/**2s** = > 99:1	2 bar of H_2, 15 h	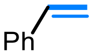 **2s**, 92%<br>**2s**/**3s** = 99:1	2 bar of H_2, 15 h

5 mmol scale experiments:

Trans-semihydrogenation of **1a**: In a N_2-filled glovebox, a 90 mL Fischer-Porter tube was charged with alkyne **1a** (0.89 g, 5 mmol), **Ru-1** (2.8 mg, 5.0 μmol), toluene (5 mL) and a stirring bar. The tube was taken out of the glovebox and pressurized with about 2.2 bar of H_2, and the resulting mixture was stirred at 25 °C. The color of the resulting solution changed from red brown to yellow in 15 h, with the hydrogen

pressure dropping to about 0.8 bar. Then the hydrogen gas was slowly released and the solvent was removed under vaccum. The resulting yellow solid was washed with MeOH, resulting in **2a** as a white solid in 96% isolated yield (E/Z>99:1 detected from crude mixture, yield of **3a** < 0.1%).

Cis-semihydrogenation of **1a**: In a N_2-filled glovebox, a 90 mL Fischer-Porter tube was charged with alkyne **1a** (0.89 g, 5 mmol), **Ru-1** (2.8 mg, 5.0 μmol), NACET (2.4 mg, 12.5 μmol), toluene (5 mL) and a stirring bar. The tube was taken out of the glovebox and pressurized with about 2.2 bar of H_2, and the resulting mixture was stirred at 25 ^oC. After 68 h, the hydrogen pressure dropped to 1 bar. Then the hydrogen gas was slowly released and the solvent was removed under vaccum. The crude mixture was directly analyzed by NMR, indicating Z/E selectivity of >20:1. Then the crude mixture was dissolved in hexane and passed through a silica column, after which the solvent was removed to afford a mixture of alkenes and the unreacted alkyne (84% NMR yield according to the weight and integrals).

20 mmol scale experiments:

Trans-semihydrogenation of **1a**: In a N_2-filled glovebox, a 90 mL Fischer-Porter tube was charged with alkyne **1a** (3.56 g, 20 mmol), **Ru-1** (11.4 mg, 20 μmol), toluene (10 mL) and a stirring bar. The tube was taken out of the glovebox and pressurized with 7 bar of H_2, and the resulting mixture was stirred at 25 ^oC. After 29 h, the solution solidified due to the accumulation of (E)-**2a**, with hydrogen pressure dropping to 1 bar. Then the Fischer-Porter tube was transferred back to the glovebox and hydrogen was slowly released. Additional 10 mL toluene was added to the tube and it was sealed and taken out of the box. The hydrogen pressure of the tube was resumed to 1.5 bar and the reaction was continued with stirring. After 15 h, the tube's valve was slowly opened to release the hydrogen gas and the crude mixture was analyzed by GC-MS, with nearly no other signals in addition to (E)-**2a** detected. The crude mixture was directly measured by NMR, indicating selectivity E/Z of >99:1. The solvent was removed under vaccum and the resulting yellow solid was washed with MeOH, resulting in (E)-**2a** as a white solid in 98% isolated yield. (Note: The

limited size (volume) of the reaction tube employed leads to this two-steps procedure for a scale-up reaction. Apparently the reaction procedure can be simplified with a larger reaction tube, see 5 mmol scale case above.)

Cis-semihydrogenation of **1a**: In a N_2-filled glovebox, a 90 mL Fischer-Porter tube was charged with alkyne **1a** (3.56 g, 20 mmol), **Ru-1** (11.4 mg, 20 μmol, NACET (9.6 mg, 50 μmol), toluene (10 mL) and a stirring bar. The tube was taken out of the glovebox and pressurized with 7 bar of H_2, and the resulting mixture was stirred at 25 ^oC. After 44 h, the hydrogen pressure dropped to ~1.8 bar. Then the tube's valve was slowly opened to release the hydrogen gas and the solvent was removed under vaccum. The crude mixture was directly measured by NMR, indicating selectivity Z/E of >20:1. Then the crude mixture was dissolved in hexane and passed through a silica column, after which the solvent was removed to afford a mixture of alkenes and the unreacted alkyne (85% NMR yield according to the weight and integrals).

Cautions:

(i) The Fischer-Porter tube and the high pressure J-Young NMR tube should be shielded with an iron net and kept behind a shielder.

(ii) Hydrogen is a flammable gas. Reactions associated with H_2 gas should be handled carefully inside a proper fume hoods without any flame, spark or static electricity sources nearby.

(iii) **Ru-1** and **Ru-4**-**Ru-6** are air sensitive, thus exposing the reaction to air can quench the reaction in a short period.

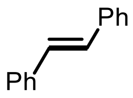

2a (20 mmol): 98% isolated yield, E/Z = >99:1. ^{1}H NMR (400 MHz, $CDCl_3$) δ 7.63 (d, J = 7.8 Hz, 4H), 7.47 (t, J = 7.5 Hz, 4H), 7.37 (t, J = 7.3 Hz, 2H), 7.23 (s, 2H). ^{13}C NMR (101 MHz, $CDCl_3$) δ 137.34, 130.35, 128.97, 128.31, 127.19.

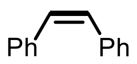

2a (20 mmol): 85% yield, Z/E = >20:1. ^{1}H NMR (500 MHz, $CDCl_3$) δ 7.36 – 7.22 (m, 10H), 6.66 (s, 2H). ^{13}C NMR (101 MHz, $CDCl_3$) δ 137.34, 130.35, 128.97, 128.31, 127.19.

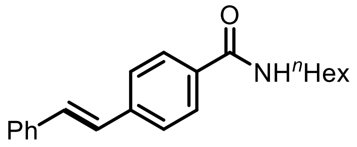

2l: Purified by washing with pentane, 97% isolated yield, E/Z = >99:1. IR (cm^{-1}): 3345, 2915, 1624, 1535. ^{1}H NMR (300 MHz, $CDCl_3$) δ 7.68 (d, *J* = 8.1 Hz, 2H), 7.45 (dd, *J* = 7.3, 5.6 Hz, 4H), 7.29 (t, *J* = 7.4 Hz, 2H), 7.24 – 7.17 (m, 1H), 7.16 – 6.94 (m, 2H), 6.22 (s, 1H), 3.36 (t, *J* = 7.0 Hz, 2H), 1.63 – 1.43 (m, 2H), 1.38 – 1.10 (m, 6H), 0.82 (t, *J* = 6.3 Hz, 3H). ^{13}C NMR (75 MHz, $CDCl_3$) δ 167.21, 167.13, 140.36, 136.91, 133.62, 130.62, 128.86, 128.20, 127.64, 127.43, 126.80, 126.58, 40.25, 40.12, 31.64, 29.75, 26.81, 22.69, 14.16. GC-EI-MS m/z calcd. for $C_{21}H_{23}NO_2$ $[M]^+$: 307.2, found: 307.2.

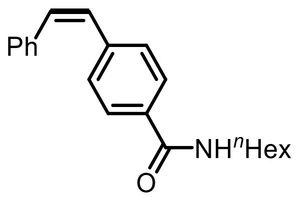

2l: Purified by flash column chromatograph, eluent: Hexane/EtOAc = 1:1. 99% isolated yield, full conversion at 29 h, Z/E = 11:1. IR (cm^{-1}): 3314, 2929, 1634, 1545. ^{1}H NMR (500 MHz, $CDCl_3$) δ 7.70 (d, *J* = 8.1 Hz, 2H), 7.35 (d, *J* = 8.5 Hz, 2H), 7.31 – 7.23 (m, 5H), 6.75 (d, *J* = 12.2 Hz, 1H), 6.66 (d, *J* = 12.2 Hz, 1H), 6.42 (br, 1H), 3.53 – 3.43 (m, 2H), 1.71 – 1.59 (m, 2H), 1.48 – 1.31 (m, 6H), 0.96 (t, *J* = 6.6 Hz, 3H). ^{13}C NMR (101 MHz, $CDCl_3$) δ 167.29, 140.34, 136.77, 133.24, 131.70, 129.23, 128.92, 128.84, 128.32, 127.41, 126.93, 40.14, 31.53, 29.63, 26.70, 22.57, 14.05. GC-EI-MS m/z calcd. for $C_{21}H_{23}NO_2$ $[M]^+$: 307.2, found: 307.2.

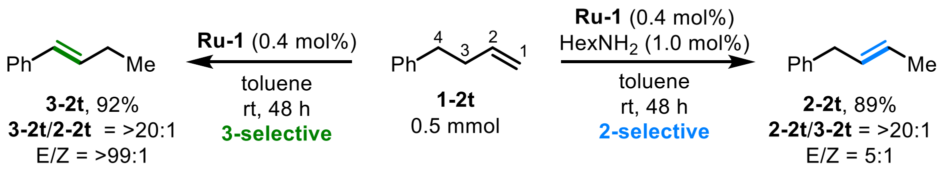

Isomerization of **1-2t** without amine additive: In a N_2-filled glovebox, a J-Young NMR tube was charged with **1-2t** (66.0 mg, 0.5 mmol), internal standard 1,3,5-trimethoxybenzene (42.0 mg, 0.25 mmol), **Ru-1** (1.2 mg, 2.0 μmol), toluene-d_8 (0.5 mL). The tube was taken out of the glovebox, and was rotated at room temperature. The progress of the isomerization was directly monitored by NMR, as shown below. Note: **3-2t** was generated after 5 h, when there was still a substantial amount of **2-2t** in the system.

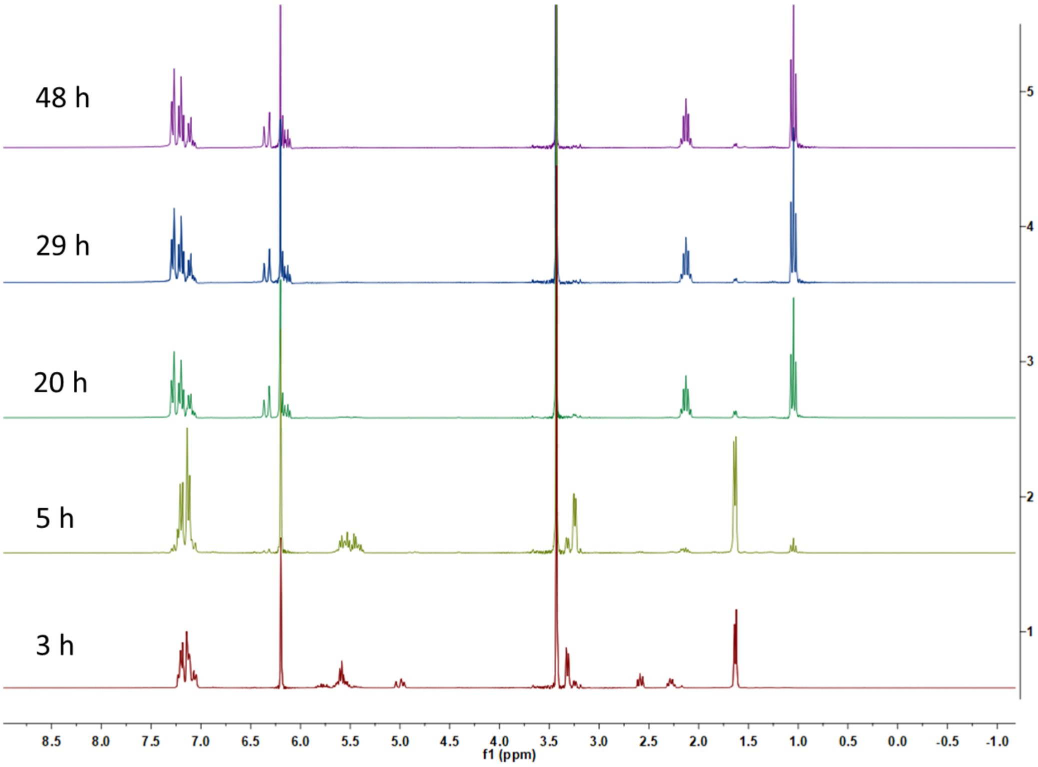

Figure S34. Monitoring the isomerization of **1-2t** without amine additive by ^{1}H NMR.

Isomerization of **1-2t** without amine additive: In a N_2-filled glovebox, a J-Young NMR tube was charged with internal standard 1,3,5-trimethoxybenzene (42.0 mg, 0.25 mmol), **Ru-1** (1.2 mg, 2.0 μmol), $HexNH_2$ (0.7 μL, 5.0 μmol), **1-2t** (66.0 mg, 0.5 mmol), toluene-d_8 (0.5 mL). The tube was taken out of the glovebox, and was rotated at room temperature. The progress of the isomerization was directly monitored by NMR, as shown below. Note: Detectable **3-2t** was generated only after 44 h.

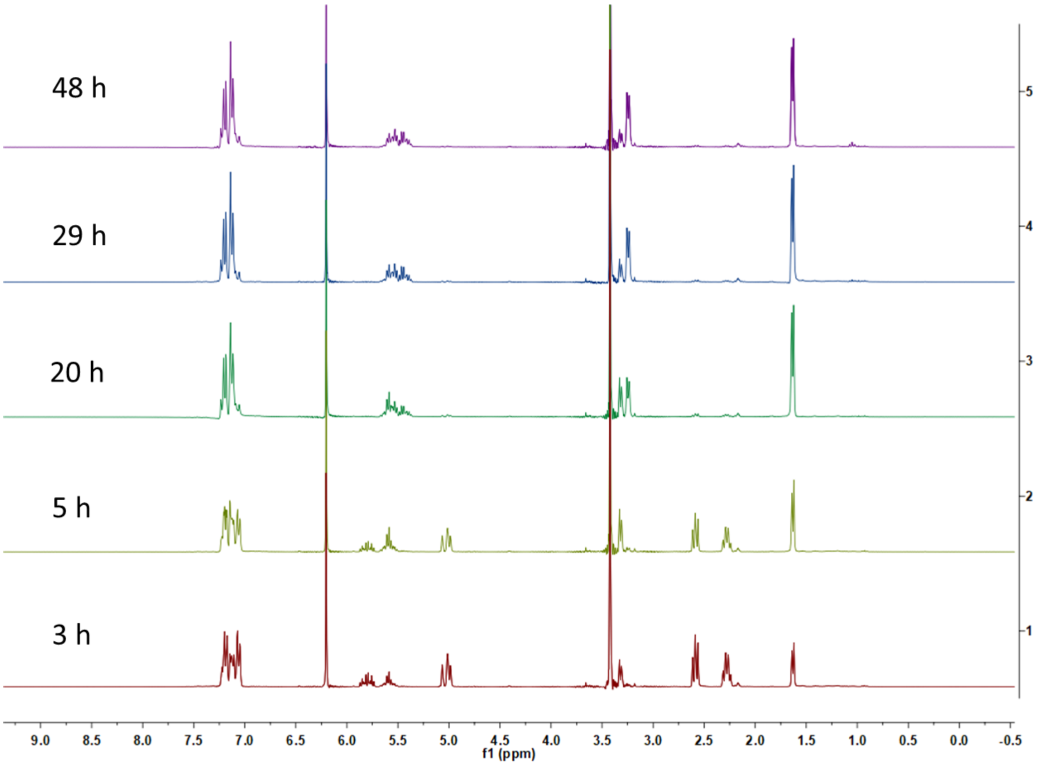

Figure S35. Monitoring the isomerization of **1-2t** with amine additive by ^{1}H NMR.

5. Selected NMR Spectra

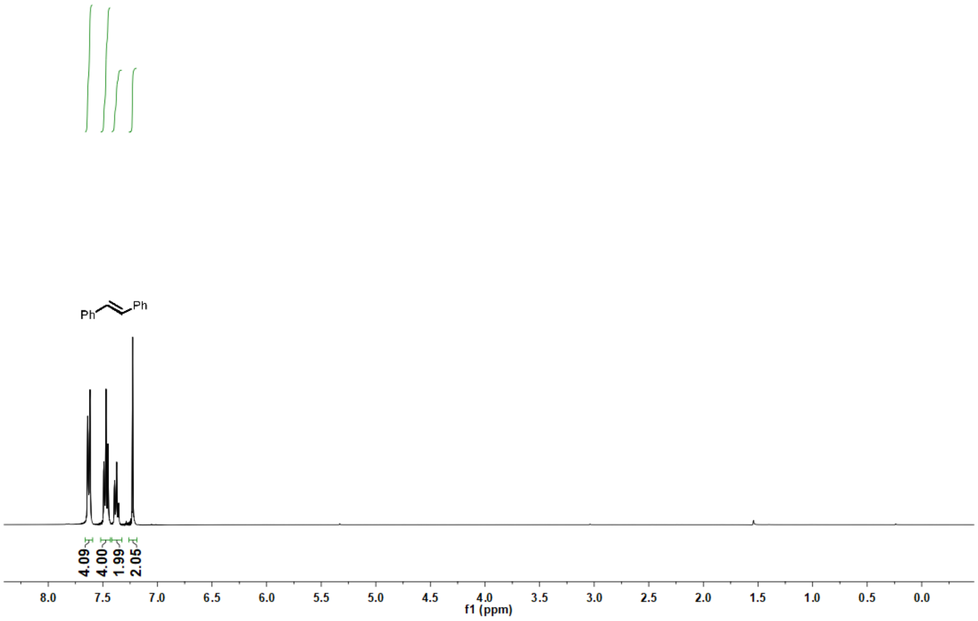

Figure S36. ^{1}H NMR ($CDCl_3$, 400 MHz) spectrum of isolated **2a** in a 20 mmol (E/Z = >99:1).

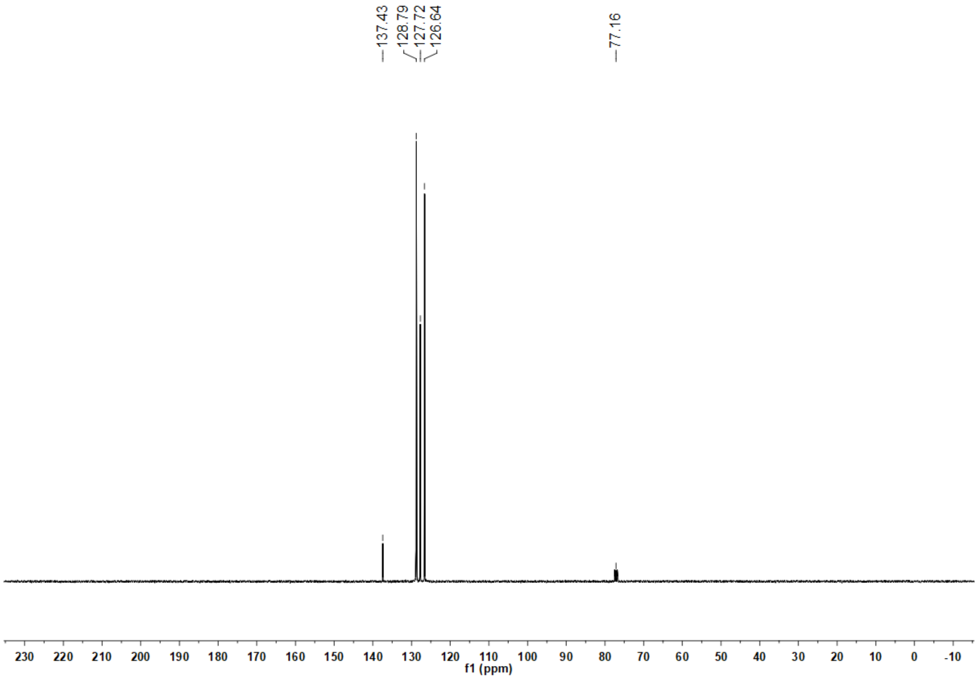

Figure S37. ^{13}C NMR ($CDCl_3$, 100 MHz) spectrum of isolated **2a** in a 20 mmol scale (E/Z = >99:1).

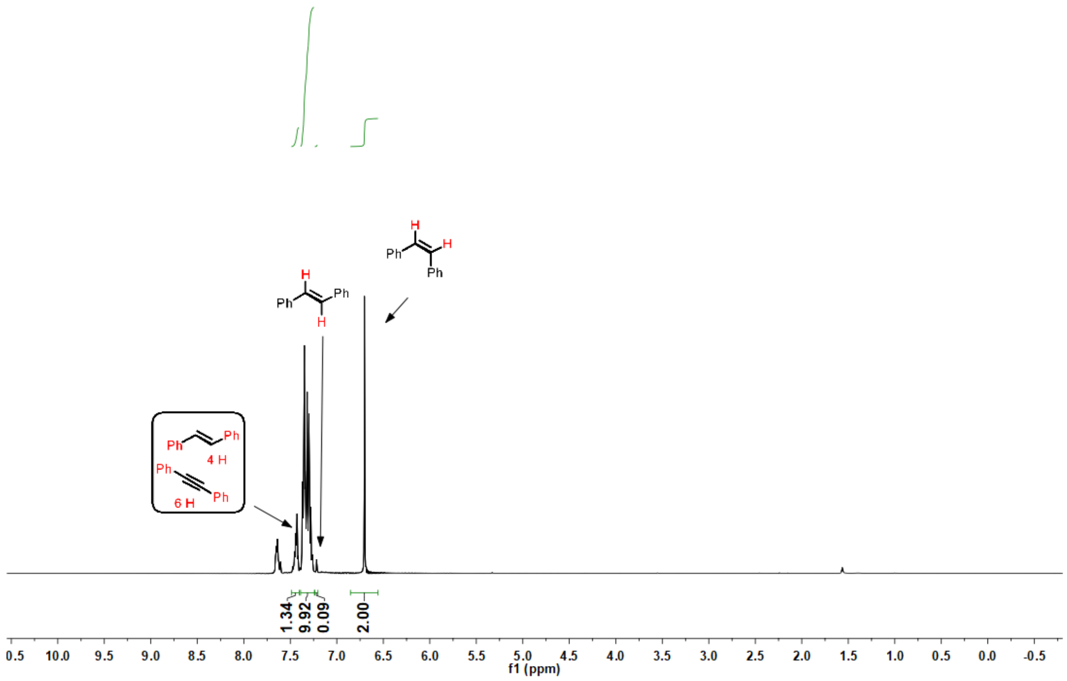

Figure S38. ^{1}H NMR ($CDCl_3$, 400 MHz) spectrum of **2a** after crude purification in a 20 mmol scale (with thiol, Z/E = >20:1).

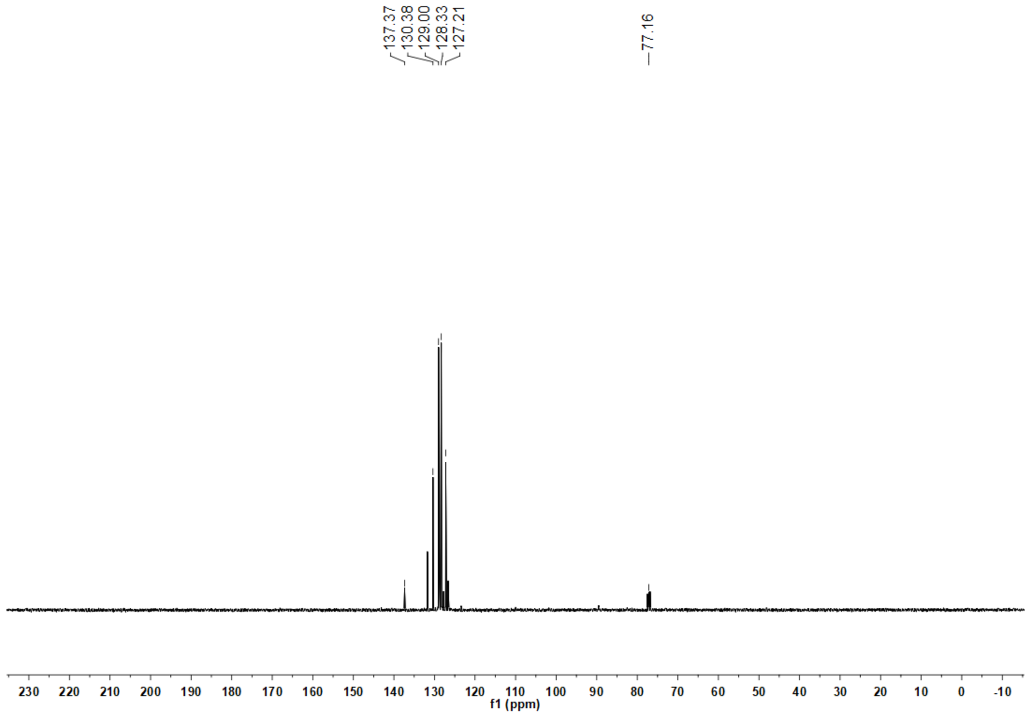

Figure S39. ^{13}C NMR ($CDCl_3$, 100 MHz) spectrum of of **2a** after crude purification in a 20 mmol scale (with thiol, Z/E = >20:1).

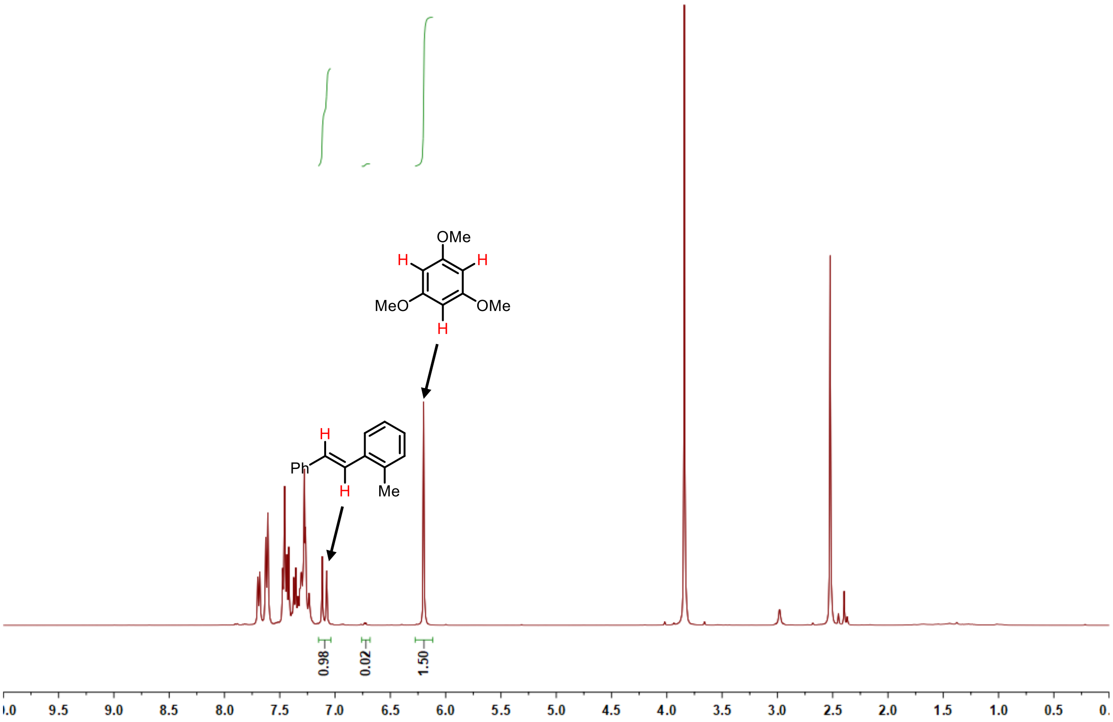

Figure S40. ^{1}H NMR ($CDCl_3$, 400 MHz) spectrum of the crude reaction mixture of hydrogenation of **1b** without thiol (E/Z = 49:1).

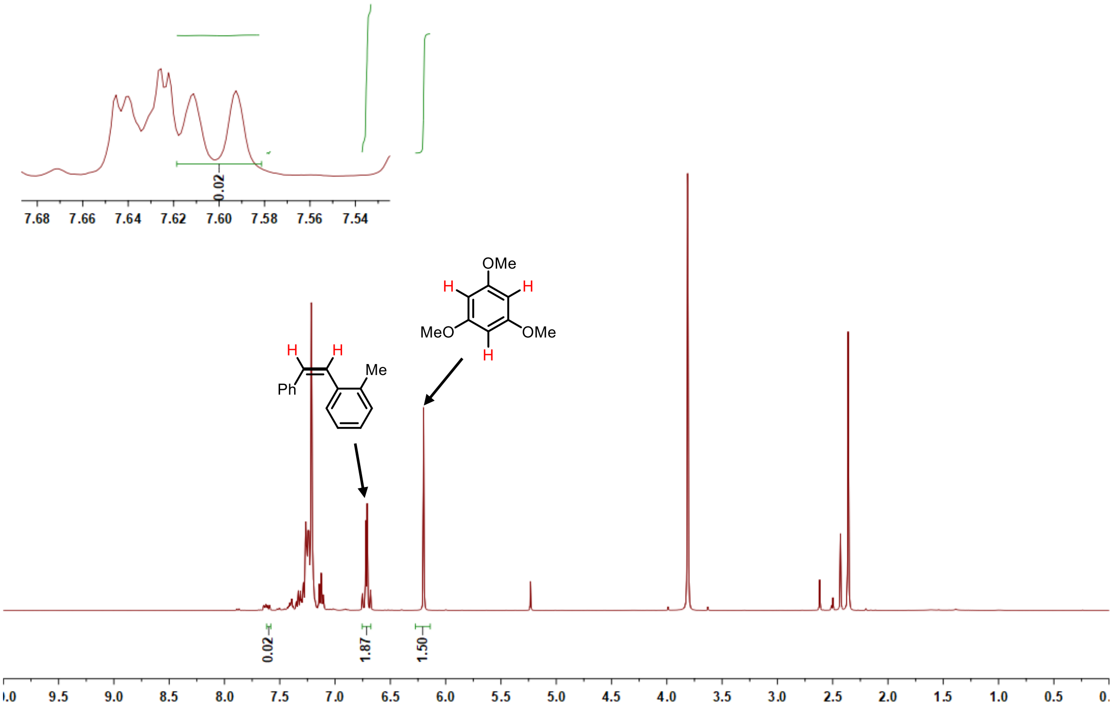

Figure S41. ^{1}H NMR ($CDCl_3$, 400 MHz) spectrum of the crude reaction mixture of hydrogenation of **1b** with thiol (Z/E = >20:1).

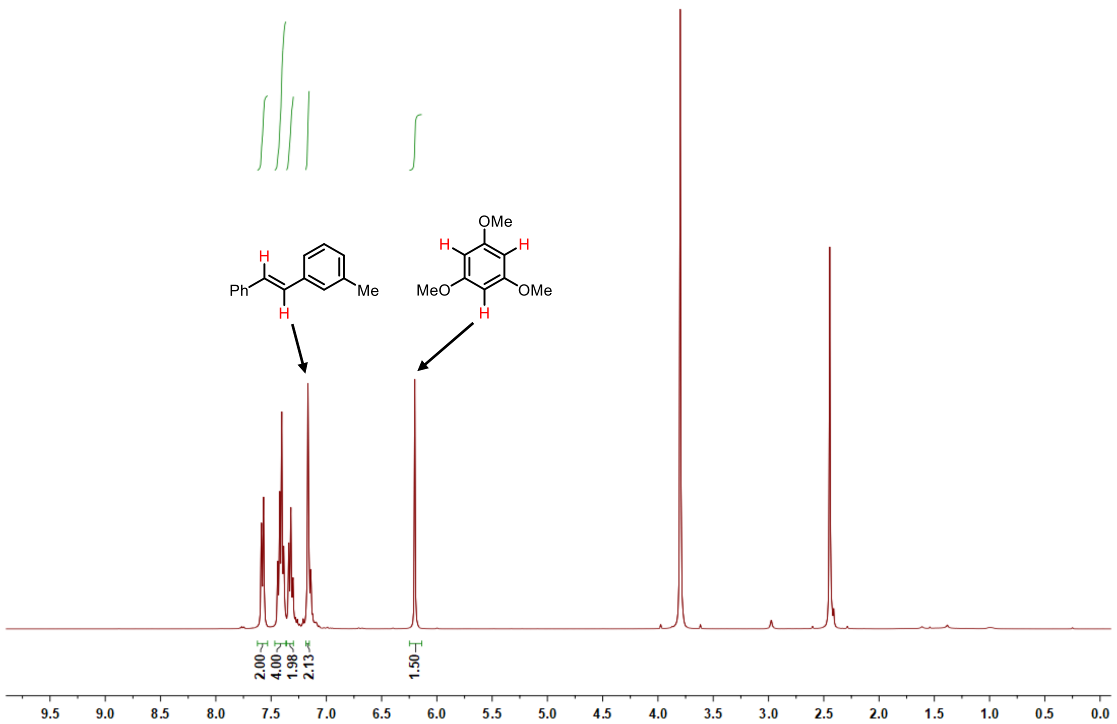

Figure S42. ^{1}H NMR ($CDCl_3$, 400 MHz) spectrum of the crude reaction mixture of hydrogenation of **1c** without thiol (E/Z = >99:1).

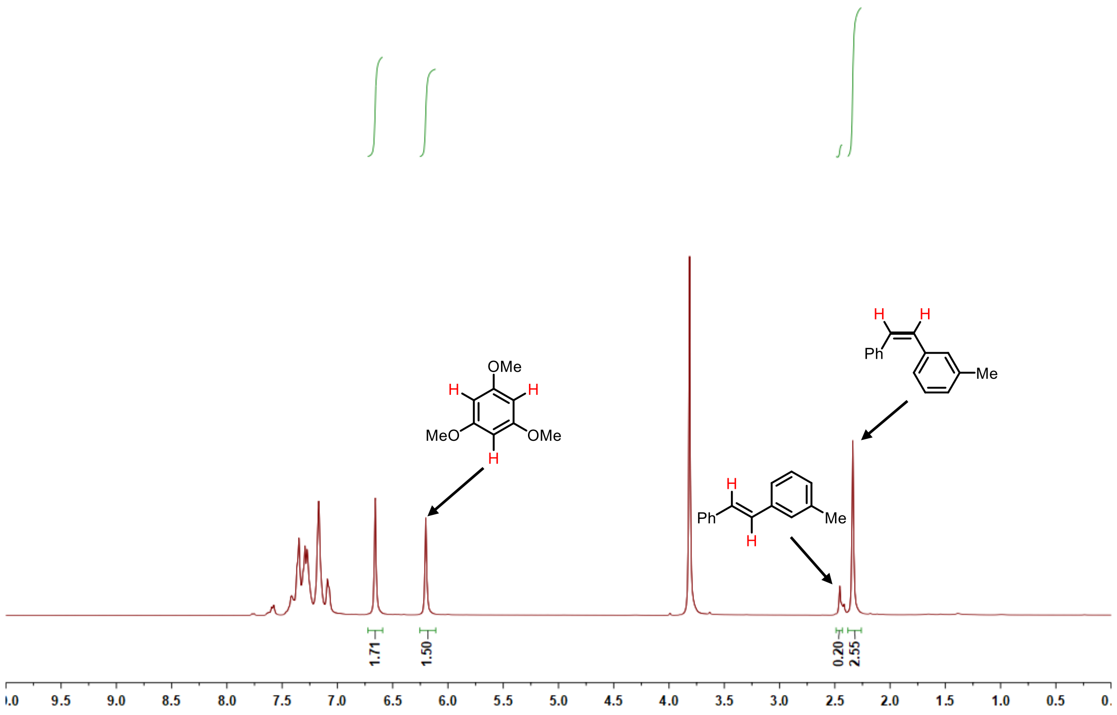

Figure S43. ^{1}H NMR ($CDCl_3$, 400 MHz) spectrum of the crude reaction mixture of hydrogenation of **1c** with thiol (Z/E = 12:1).

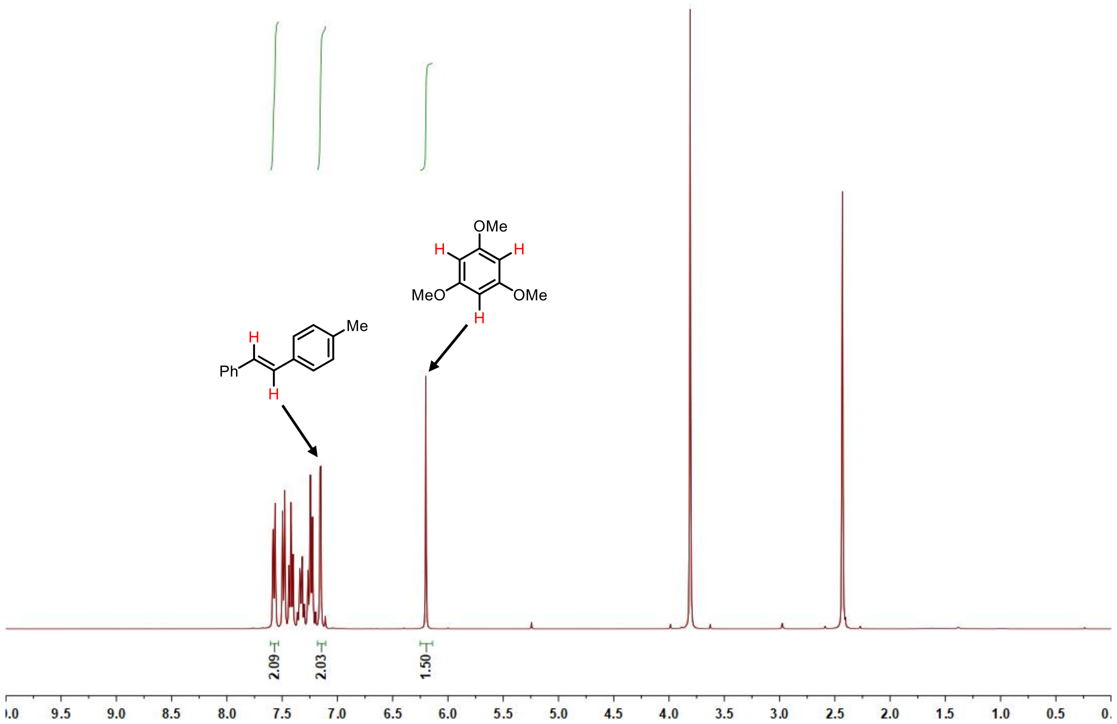

Figure S44. ^{1}H NMR ($CDCl_3$, 400 MHz) spectrum of the crude reaction mixture of hydrogenation of **1d** without thiol (E/Z = >99:1).

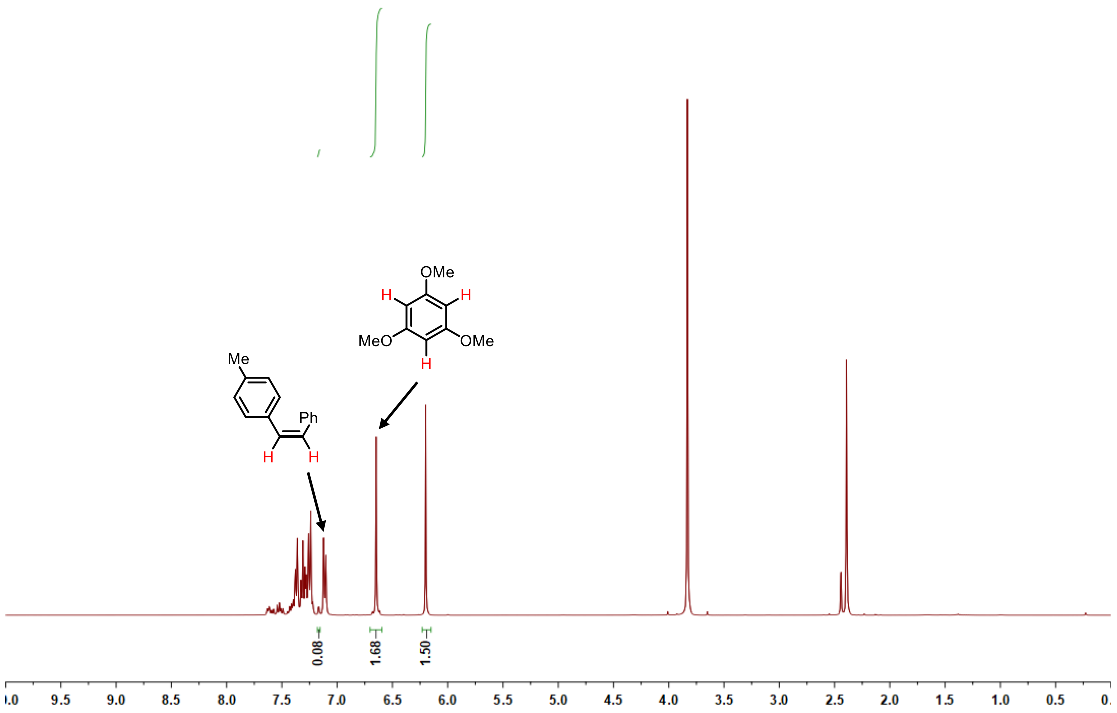

Figure S45. ^{1}H NMR ($CDCl_3$, 400 MHz) spectrum of the crude reaction mixture of hydrogenation of **1d** with thiol (Z/E = >20:1).

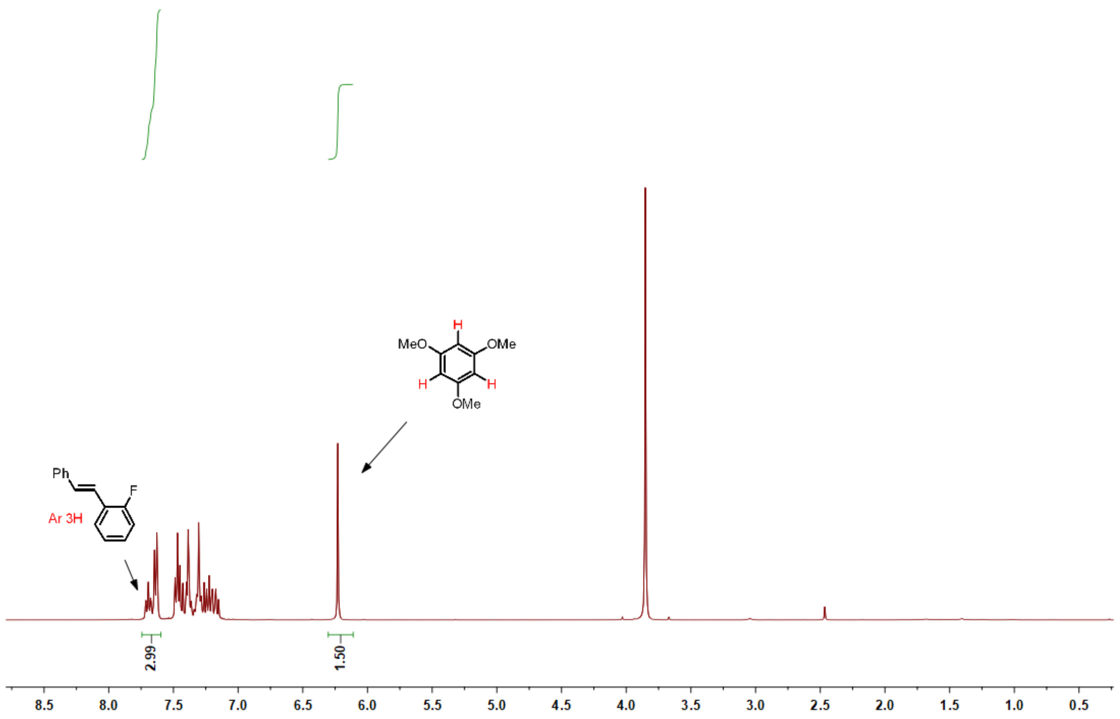

Figure S46. ^{1}H NMR ($CDCl_3$, 400 MHz) spectrum of the crude reaction mixture of hydrogenation of **1e** without thiol (E/Z = >99:1).

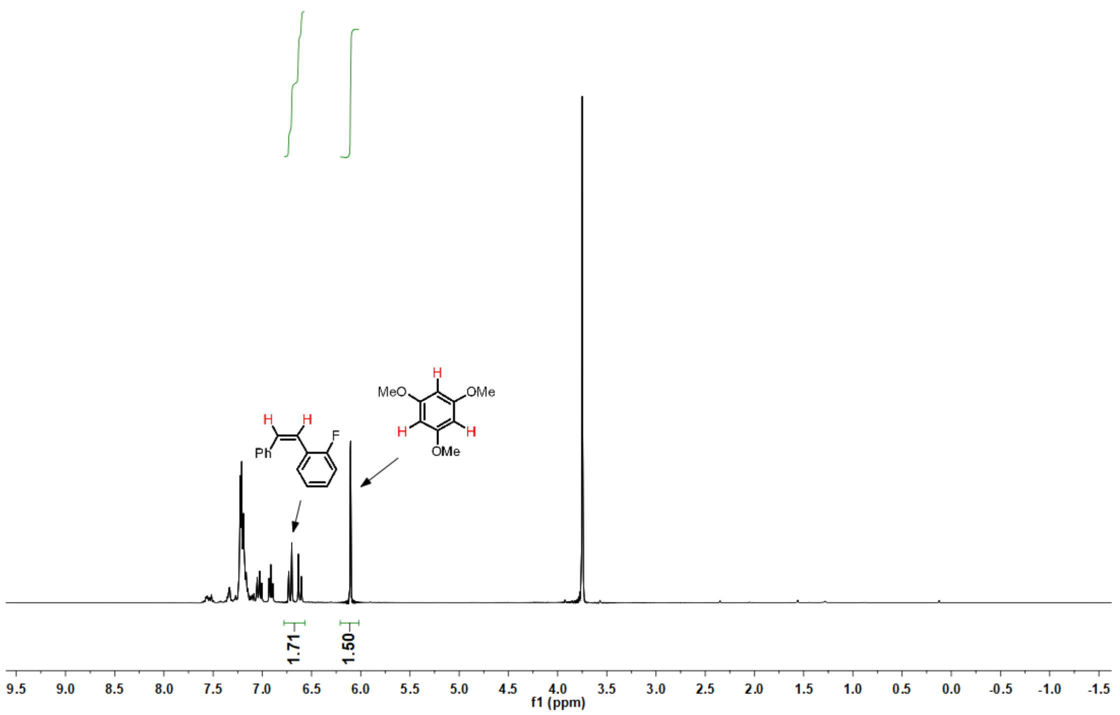

Figure S47. ^{1}H NMR ($CDCl_3$, 400 MHz) spectrum of the crude reaction mixture of hydrogenation of **1e** with thiol (85% Z-**2e**).

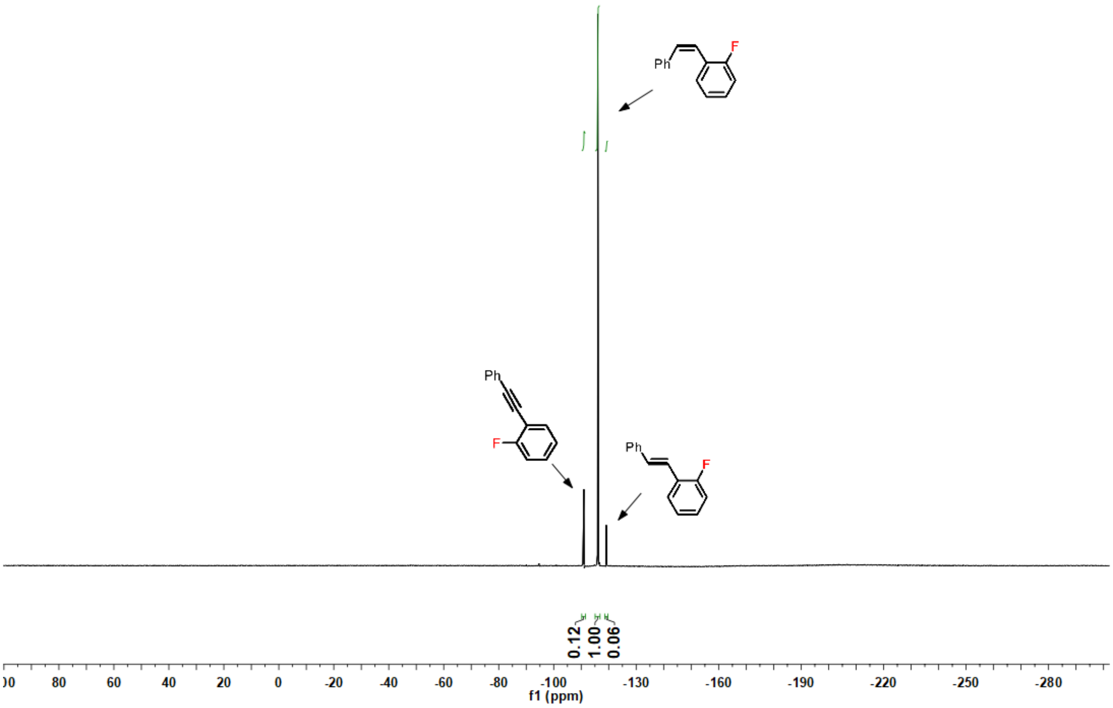

Figure S48. ^{19}F NMR ($CDCl_3$, 282 MHz) spectrum of the crude reaction mixture of hydrogenation of **1e** with thiol (Z/E = 17:1).

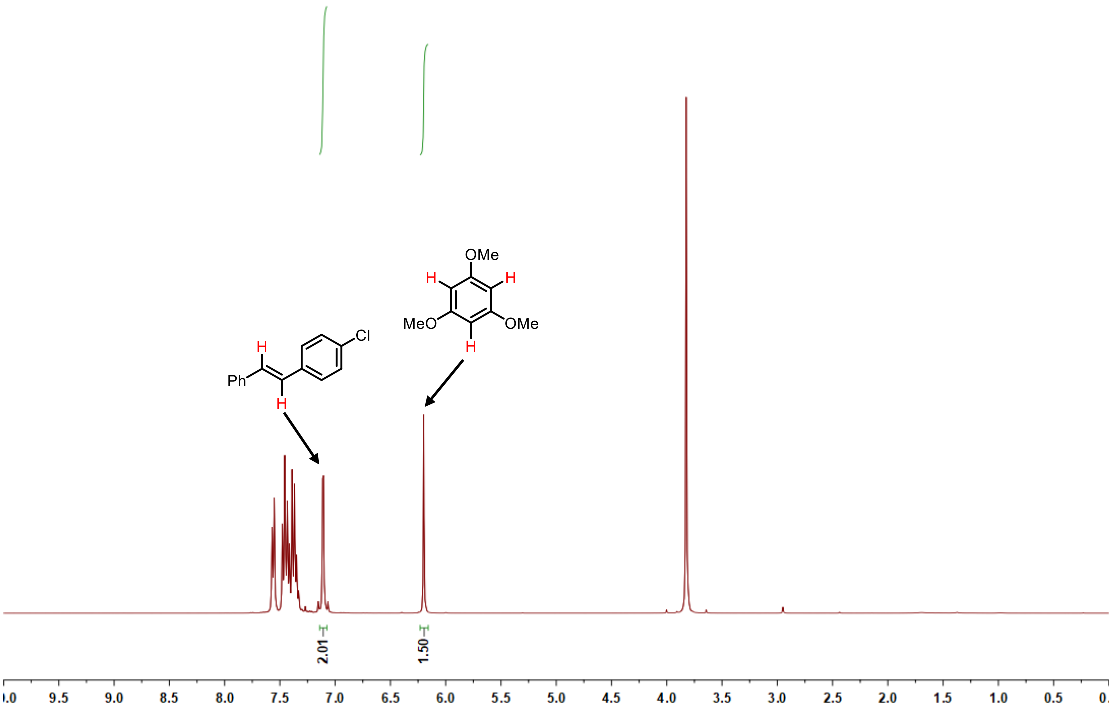

Figure S49. ^{1}H NMR ($CDCl_3$, 400 MHz) spectrum of the crude reaction mixture of hydrogenation of **1f** without thiol (E/Z = >99:1).

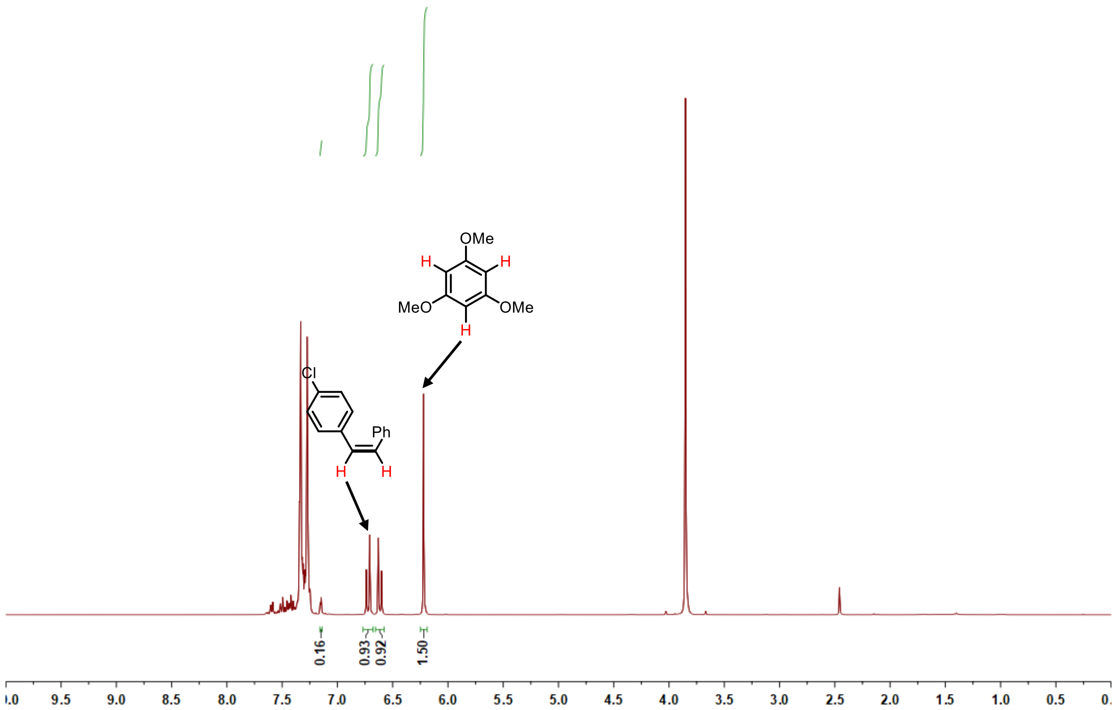

Figure S50. ^{1}H NMR ($CDCl_3$, 400 MHz) spectrum of the crude reaction mixture of hydrogenation of **1f** with thiol (Z/E = 12:1).

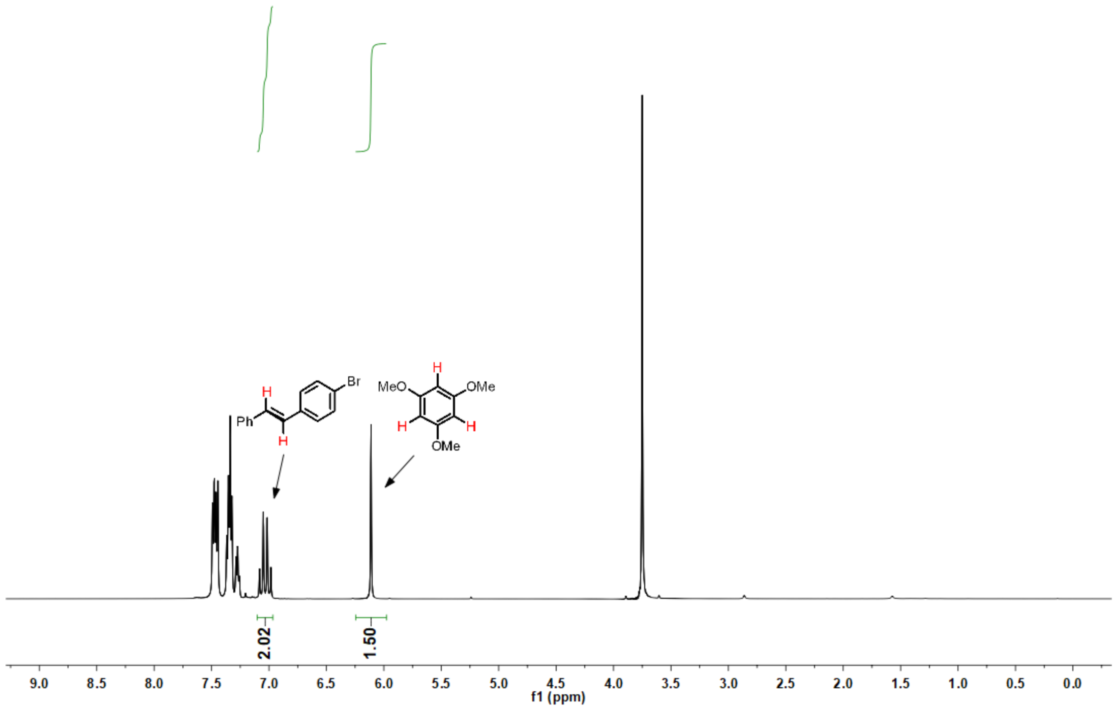

Figure S51. ^{1}H NMR ($CDCl_3$, 400 MHz) spectrum of the crude reaction mixture of hydrogenation of **1g** without thiol (E/Z = >99:1).

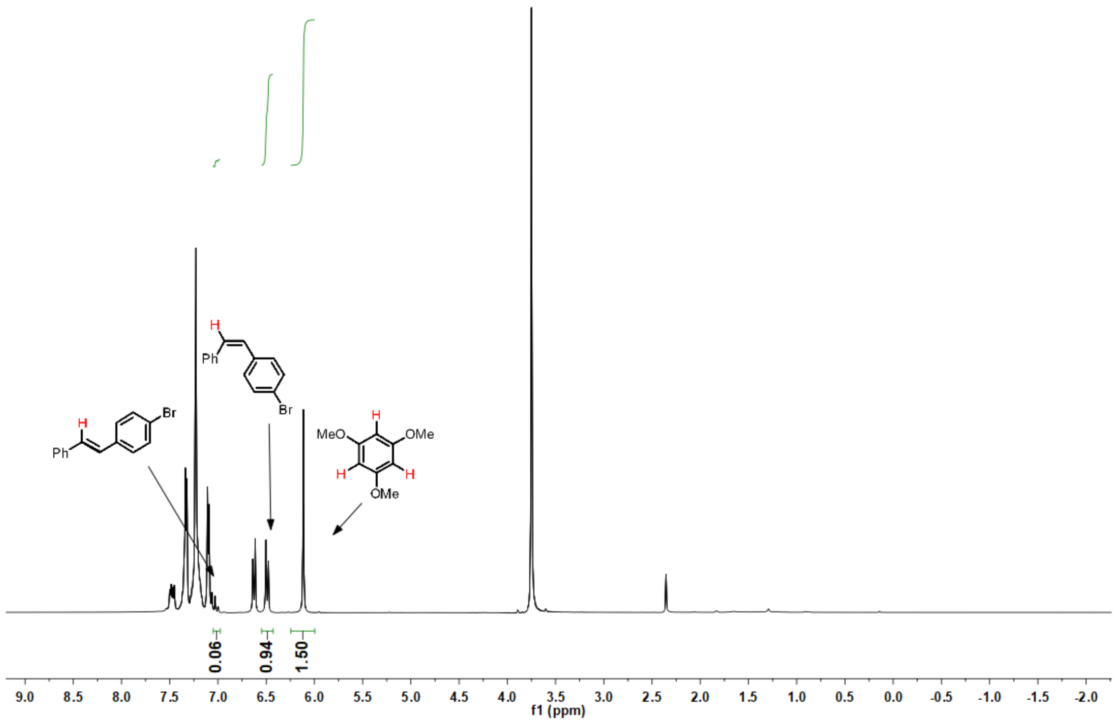

Figure S52. ^{1}H NMR ($CDCl_3$, 500 MHz) spectrum of the crude reaction mixture of hydrogenation of **1g** with thiol (Z/E=16:1).

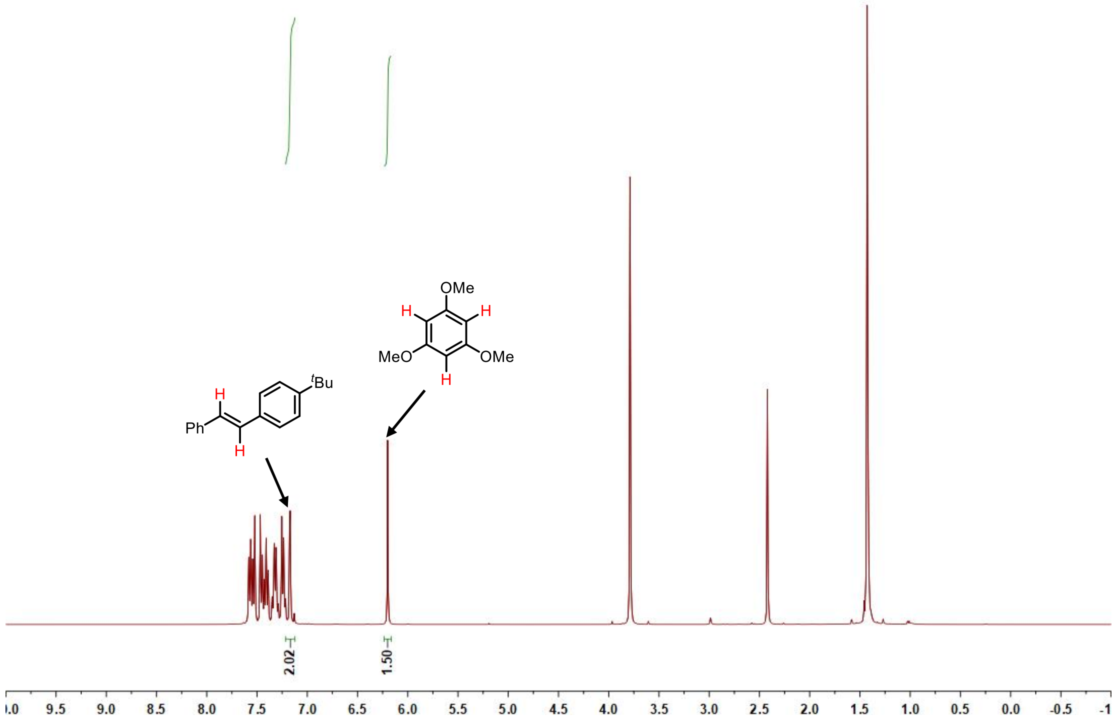

Figure S53. ^{1}H NMR ($CDCl_3$, 400 MHz) spectrum of the crude reaction mixture of hydrogenation of **1h** without thiol (E/Z = >99:1).

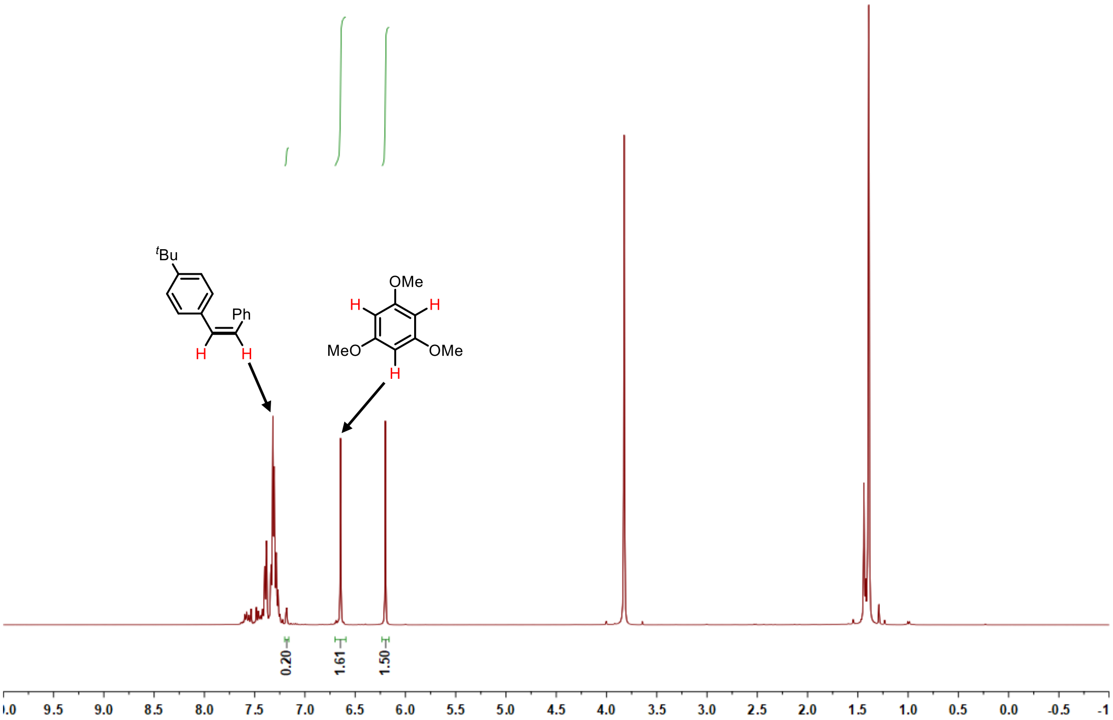

Figure S54. ^{1}H NMR ($CDCl_3$, 400 MHz) spectrum of the crude reaction mixture of hydrogenation of **1h** with thiol (Z/E = 8:1).

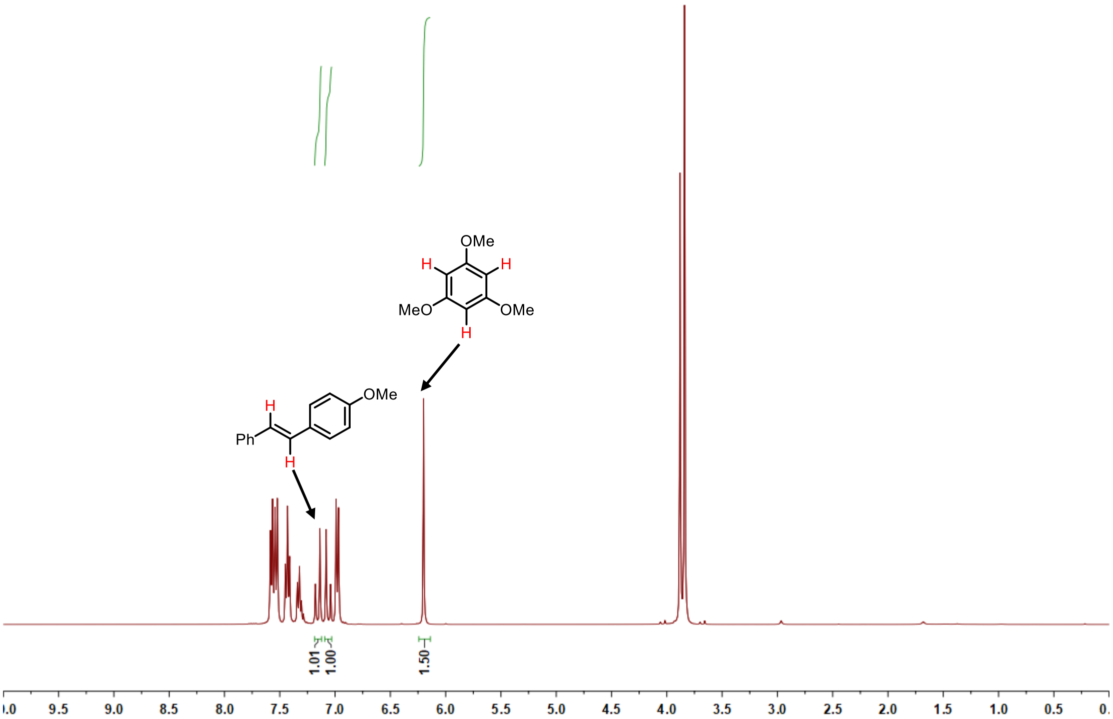

Figure S55. ^{1}H NMR ($CDCl_3$, 400 MHz) spectrum of the crude reaction mixture of hydrogenation of **1i** without thiol (E/Z = >99:1).

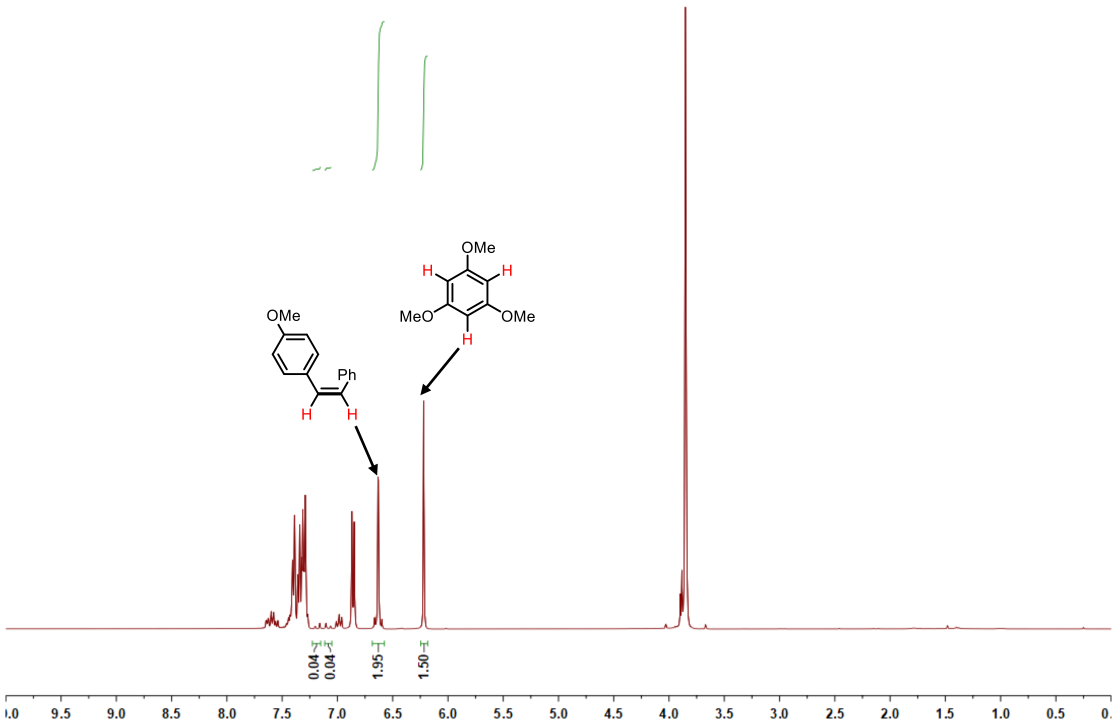

Figure S56. ^{1}H NMR ($CDCl_3$, 400 MHz) spectrum of the crude reaction mixture of hydrogenation of **1i** with thiol (Z/E>20:1).

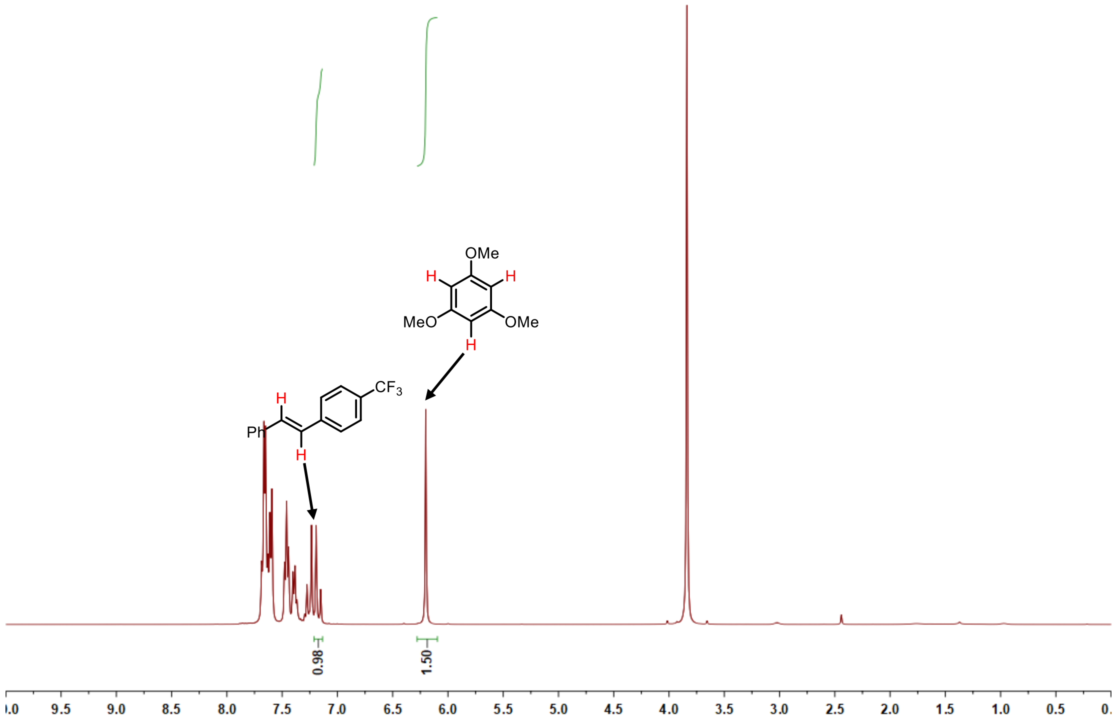

Figure S57. ^{1}H NMR ($CDCl_3$, 400 MHz) spectrum of the crude reaction mixture of hydrogenation of **1j** without thiol (E/Z = >99:1).

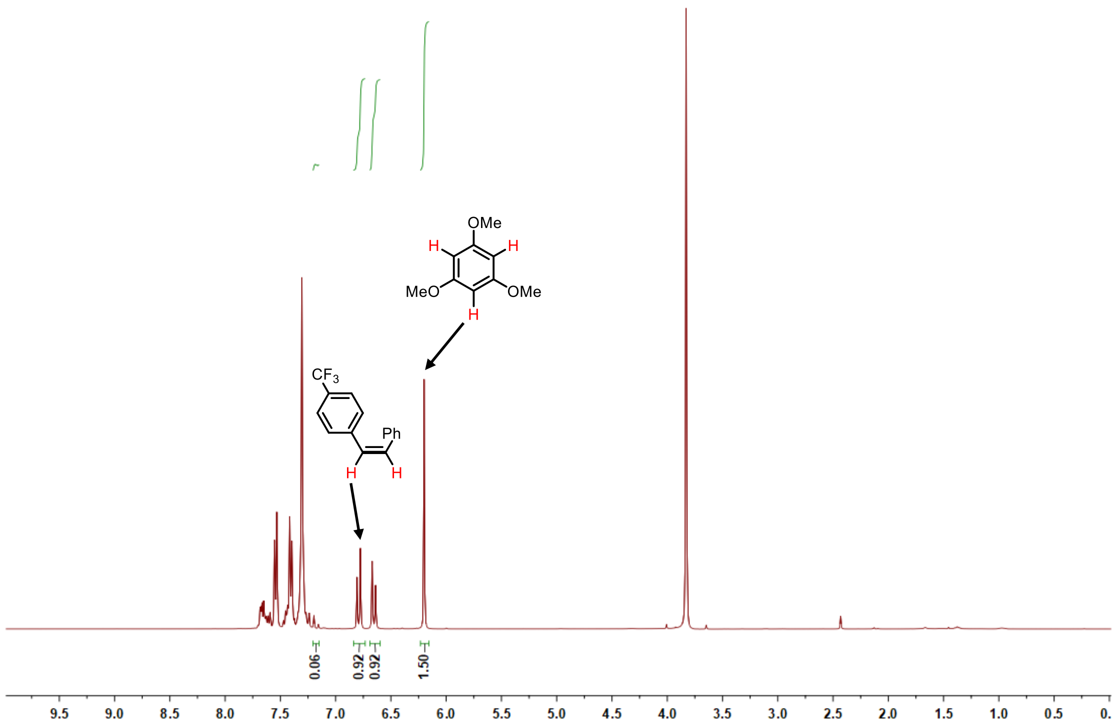

Figure S58. ^{1}H NMR ($CDCl_3$, 400 MHz) spectrum of the crude reaction mixture of hydrogenation of **1j** with thiol (Z/E = 15:1).

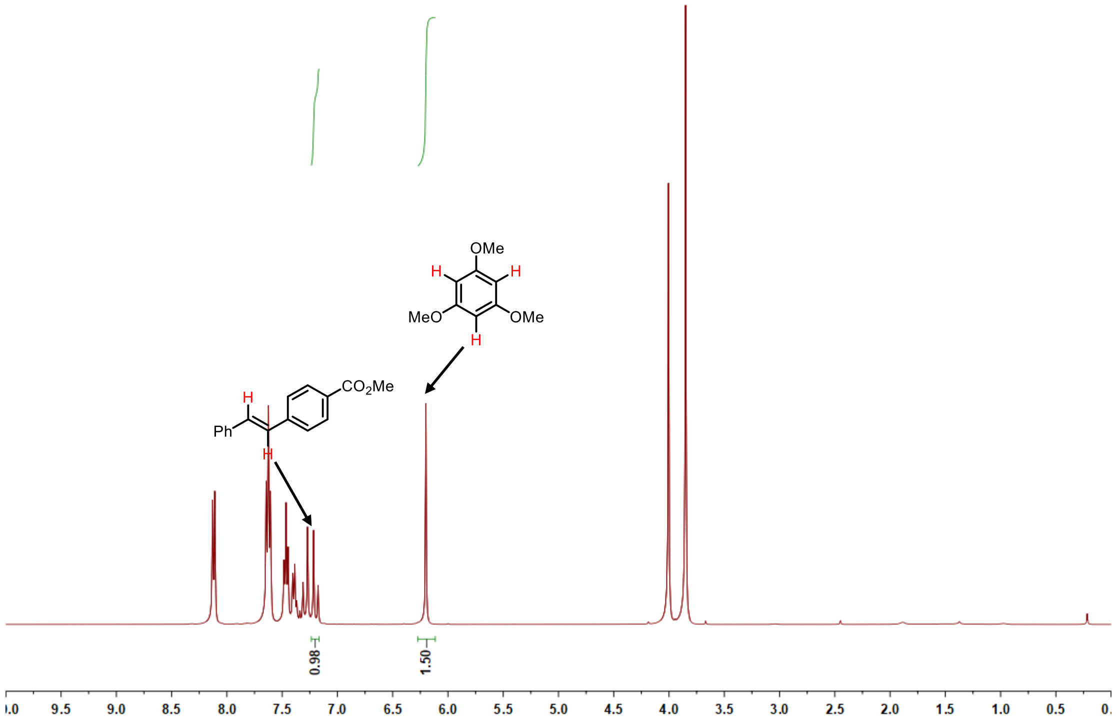

Figure S59. ^{1}H NMR ($CDCl_3$, 400 MHz) spectrum of the crude reaction mixture of hydrogenation of **1k** without thiol (E/Z = >99:1).

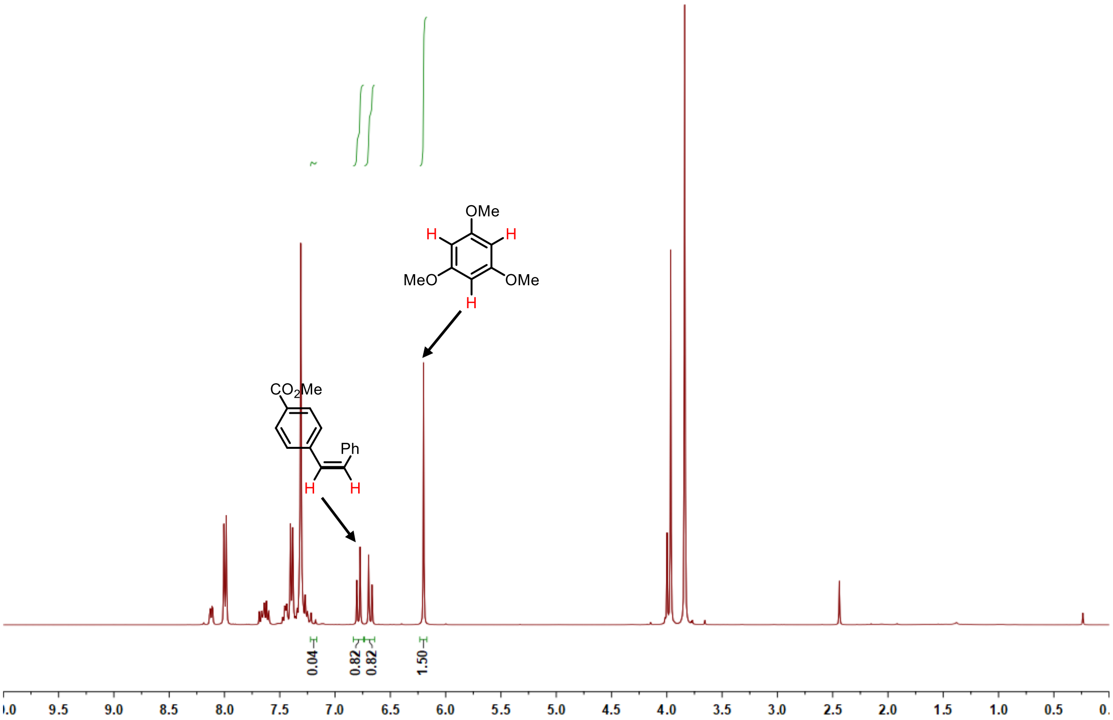

Figure S60. ^{1}H NMR ($CDCl_3$, 400 MHz) spectrum of the crude reaction mixture of hydrogenation of **1k** with thiol (Z/E = >20:1).

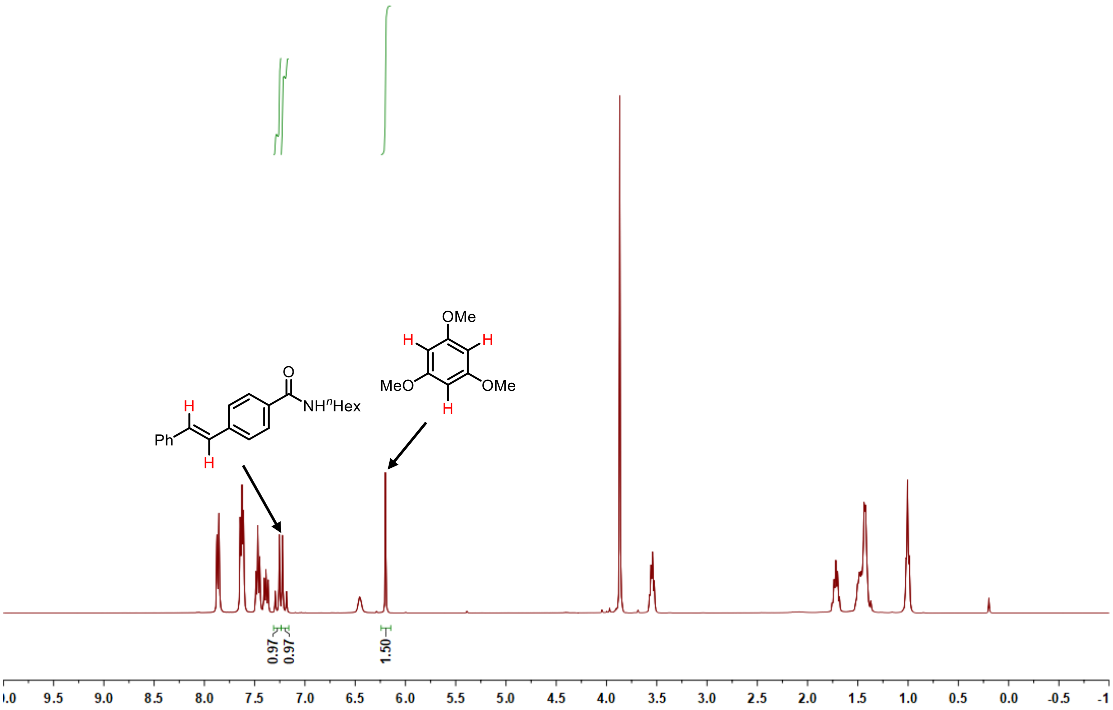

Figure S61. ^{1}H NMR ($CDCl_3$, 400 MHz) spectrum of the crude reaction mixture of hydrogenation of **1l** without thiol (E/Z = >99:1).

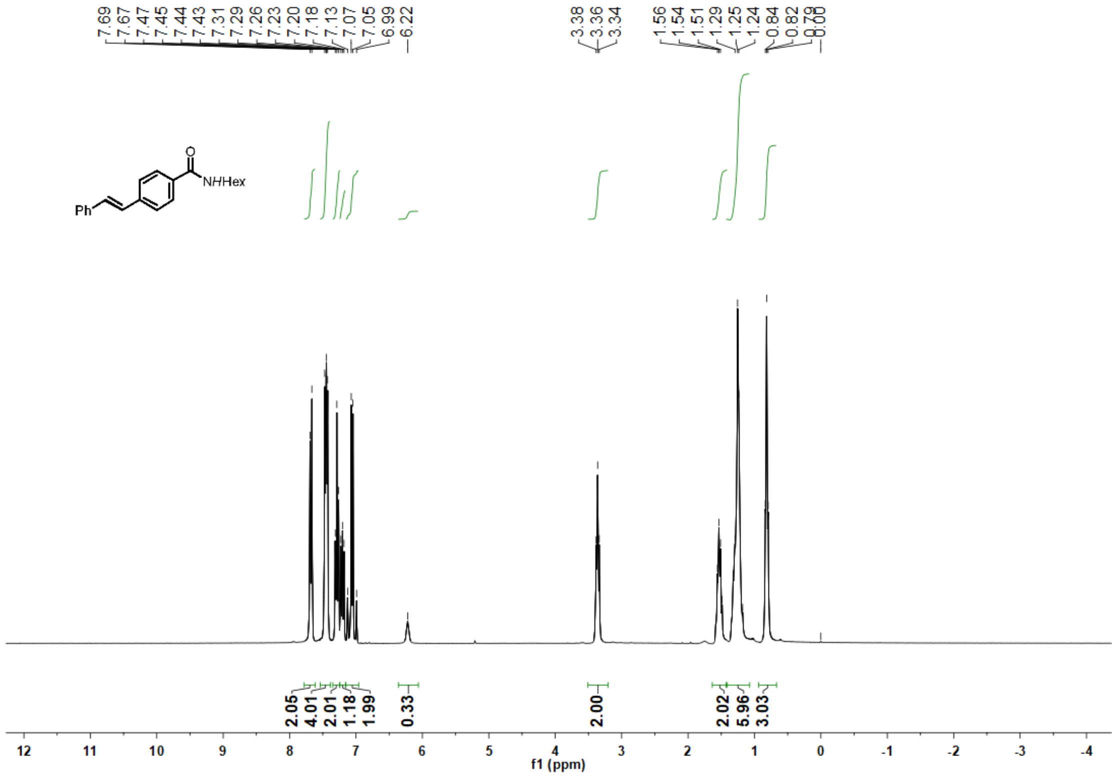

Figure S62. ^{1}H NMR ($CDCl_3$, 300 MHz) spectrum of isolated **2l** (without thiol, E/Z = >99:1).

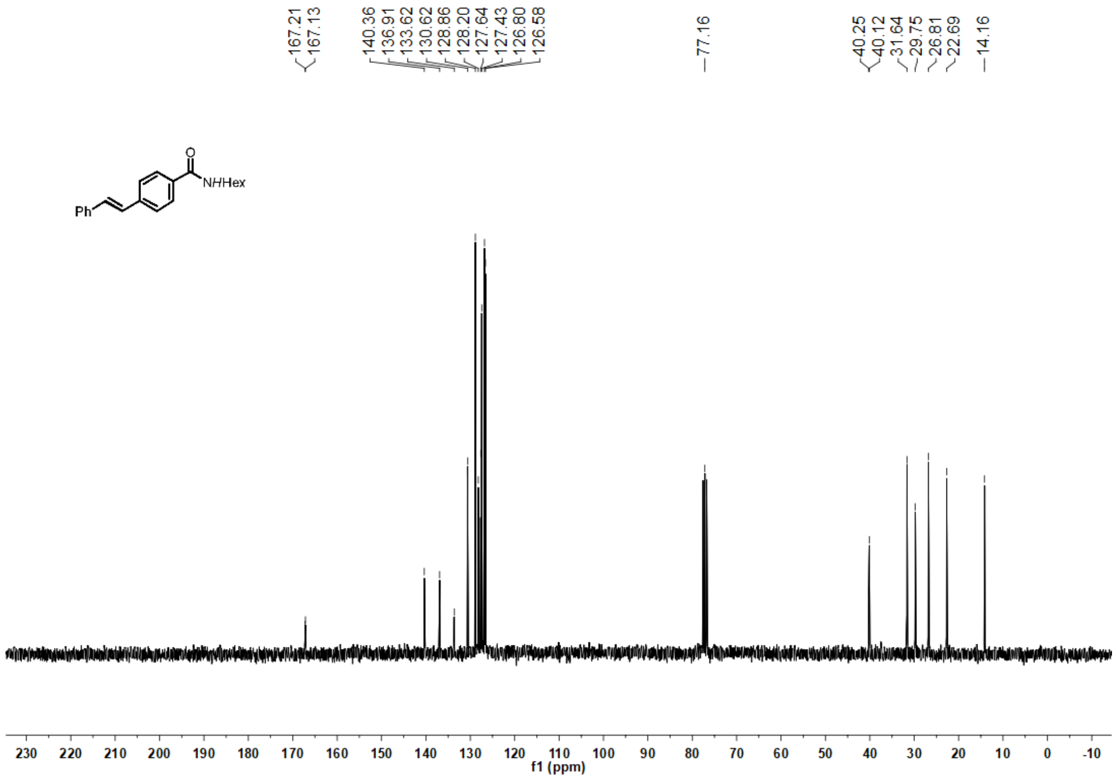

Figure S63. ^{13}C NMR ($CDCl_3$, 75 MHz) spectrum of isolated **2l** (without thiol, E/Z = >99:1).

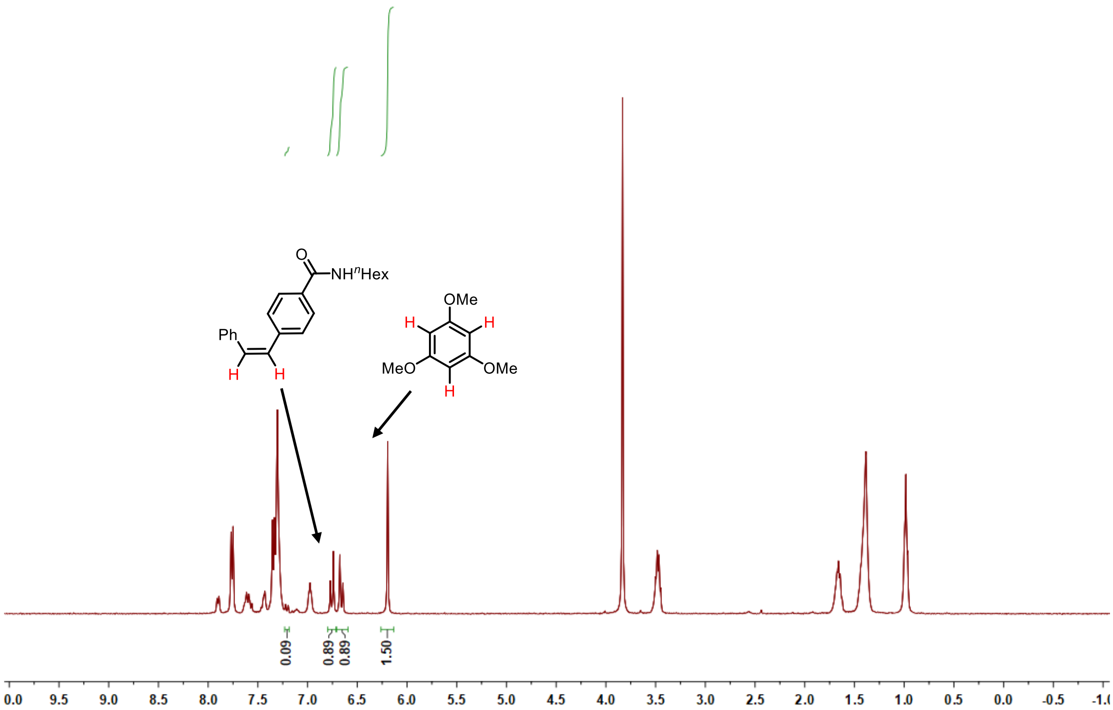

Figure S64. ^{1}H NMR ($CDCl_3$, 400 MHz) spectrum of the crude reaction mixture of hydrogenation of **1l** with thiol (Z/E = 20:1).

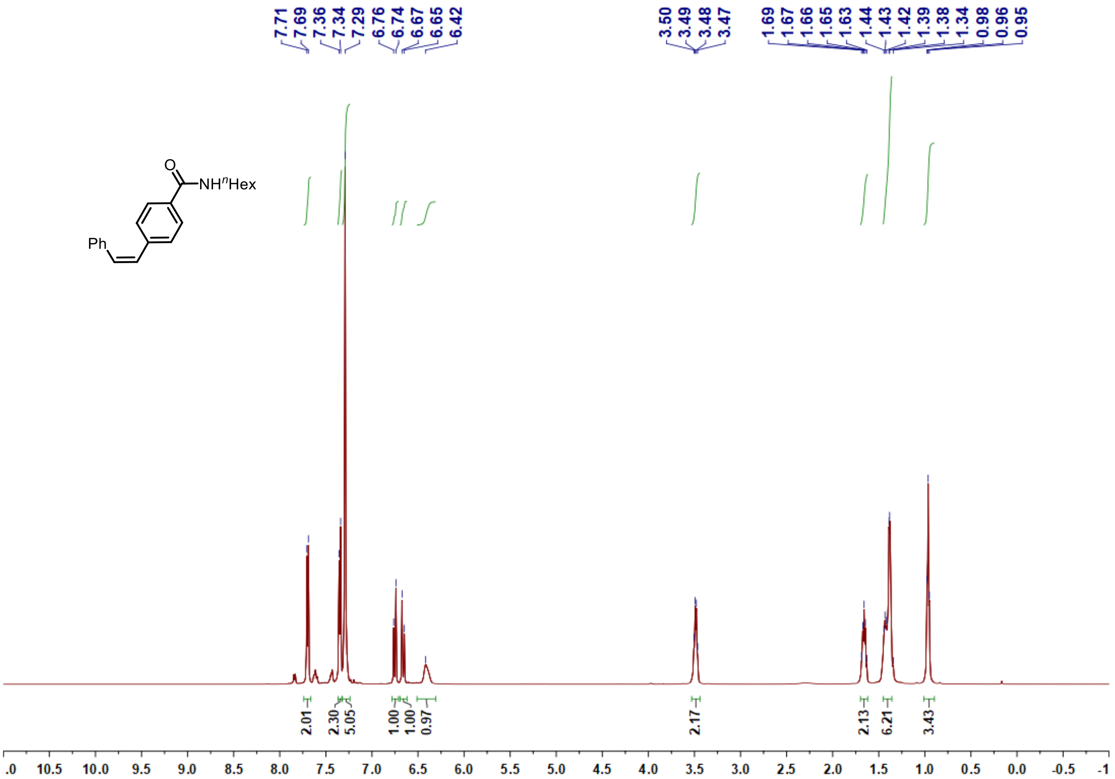

Figure S65. ^{1}H NMR ($CDCl_3$, 400 MHz) spectrum of isolated **2l** (with thiol, Z/E = 11:1, obtained after 29 h with full conversion of **1l**).

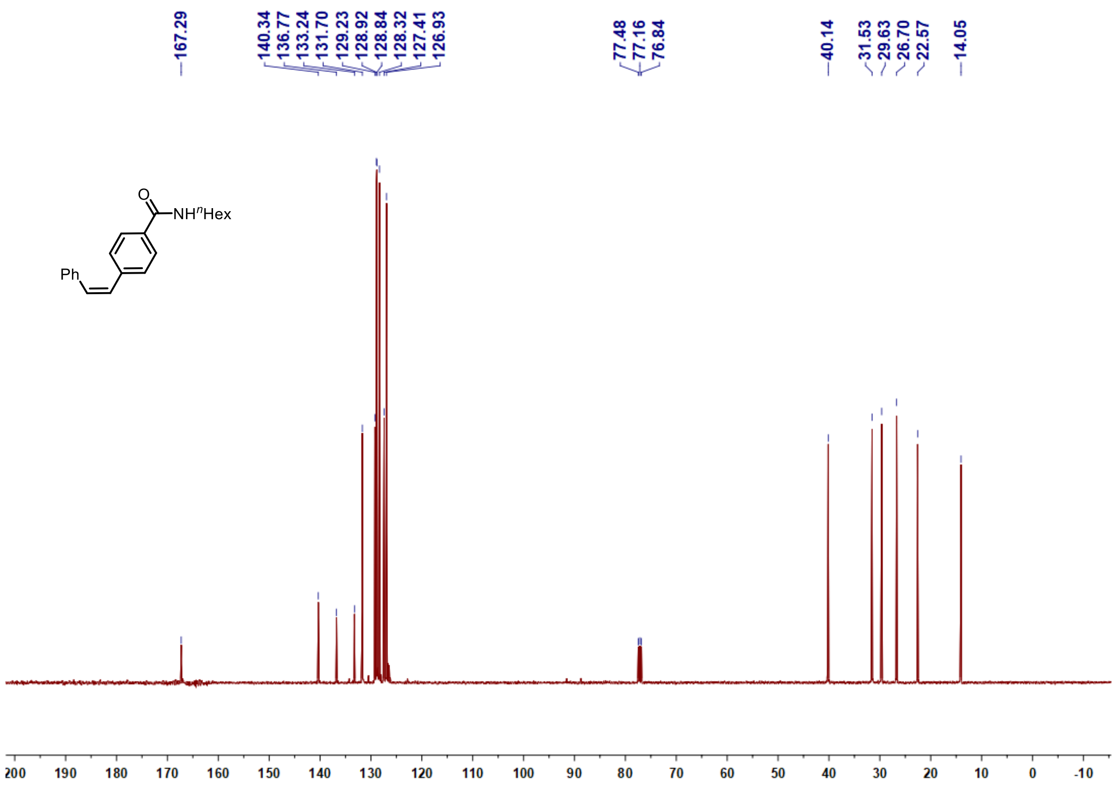

Figure S66. ^{13}C NMR ($CDCl_3$, 75 MHz) spectrum of isolated **2l** (with thiol, Z/E = 11:1, obtained after 29 h with full conversion of **1l**).

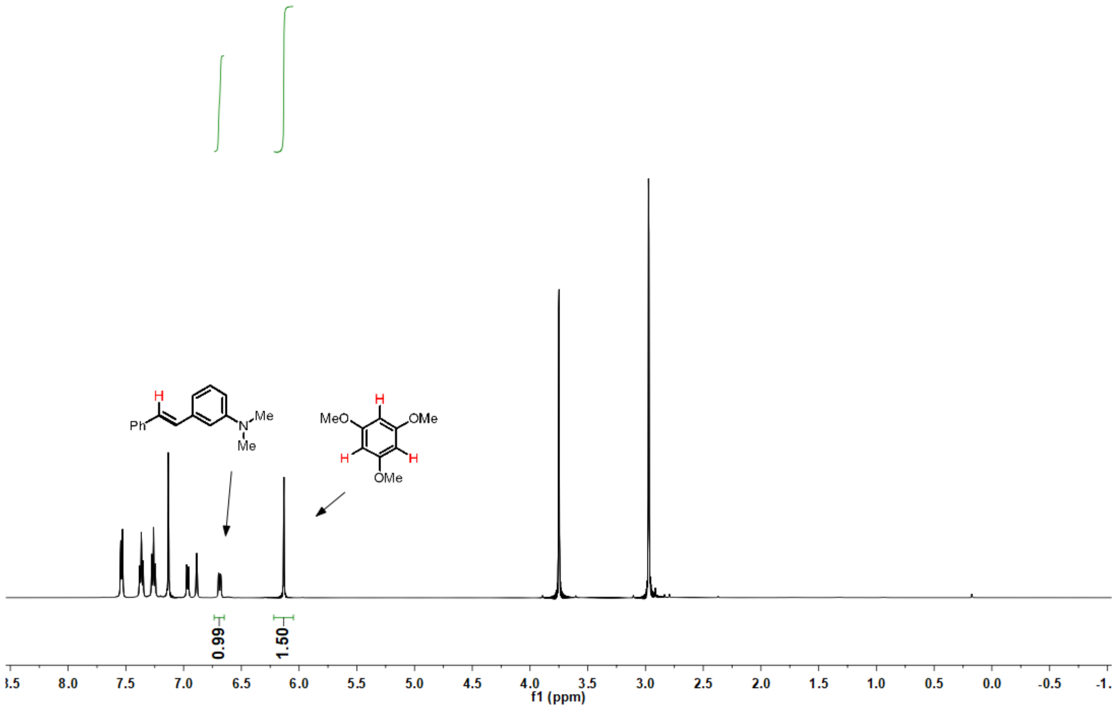

Figure S67. ^{1}H NMR ($CDCl_3$, 500 MHz) spectrum of the crude reaction mixture of hydrogenation of **1m** without thiol (E/Z = >99:1).

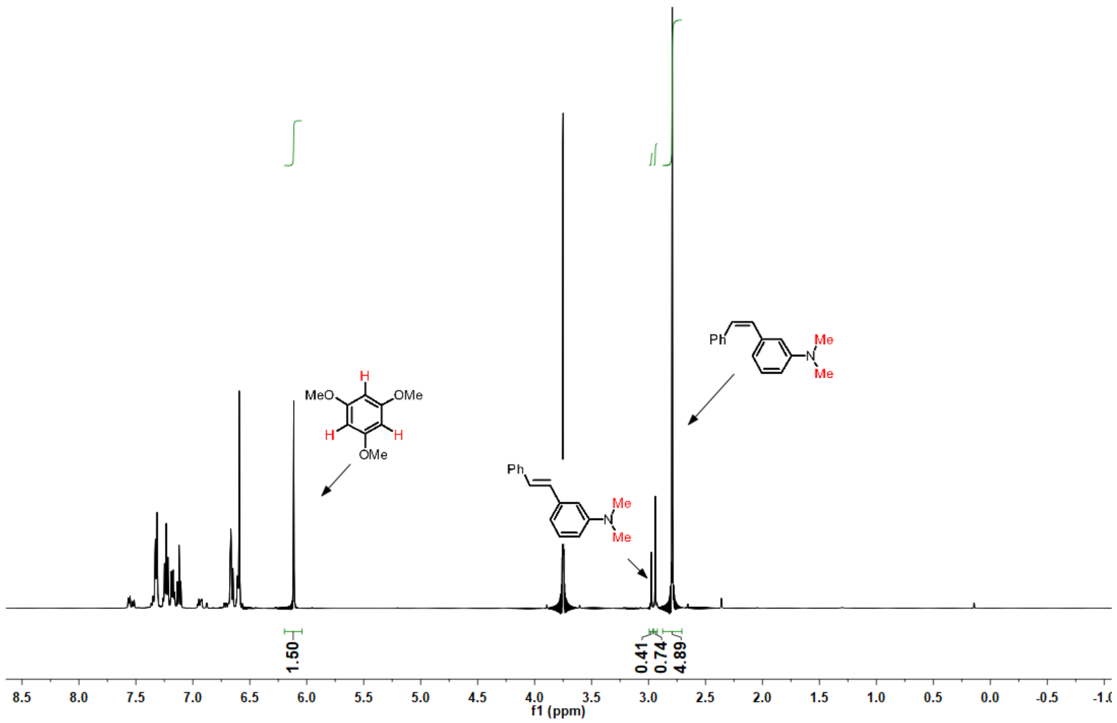

Figure S68. ^{1}H NMR ($CDCl_3$, 500 MHz) spectrum of the crude reaction mixture of hydrogenation of **1m** with thiol (Z/E = 12:1).

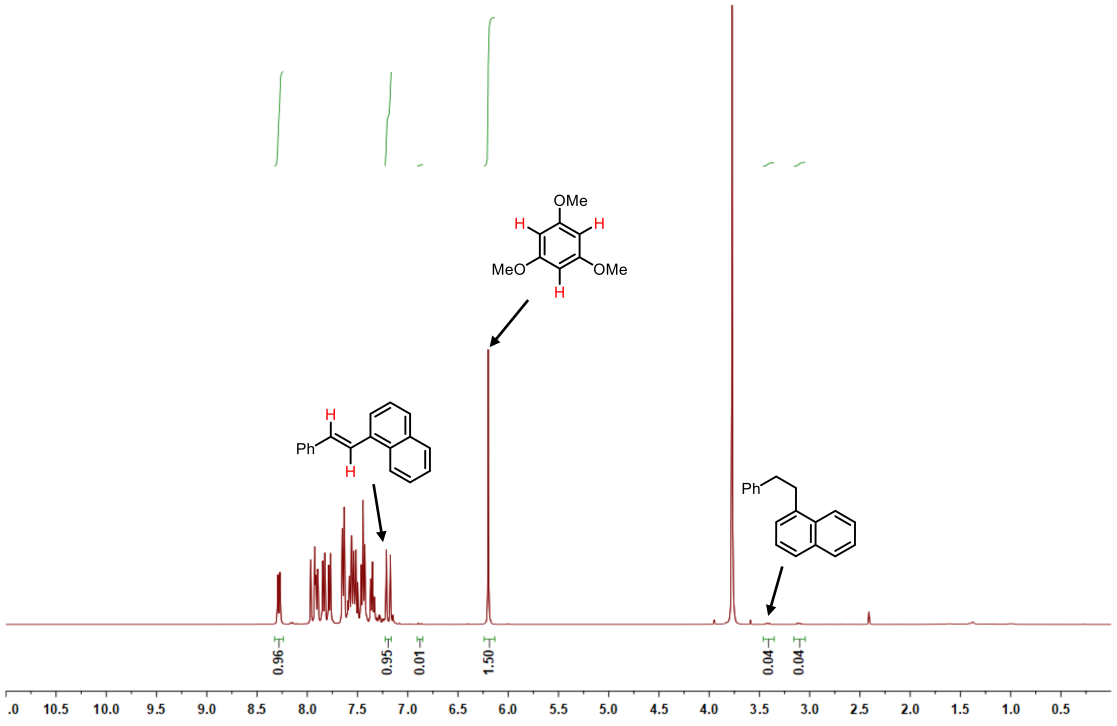

Figure S69. ^{1}H NMR ($CDCl_3$, 400 MHz) spectrum of the crude reaction mixture of hydrogenation of **1n** without thiol (E/Z = 96:1).

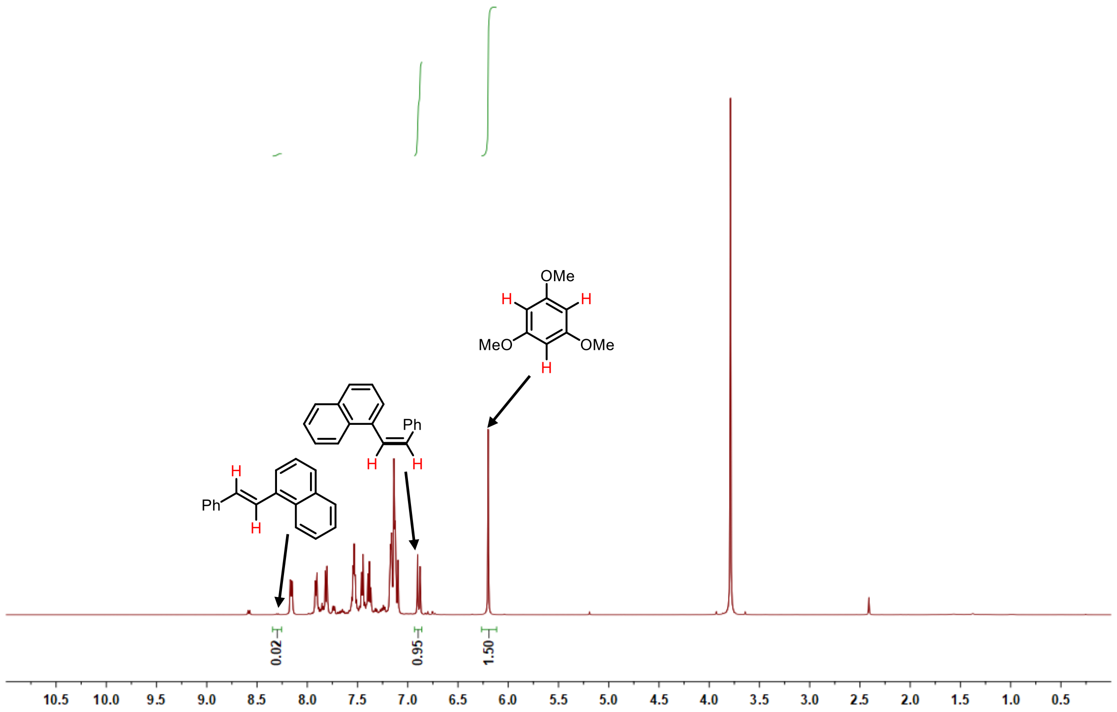

Figure S70. ^{1}H NMR ($CDCl_3$, 500 MHz) spectrum of the crude reaction mixture of hydrogenation of **1n** with thiol (Z/E = >20:1).

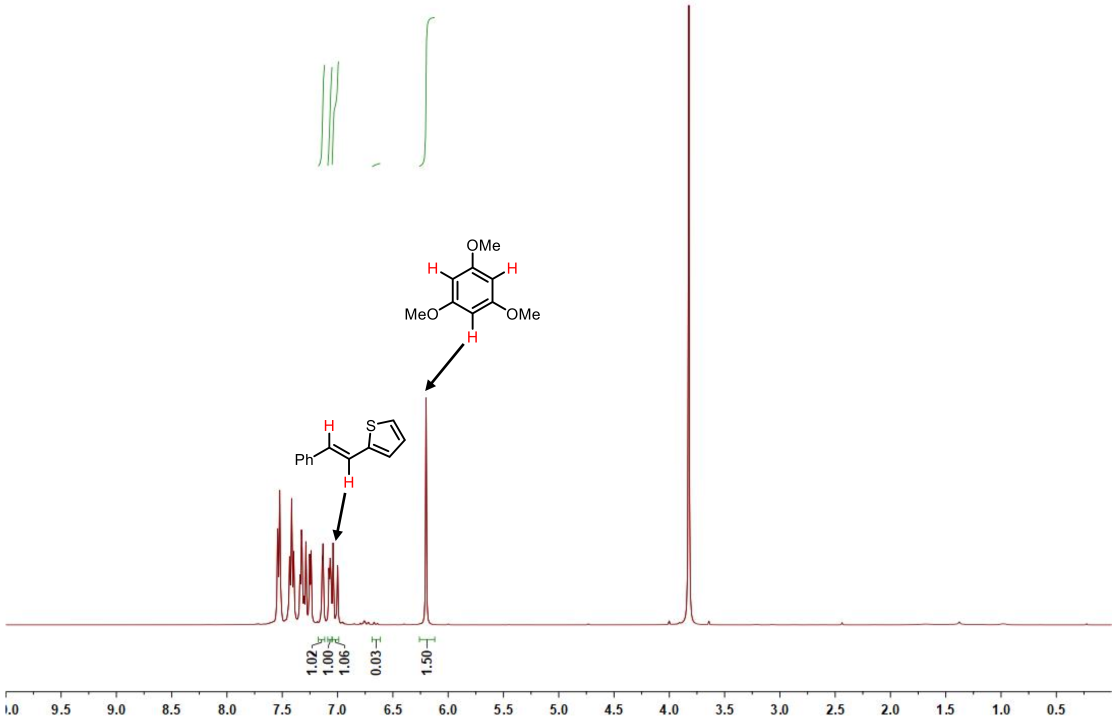

Figure S71. ^{1}H NMR ($CDCl_3$, 400 MHz) spectrum of the crude reaction mixture of hydrogenation of **1o** without thiol (E/Z = 36:1).

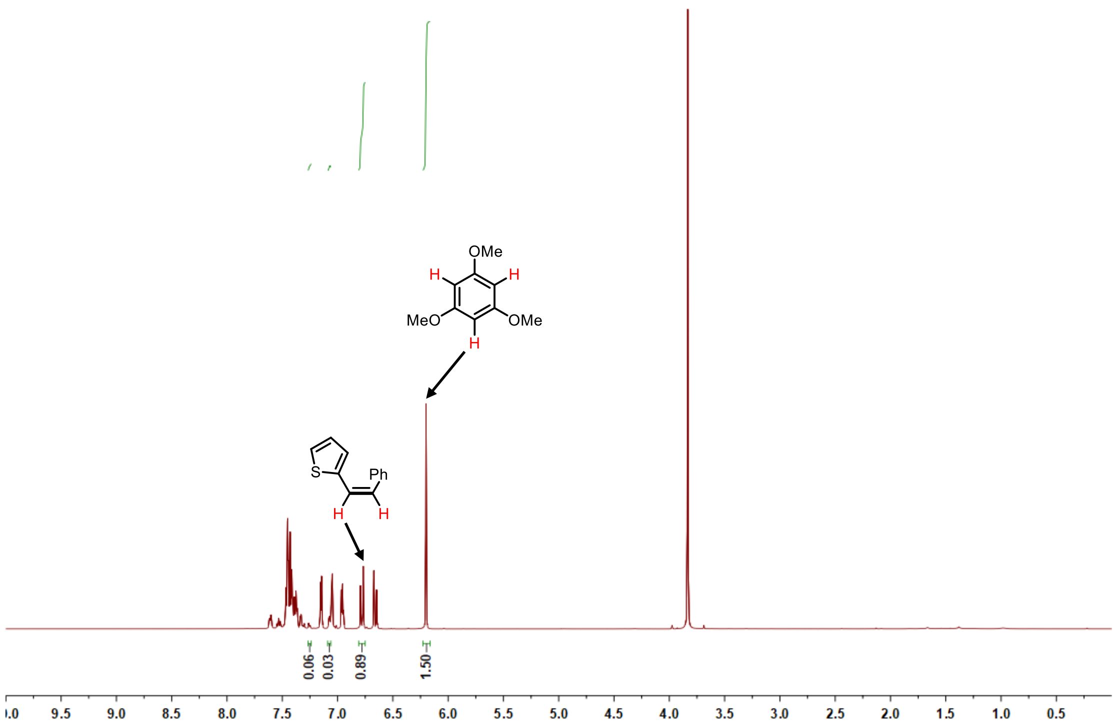

Figure S72. ^{1}H NMR ($CDCl_3$, 400 MHz) spectrum of the crude reaction mixture of hydrogenation of **1o** with thiol (Z/E = >20:1).

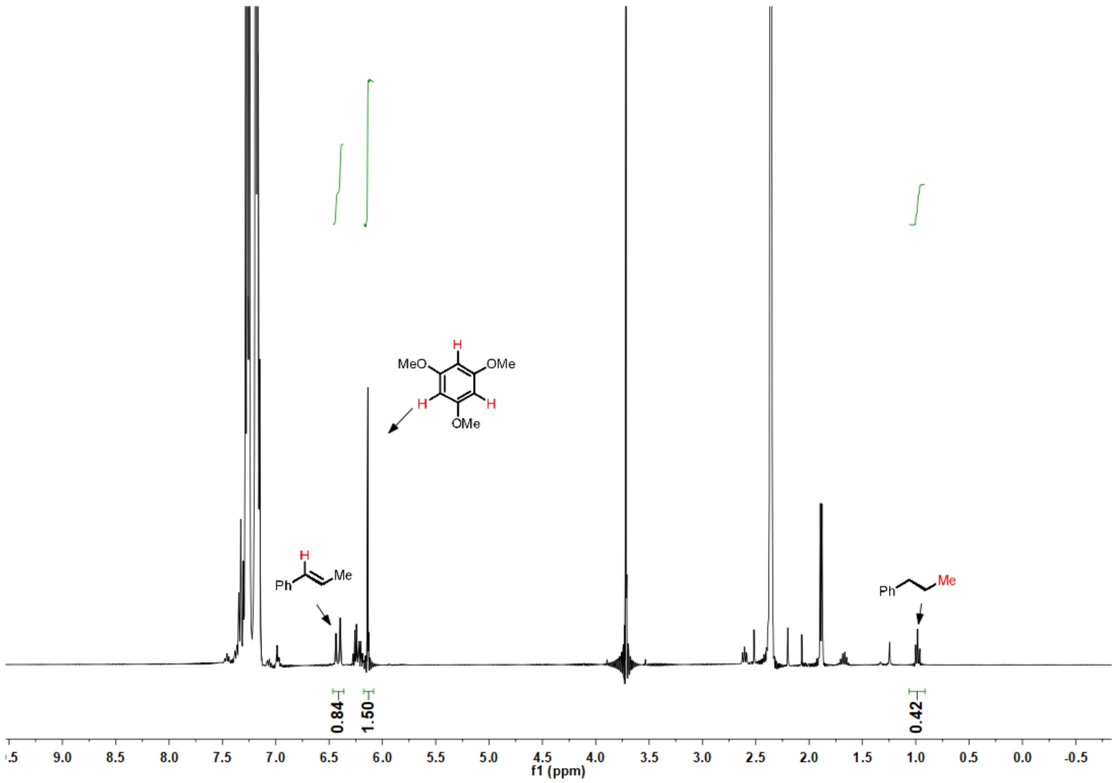

Figure S73. ^{1}H NMR ($CDCl_3$, 400 MHz) spectrum of the crude reaction mixture of hydrogenation of **1p** without thiol (E/Z = >99:1, 14% **3p**).

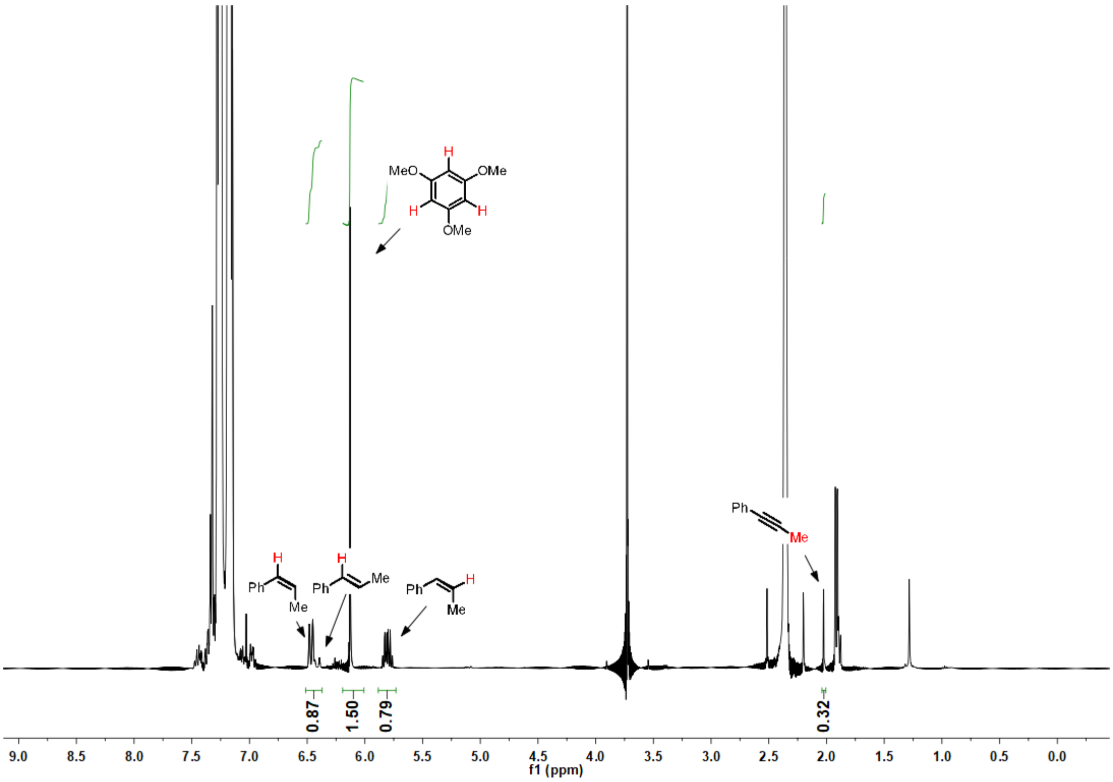

Figure S74. ^{1}H NMR ($CDCl_3$, 400 MHz) spectrum of the crude reaction mixture of hydrogenation of **1p** with thiol (Z/E = 10:1).

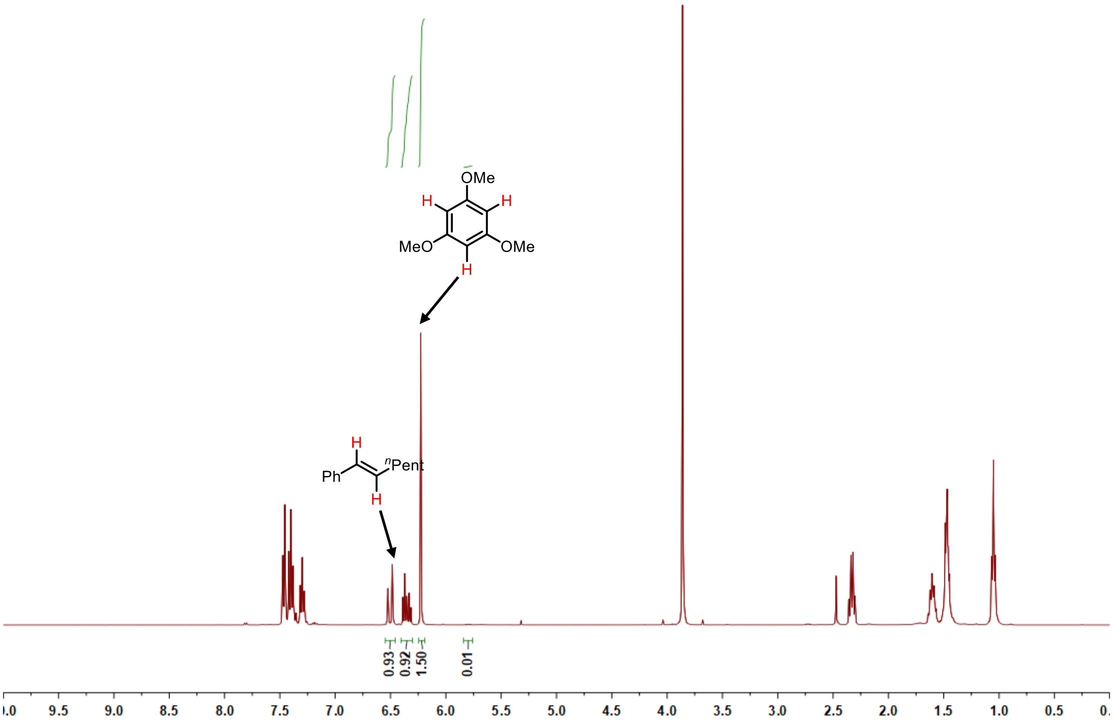

Figure S75. ^{1}H NMR ($CDCl_3$, 400 MHz) spectrum of the crude reaction mixture of hydrogenation of **1q** without thiol (E/Z = 92:1).

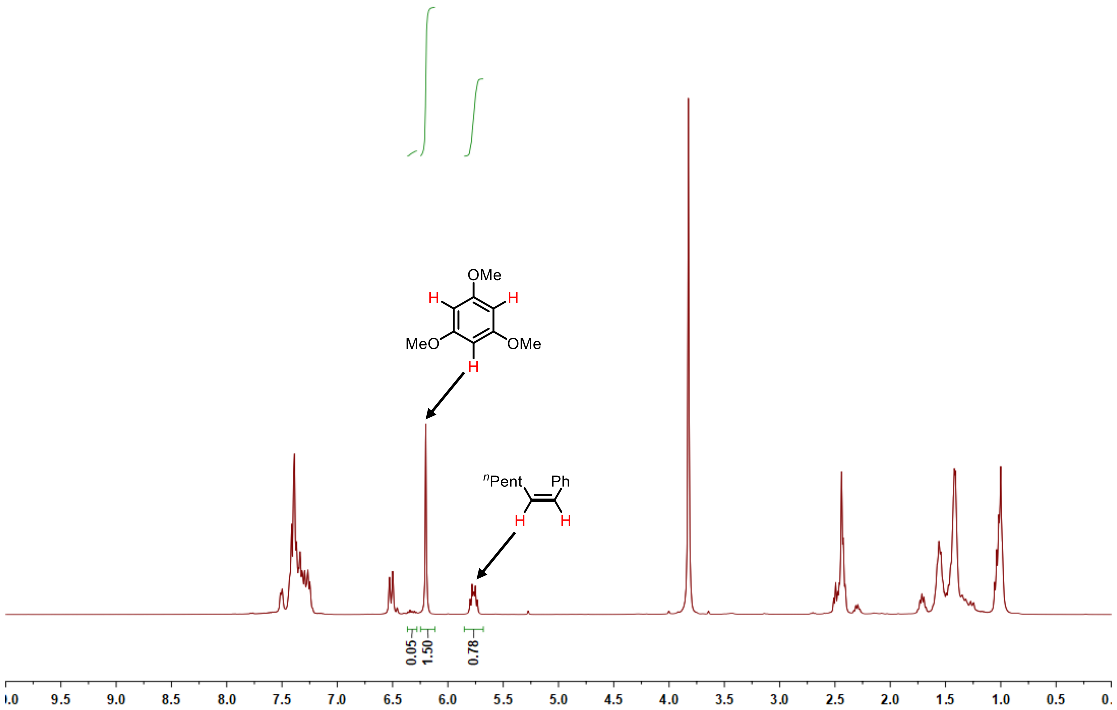

Figure S76. ^{1}H NMR ($CDCl_3$, 400 MHz) spectrum of the crude reaction mixture of hydrogenation of **1q** with thiol (Z/E = 16:1).

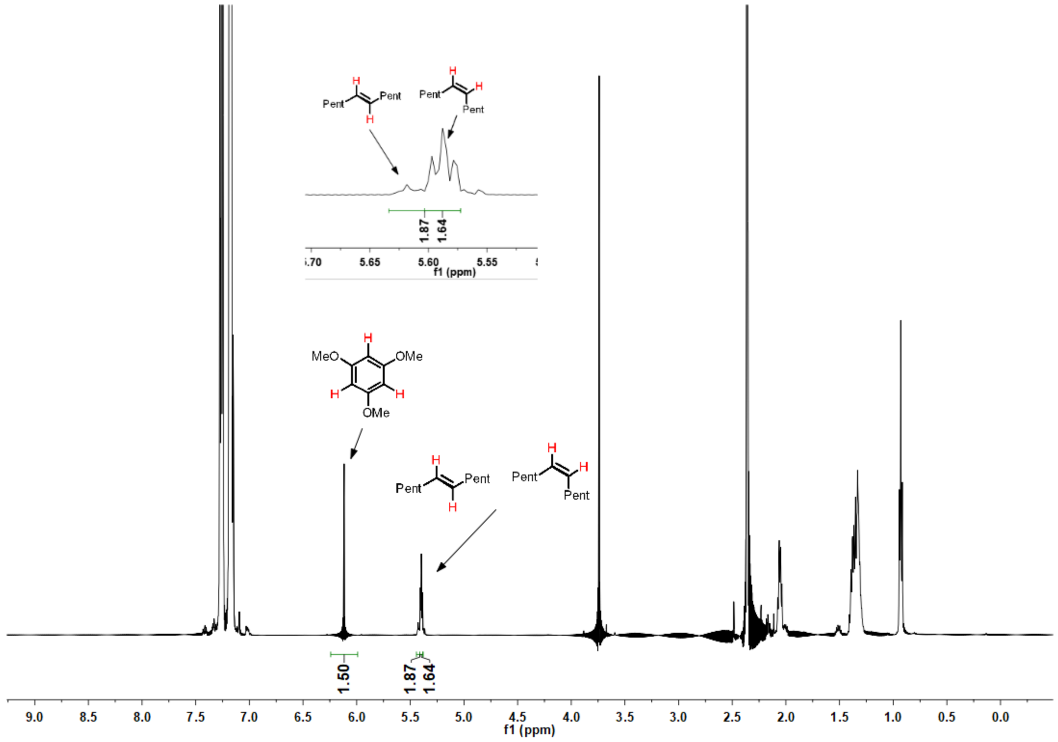

Figure S77. ^{1}H NMR ($CDCl_3$, 400 MHz) spectrum of the crude reaction mixture of hydrogenation of **1r** with thiol (Z/E = 7:1, with minor side product of double bond shifts).

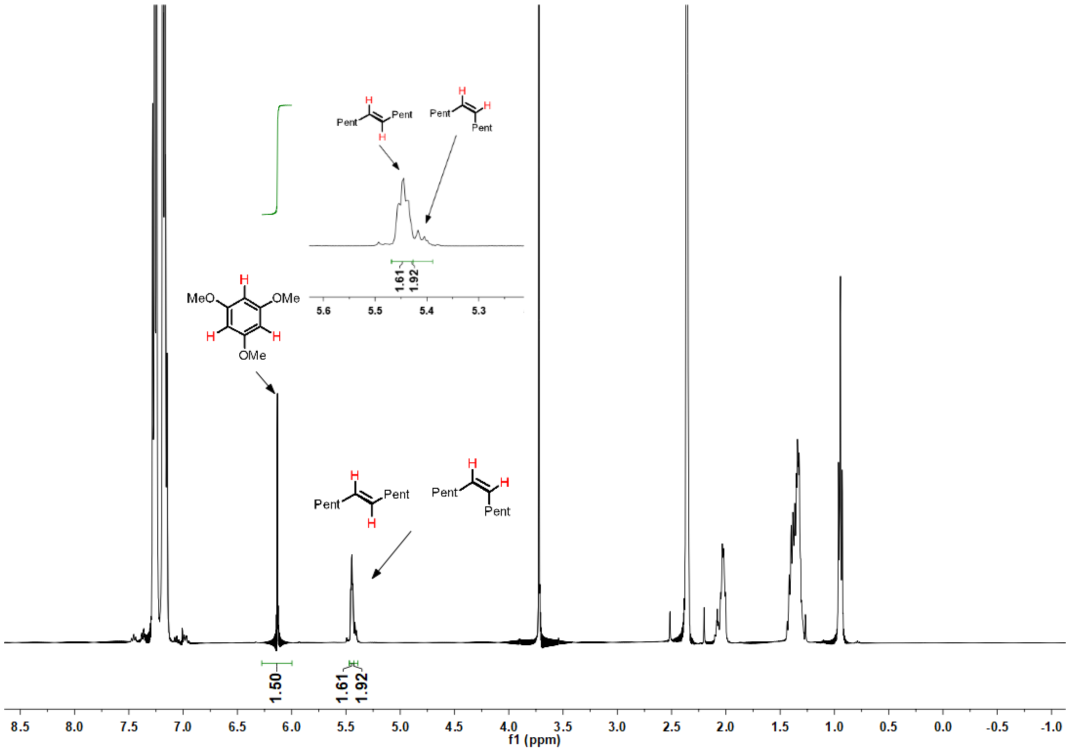

Figure S78. ^{1}H NMR ($CDCl_3$, 400 MHz) spectrum of the crude reaction mixture of hydrogenation of **1r** without thiol (E/Z = 5:1, with minor side product of double bond shifts).

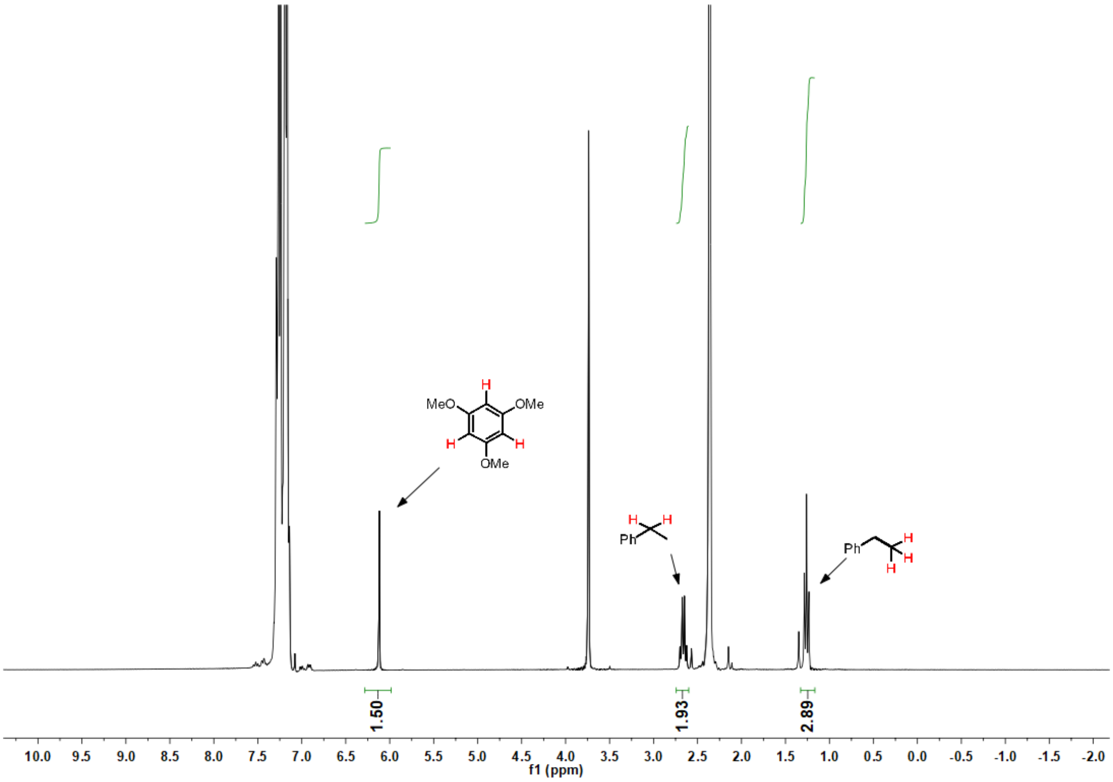

Figure S79. ^{1}H NMR ($CDCl_3$, 400 MHz) spectrum of the crude reaction mixture of hydrogenation of **1s** without thiol (**3s/2s =** >99:1).

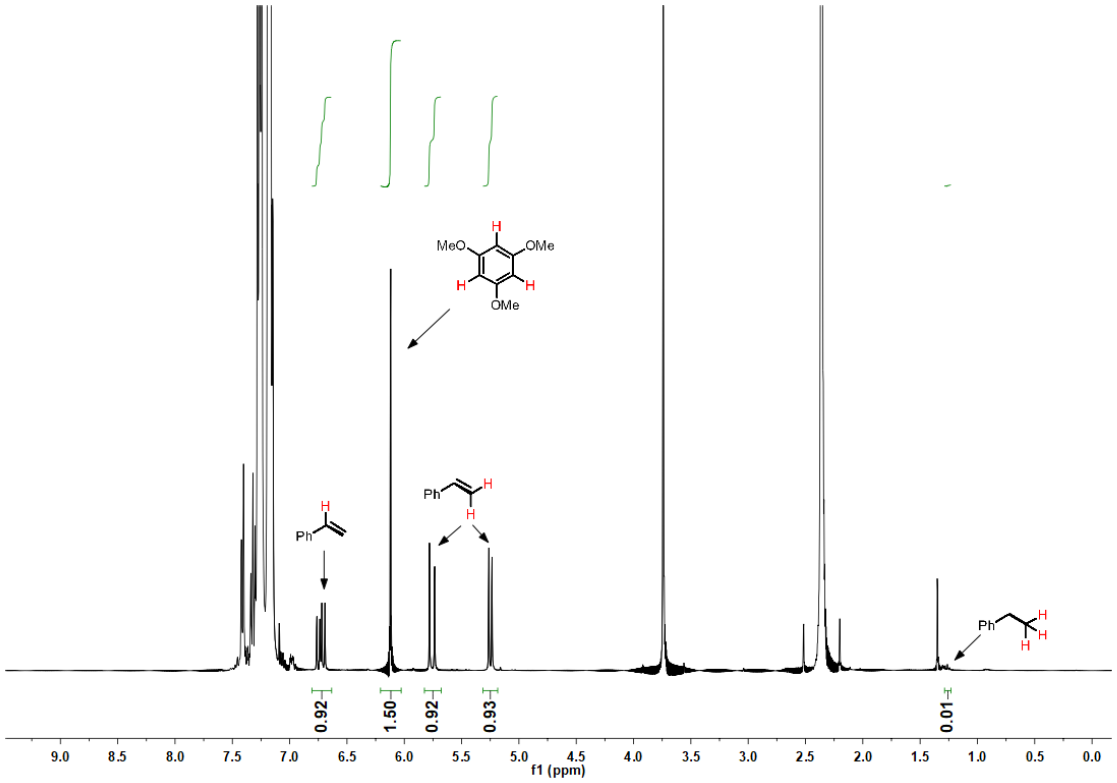

Figure S80. ^{1}H NMR ($CDCl_3$, 400 MHz) spectrum of the crude reaction mixture of hydrogenation of **1s** with thiol (**2s**/**3s** = 99:1).

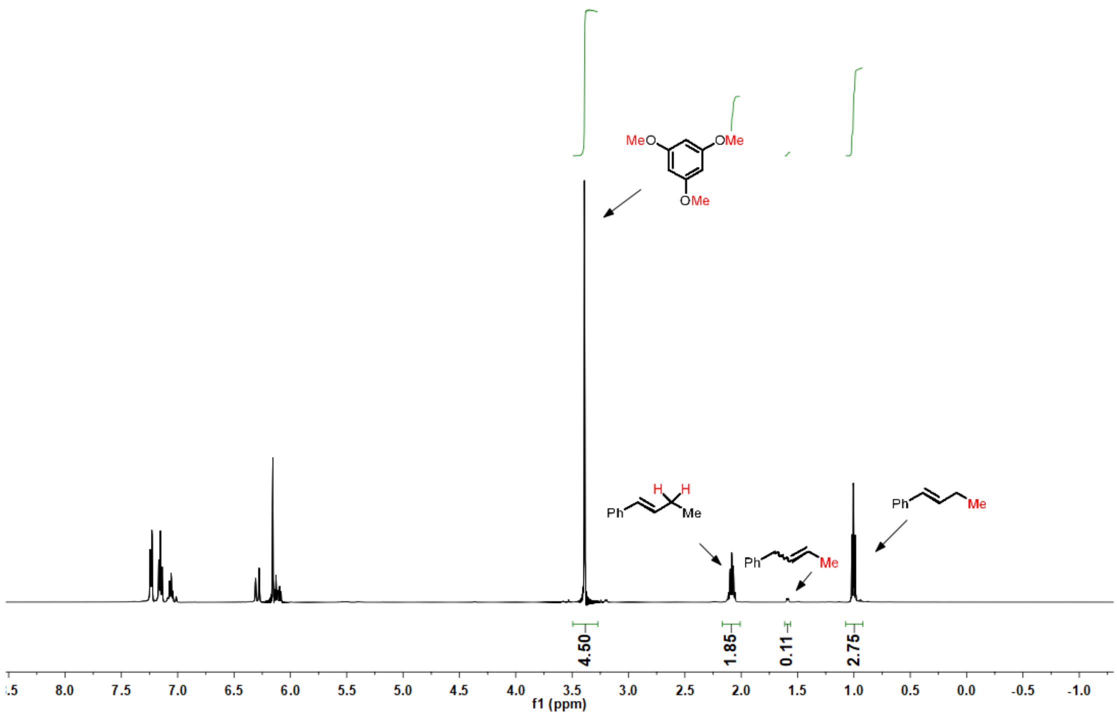

Figure S81. ^{1}H NMR (toluene-d_8, 500 MHz) spectrum of the crude reaction mixture of isomerization of **1-2t** (3-position/2-position > 20:1, E/Z = >99:1).

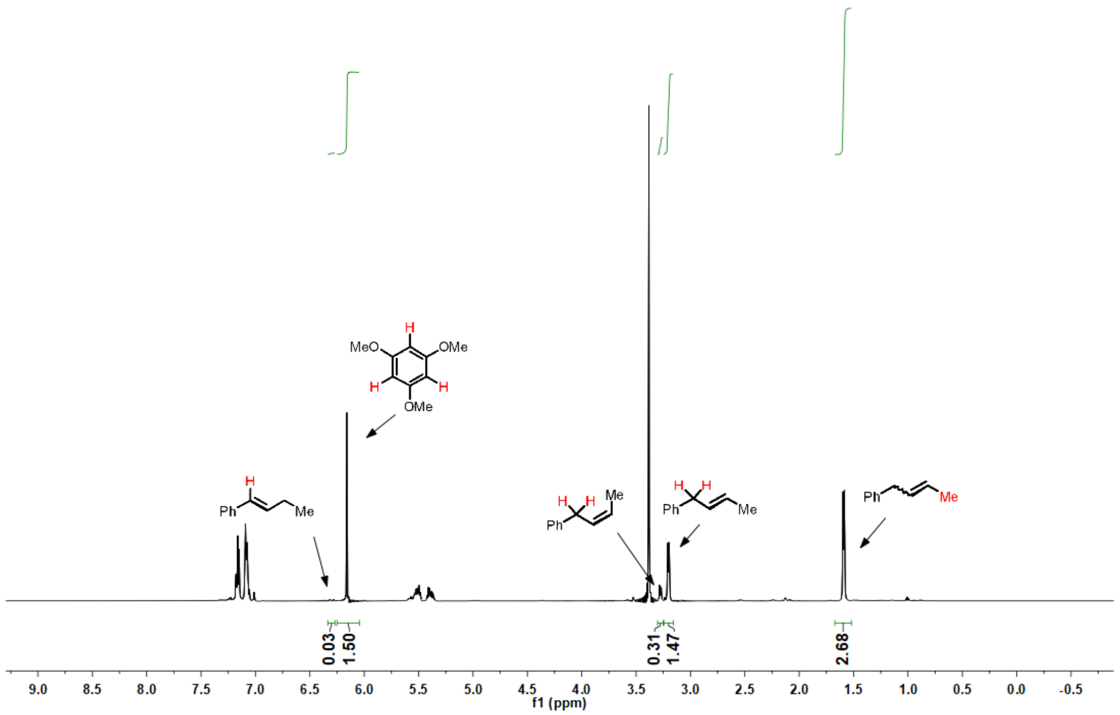

Figure S82. ^{1}H NMR (toluene-d_8, 500 MHz) spectrum of the crude reaction mixture of isomerization of **1-2t** with $HexNH_2$ (2-position/3-position > 20:1, E/Z = 5:1).

6. Characterization of substrate 1l

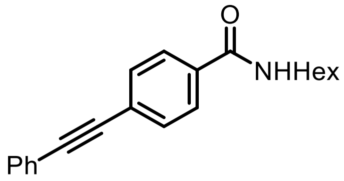

1l: IR (cm^{-1}): 3341, 2916, 1629, 1534 (alkyne peak hasn't been observed). ^{1}H NMR (500 MHz, $CDCl_3$) δ 7.74 (d, J = 8.2 Hz, 2H), 7.57 (d, J = 8.2 Hz, 2H), 7.55 – 7.52 (m, 2H), 7.38 – 7.34 (m, 3H), 6.24 (s, 1H), 3.49 – 3.34 (m, 2H), 1.65 – 1.54 (m, 2H), 1.44 – 1.24 (m, 6H), 0.90 (t, J = 6.6 Hz, 3H). ^{13}C NMR (126 MHz, $CDCl_3$) δ 166.94, 134.28, 131.80, 131.77, 128.77, 128.53, 127.02, 126.48, 122.90, 91.72, 88.72, 40.33, 31.63, 29.74, 26.81, 22.69, 14.15. GC-EI-MS m/z calcd. for $C_{21}H_{23}NO_2$ $[M]^+$: 305.2, found: 305.1.

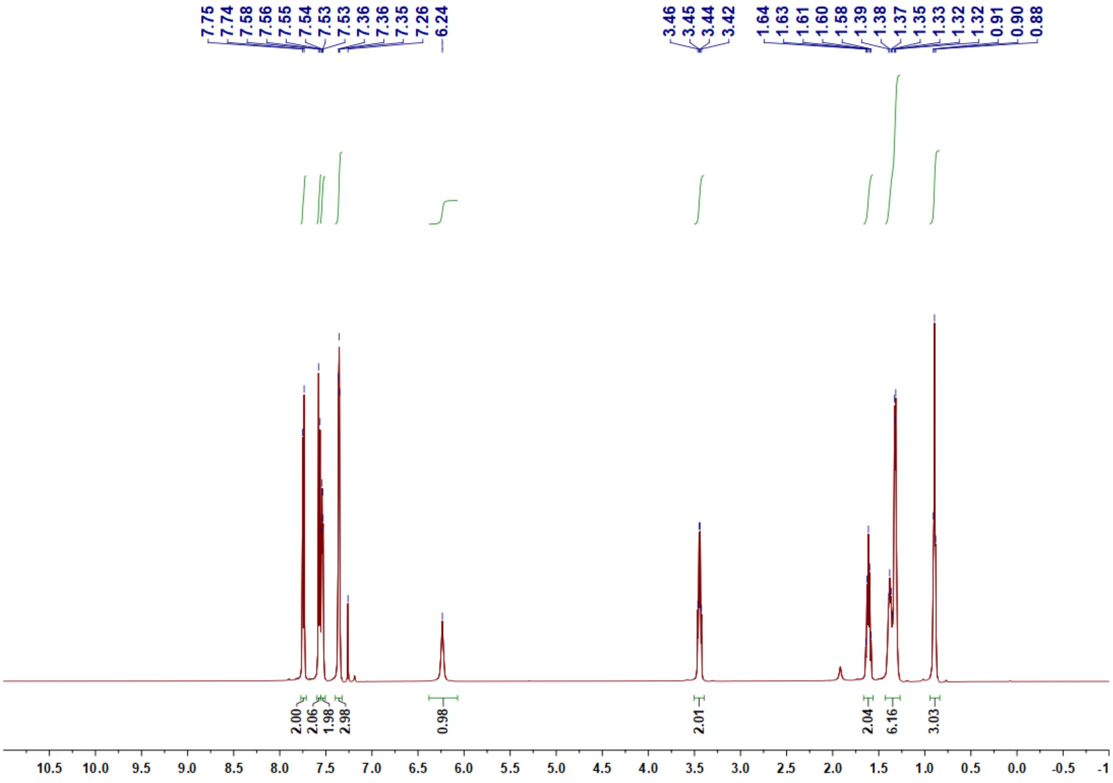

Figure S83. ^{1}H NMR (500 MHz, $CDCl_3$) spectrum of **1l**.

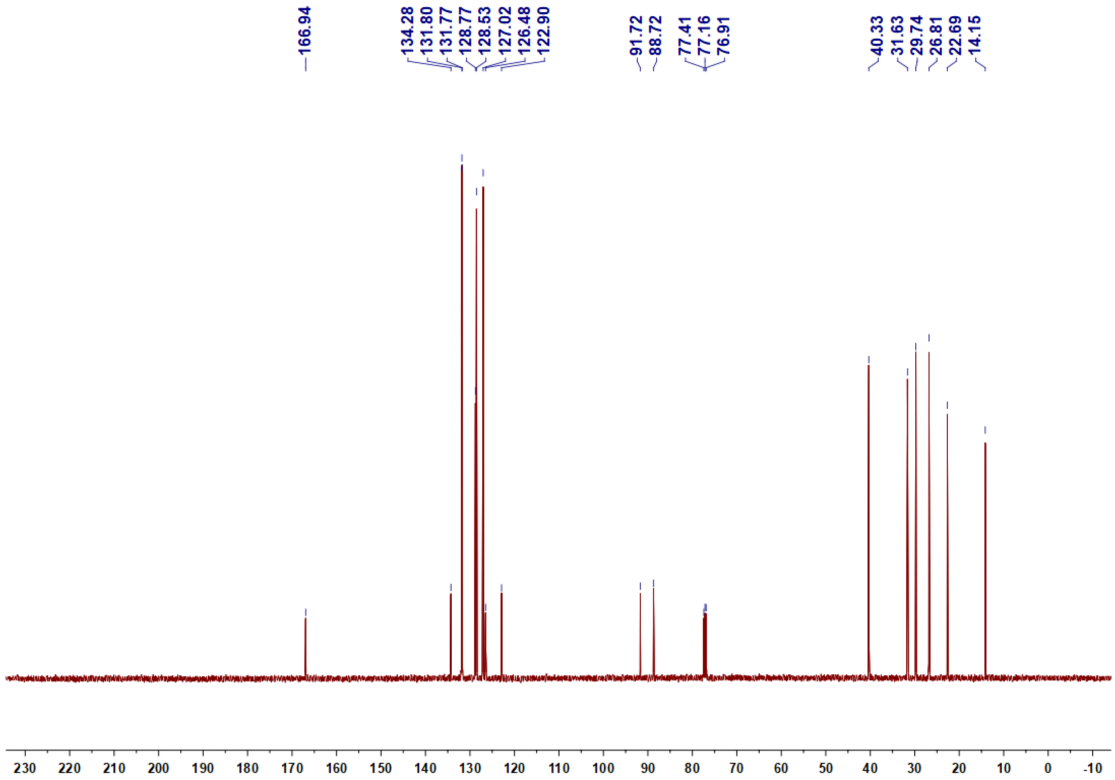

Figure S84. ^{13}C NMR (126 MHz, $CDCl_3$) spectrum of **1l**.

7. Single crystal X-ray diffraction analysis

Single crystal XRD were measured by a sealed tube Rigaku Synergy-S dual source equipped with Dectris Pilatus3 R CdTe 300K detector and microfocus diffractometer, with MoKα (λ=0.71073 Å). Data collection was performed in low temperature under LN. Data were processed with CrysAlis^{PRO} (Rigaku). Structure was solved using SHELXT [7] and refinement performed based on F[8] with SHELXL[8] and OLEX2[9] with full matrix least-squares. All non-hydrogen atoms were refined aniostropically. Hydrogens were placed at calculated positions and refined using a riding model.

Crystal data: $C_{35}H_{53}NO_3P_2RuS$, brown chunk, 0.16 x 0.15 x 0.10 mm^3, Triclinic *P-1*, a=7.8146(2)Å, b=13.3277(3)Å, c=16.5286(4)Å, α=88.939(2)°, β=88.230(2)°, γ=86.356(2)°, from 37889 reflections and 27° data, T=100(2)K, V=1716.93(7)Å^3, Z=2, Fw=730.85, Dc=1.414 Mg·m^{-3}, μ=0.646 mm^{-1}. Data completeness 1.00, 7874 independent reflections (R-int =0.0557). The data were processed with CrysAlis ^{PRO}. Full matrix least-squares refinement based on F^2 on 407 parameters with no restraints gave final R_1= 0.0408 (based on F^2) wR_2= 0.1029 for data with I>2σ(I) and, R_1= 0.0513 wR_2= 0.1081 for all data on 7874 reflections, goodness-of-fit on F^2 = 1.038 largest electron density peak 1.952 e·Å^{-3}. Largest hole –0.539 e·Å^{-3}.

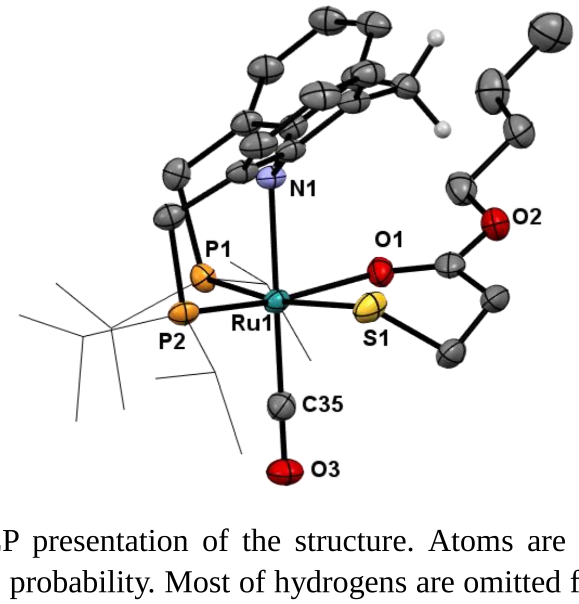

Figure S85. ORTEP presentation of the structure. Atoms are presented as thermal ellipsoids with 50% probability. Most of hydrogens are omitted for clarity. Colors are: Carbon-Grey, Oxygen-Red, Nitrogen-Purple, Phosphur-Orange, Sulphur-Yellow, Ruthenium-Green, Hydrogen-White.

8. Computational details

DFT calculations were performed with Gaussian 16 (C.01 revision)[10] using Truhlar's M06-L functional,[11] the triple-ξ def2-TZVP basis set,[12] W06 density fitting,[13] and Grimme's D3(0) empirical dispersion correction.[14] Frequency calculations at this level of theory were run to confirm stationary points and transition states and to obtain thermodynamic corrections. Single point energies of the M06-L optimized structures were computed with ORCA (4.2.1)[15] using the range-separated meta-GGA hybrid functional ωB97M-V of the Head-Gordon group[16] including dispersion correction,[17,18] together with the triple-ξ def2-TZVPP basis set[12] and the corresponding auxiliary basis sets, def2/J[13] and def2-TZVPP/C[19] for RIJCOSX density fitting. The functional and basis set selections are based on recent benchmark studies.[20] The polarizable continuum model (IEFPCM) was used in all calculations (optimization and single point) with the SMD solvation (Toluene) model of Truhlar and co-workers.[21]

Gibbs free energies were computed by adding the free energy correction term from the frequency calculation to the single point energy in methanol, according to

$$\mathbf{G}^{\omega\mathrm{B97M\text{-}V}}_{\mathrm{(Toluene)}} = \mathbf{E}^{\omega\mathrm{B97M\text{-}V}}_{\mathrm{Toluene}} + \mathbf{corr}^{\mathit{M06\text{-}L}}_{\mathit{freq}\mathrm{(Toluene)}}$$

where $\mathbf{E}^{\omega\mathrm{B97M\text{-}V}}_{\mathrm{Toluene}}$ is the single point energy; and
where $\mathbf{corr}^{\mathit{M06\text{-}L}}_{\mathit{freq}}$ is the thermal correction to the Gibbs free energy from the frequency calculation.

Free energy values (G°) were then corrected to account for changes in standard states ($G^{\circ} \rightarrow G$).
Standard state corrections[22] were employed such that all species are treated as 1M (using an ideal gas approximation), with the exception of H_2 (maintained as 1 atm).[23-25] Other than these standard state corrections, the transformation of hydrogen from the condensed phase to the gas phase is not additionally corrected in the free energy quantities provided.

Ethanethiol were studied as minimal models for hexanethiol in the system. Directionality of ΔG and ΔG_{TS} values are indicated by the ordering of X,Y and all energies are reported in kcal/mol. Mass balance is ensured throughout.

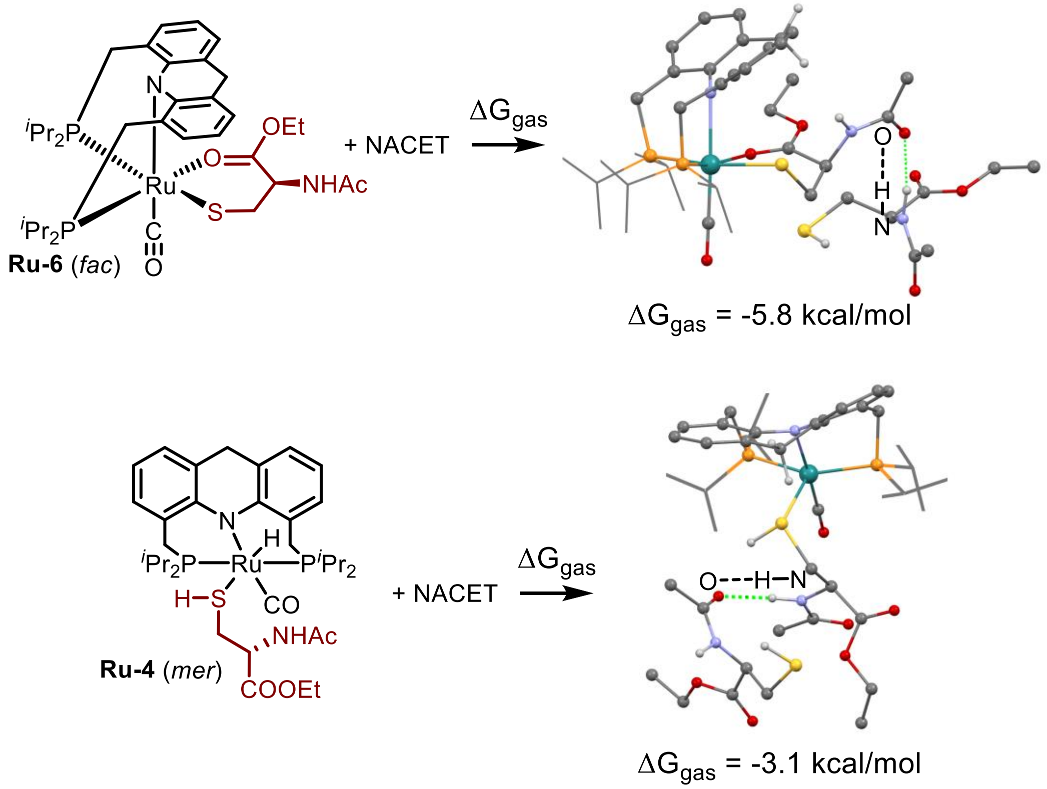

Figure S86. Two examples of hydrogen bonding interactions between NACET units modeled in gas phase.

Table S2 Energy data

**Structure**	$E^{\omega B97M\text{-}V}_{Toluene}$	$G^{o\omega B97M\text{-}V}_{Toluene}$	**G**
Energy Unit	Hartree	Hartree	kcal
***mer* Ru-1**	-1999.289765	-1998.722741	-1254196.625
**Ru-4 (EtSH)**	-2477.302498	-2476.663482	-1554104.44
**Ru-5 (EtSH)**	-2476.113352	-2475.488188	-1553366.943

**Ru-4 (NACET)**	-2952.567542	-2951.814419	-1852261.653
**Ru-4 (NACET) (gas)**	-2952.528493	-2951.77607	-1852237.589
**Ru-4-NACET (gas)**	-3905.790961	-3904.850405	-2450291.734
**Ru-6**	-2951.401536	-2950.659179	-1851536.74
**Ru-6 (gas)**	-2951.366388	-2950.623313	-1851514.234
**Ru-6-NACET (gas)**	-3904.62414	-3903.702053	-2449571.143
**EtSH**	-477.9865194	-477.9375854	-299903.9397
**NACET**	-953.2485026	-953.0907816	-598062.5703
**NACET (gas)**	-953.2303572	-953.0733042	-598051.0629
**hydrogen**	-1.161074769	-1.162683769	-729.5840651

Cartesian Coordinates

mer Ru-1

Ru -0.2201830000 6.3497940000 1.3432180000
P -1.8352660000 7.5319770000 0.1687840000
P 1.6887590000 5.9843590000 2.6252470000
O -2.1618920000 5.2090050000 3.2863310000
N 0.9521750000 6.1152860000 -0.4786570000
C -1.3932300000 5.7053580000 2.5611860000
C -1.6310820000 10.1078330000 1.2740050000
C -3.2429510000 8.5346250000 2.3665840000
C -2.6103540000 8.9662010000 1.0528440000
C -4.0572200000 7.4665030000 -1.6150700000
C -2.9759410000 5.2694140000 -1.0425850000
C -3.3114940000 6.6724480000 -0.5551040000
C 3.5286010000 4.2131480000 3.8894790000
C 1.1939950000 3.3516050000 3.4636620000
C 2.3076010000 4.2728600000 2.9878170000
C 0.7057780000 6.5950260000 5.1761370000
C 1.9271180000 8.4273760000 3.9921240000
C 1.8366640000 6.9258320000 4.2157200000
C 0.3580290000 6.1794410000 -1.7571620000
C 2.2012880000 5.4583820000 -0.4777370000

C -0.8973810000 8.3142630000 -1.1985770000
C -0.4568950000 7.2625530000 -2.1594870000
C -0.9368070000 7.3174090000 -3.4649920000
C -0.6688580000 6.3261630000 -4.3925290000
C 0.0766940000 5.2317310000 -3.9880840000
C 0.5690480000 5.1489860000 -2.6962640000
C 1.3117460000 3.9536310000 -2.2093600000
C 2.4635760000 4.4119080000 -1.3842970000
C 3.7143480000 3.8225710000 -1.4693500000
C 4.7432130000 4.2220670000 -0.6330270000
C 4.4814300000 5.1979210000 0.3128970000
C 3.2381910000 5.8153810000 0.4151160000
C 2.9948270000 6.7301240000 1.5644900000
H -0.7620780000 9.7808320000 1.8480340000
H -1.2717170000 10.5412250000 0.3421040000
H -2.1110340000 10.9073110000 1.8403790000
H -3.8200170000 9.3550960000 2.7956130000
H -3.9174260000 7.6854190000 2.2567890000
H -2.4784480000 8.2550100000 3.0928710000
H -3.4010270000 9.3204650000 0.3832180000
H -4.9860590000 6.9604040000 -1.8835700000
H -4.3230990000 8.4716550000 -1.2887220000
H -3.4659570000 7.5540440000 -2.5267890000
H -2.4031900000 5.2927610000 -1.9696600000
H -2.3966100000 4.7110140000 -0.3048370000
H -3.8911650000 4.7090740000 -1.2398770000
H -3.9584200000 6.5702650000 0.3249830000
H 3.9562770000 3.2092370000 3.8809610000
H 4.3146880000 4.9006620000 3.5749350000
H 3.2777460000 4.4432980000 4.9261960000
H 0.8236650000 3.6201800000 4.4534120000
H 0.3448500000 3.3616320000 2.7803020000
H 1.5569210000 2.3245720000 3.5260450000
H 2.6037710000 3.9321980000 1.9901650000
H -0.2493080000 6.9525920000 4.7886330000
H 0.6043360000 5.5269660000 5.3639020000
H 0.8713020000 7.0821890000 6.1383980000
H 2.0107690000 8.9391960000 4.9521190000
H 2.7911250000 8.7148080000 3.3951550000
H 1.0342350000 8.8128370000 3.4974400000
H 2.7839670000 6.5947420000 4.6527110000
H -0.0494700000 8.8050520000 -0.7110590000
H -1.4826410000 9.0831680000 -1.7077640000
H -1.5378060000 8.1740210000 -3.7498240000

H -1.0464000000 6.4002170000 -5.4037440000
H 0.2769390000 4.4173790000 -4.6756080000
H 0.6392450000 3.3496820000 -1.5794940000
H 1.6287050000 3.3098030000 -3.0297400000
H 3.8699390000 3.0312020000 -2.1942500000
H 5.7229410000 3.7679120000 -0.7002100000
H 5.2581230000 5.4992850000 1.0072860000
H 3.9191070000 6.9136600000 2.1166550000
H 2.5913320000 7.6975030000 1.2520580000
H -0.2515290000 7.6516030000 2.2301470000

Ru-4 (EtSH)

Ru -0.3731770000 6.1247230000 1.2604510000
P -1.8794070000 7.4965670000 0.0991880000
P 1.4890070000 5.9359140000 2.6144710000
O -2.1130470000 5.4131520000 3.5154010000
N 0.9652740000 6.0945420000 -0.6395900000
C -1.4673300000 5.8581850000 2.6348750000
C -1.4885320000 10.1540450000 1.1744980000
C -2.8366050000 8.4966200000 2.4646440000
C -2.4453820000 8.9696990000 1.0730270000
C -3.9440680000 7.9467580000 -1.7992880000
C -3.4925710000 5.5548810000 -1.1447310000
C -3.4907920000 7.0146440000 -0.6802120000
C 3.4843180000 4.5466480000 3.9852350000
C 1.2691600000 3.3324260000 3.6734430000
C 2.2636560000 4.3438620000 3.1241770000
C 0.5329650000 6.4167820000 5.2520670000
C 1.5157840000 8.3683180000 4.0179580000
C 1.5748620000 6.8502220000 4.2109560000
C 0.3327990000 6.1181680000 -1.8804760000
C 2.1645800000 5.3823640000 -0.5176960000
C -0.8951740000 8.2205550000 -1.2669530000
C -0.5451590000 7.1698620000 -2.2599310000
C -1.0797820000 7.2286140000 -3.5443720000
C -0.7931490000 6.2595060000 -4.4974370000
C 0.0342210000 5.1951840000 -4.1160270000
C 0.5701030000 5.1152300000 -2.8263500000
C 1.3930270000 3.9327970000 -2.3828560000
C 2.4775290000 4.3579820000 -1.4260790000
C 3.7214280000 3.7549490000 -1.4323430000
C 4.7105480000 4.1344740000 -0.5231030000
C 4.3965340000 5.1086030000 0.4190210000
C 3.1372600000 5.7255290000 0.4554370000

C 2.7912510000 6.7071650000 1.5542570000
H -0.5272680000 9.8694210000 1.5873240000
H -1.3017130000 10.6303630000 0.2223360000
H -1.9128790000 10.9151190000 1.8348650000
H -3.3768480000 9.2620290000 3.0273110000
H -3.4639450000 7.6058970000 2.4608880000
H -1.8982950000 8.2465130000 3.0456060000
H -3.3252090000 9.3382830000 0.5397090000
H -4.9725880000 7.7119940000 -2.0691680000
H -3.9196400000 9.0049680000 -1.5352650000
H -3.3266480000 7.8141890000 -2.6886010000
H -2.7268470000 5.3624790000 -1.9056100000
H -3.3075420000 4.8188270000 -0.3124770000
H -4.4597080000 5.3008720000 -1.5750840000
H -4.1900750000 7.1101810000 0.1583890000
H 4.0154130000 3.5973360000 4.0755740000
H 4.1842100000 5.2737270000 3.5894230000
H 3.2384790000 4.8707250000 4.9967910000
H 1.0997110000 3.4693990000 4.7339870000
H 0.2763290000 3.3343320000 3.1533010000
H 1.6821200000 2.3373170000 3.5588450000
H 2.5722830000 3.9447450000 2.1912050000
H -0.5041210000 6.7210170000 4.9448720000
H 0.5098260000 5.3501090000 5.4458400000
H 0.7701520000 6.9108250000 6.1935790000
H 1.7049740000 8.9003270000 4.9571440000
H 2.2373780000 8.7298160000 3.2896890000
H 0.5184340000 8.6510090000 3.6838160000
H 2.5785080000 6.5755370000 4.5838090000
H -0.0018680000 8.6340000000 -0.7952410000
H -1.4462650000 9.0445270000 -1.7317940000
H -1.7177730000 8.0702660000 -3.8024390000
H -1.2022060000 6.3288640000 -5.4980050000
H 0.2549740000 4.3992810000 -4.8188310000
H 0.7327870000 3.2029500000 -1.8789140000
H 1.8100950000 3.4055320000 -3.2456670000
H 3.9214110000 2.9798090000 -2.1662310000
H 5.6885180000 3.6706890000 -0.5426070000
H 5.1482250000 5.4182150000 1.1349530000
H 3.6760910000 6.9721930000 2.1478790000
H 2.3707010000 7.6289620000 1.1476590000
H -0.0287540000 7.6156920000 1.8049930000
S -0.5347640000 3.8468880000 0.1904910000
H 0.6428730000 3.3191980000 0.5236450000

C -1.6220000000 2.7440600000 1.1319950000
H -2.6136270000 3.0416930000 0.8563800000
H -1.5236670000 2.9867370000 2.2066710000
C -1.3755640000 1.2608920000 0.8246220000
H -2.0599620000 0.6456610000 1.3927820000
H -1.5153250000 1.0179480000 -0.2261920000
H -0.3536170000 0.9651850000 1.0989530000

Ru-5
(EtSH)

S -0.1178080000 10.9723980000 3.6735910000
P -2.0878360000 7.9448630000 2.9301480000
P 0.6914210000 6.6784830000 4.8510950000
O -1.8330740000 8.9888880000 6.6887230000
N 0.8848610000 8.4001100000 2.2631220000
C -1.5649980000 7.7844450000 1.1768110000
H -0.9559490000 6.8747450000 1.1503630000
H -2.4531430000 7.6071370000 0.5697420000
C -0.7833600000 8.9340250000 0.6403430000
C -1.2409640000 9.7149410000 -0.4124860000
H -2.2284290000 9.5274660000 -0.8218420000
C -0.4316810000 10.6952850000 -0.9695610000
H -0.7907040000 11.2930140000 -1.7971270000
C 0.8492340000 10.8903510000 -0.4678820000
H 1.4907830000 11.6484420000 -0.9046660000
C 1.3166810000 10.1516820000 0.6078600000
C 0.4894930000 9.1773850000 1.1871070000
C 2.6521250000 10.3840820000 1.2494910000
H 3.3662790000 10.8059930000 0.5393730000
H 2.5305760000 11.1411980000 2.0367080000
C 3.1851670000 9.1247800000 1.8663040000
C 4.5404940000 8.8549460000 1.9620100000
H 5.2475930000 9.5663620000 1.5484910000
C 5.0018390000 7.7057240000 2.5920540000
H 6.0636620000 7.5120790000 2.6696230000
C 4.0861370000 6.8009610000 3.1062530000
H 4.4326340000 5.8856900000 3.5746490000
C 2.7181730000 7.0235020000 2.9955380000
C 2.2514480000 8.2101170000 2.3932240000
C 1.7315760000 6.0141220000 3.4838860000
H 2.2431000000 5.1065970000 3.8053840000
H 1.0320190000 5.7425260000 2.6860980000
C -2.8294720000 6.2817050000 3.2823580000
H -1.9167530000 5.6768310000 3.3246120000

C -3.7086870000 5.6541260000 2.2080510000
H -4.6818610000 6.1322800000 2.1306810000
H -3.8890930000 4.6088750000 2.4658130000
H -3.2471200000 5.6628110000 1.2226280000
C -3.5046800000 6.2406760000 4.6438230000
H -2.8994910000 6.6995140000 5.4257940000
H -3.7065460000 5.2105250000 4.9431920000
H -4.4635970000 6.7598670000 4.6218240000
C -3.4224370000 9.2315780000 2.8012630000
H -2.8820040000 10.0080200000 2.2452650000
C -3.8637250000 9.8324710000 4.1233900000
H -4.3017280000 9.0918740000 4.7931420000
H -4.6237590000 10.5942670000 3.9435640000
H -3.0412900000 10.3124460000 4.6442430000
C -4.6360600000 8.8106890000 1.9835180000
H -4.3861630000 8.3174380000 1.0456550000
H -5.2223790000 9.6960860000 1.7332830000
H -5.2910900000 8.1491290000 2.5508570000
C 2.0166190000 7.1857090000 6.0641650000
H 2.6587190000 7.7852950000 5.4084130000
C 1.5499570000 8.1027450000 7.1836700000
H 1.1180260000 9.0244850000 6.7969410000
H 2.4029440000 8.3847250000 7.8031180000
H 0.8147910000 7.6382830000 7.8397010000
C 2.8628610000 6.0414300000 6.6034910000
H 2.3226340000 5.4475240000 7.3411970000
H 3.7447850000 6.4450590000 7.1040280000
H 3.2177360000 5.3690380000 5.8234900000
C -0.2077150000 5.2093090000 5.5615930000
H -1.0963430000 5.1448770000 4.9319530000
C -0.6881400000 5.4792520000 6.9804810000
H 0.1309900000 5.4405030000 7.6982810000
H -1.4129290000 4.7201900000 7.2795080000
H -1.1732630000 6.4496730000 7.0822720000
C 0.5039380000 3.8682720000 5.4523430000
H 0.7240390000 3.5983050000 4.4207200000
H -0.1393070000 3.0847750000 5.8574780000
H 1.4360790000 3.8372470000 6.0130500000
C -0.7529350000 12.0100640000 5.0370700000
H -0.8572480000 13.0146860000 4.6241970000
H -1.7579470000 11.6820650000 5.3043950000
C 0.1368040000 12.0326850000 6.2563530000
H 1.1469750000 12.3554410000 6.0048820000
H 0.2111040000 11.0443250000 6.7100420000

H -0.2550250000 12.7157100000 7.0131680000
C -1.2506290000 8.8682150000 5.6894960000
Ru -0.3116610000 8.6681610000 4.1262720000

Ru-4 (NACET)

Ru -0.1211030000 5.9115660000 1.3630690000
P -1.9081870000 6.9238830000 0.2205180000
P 1.8782380000 5.9583900000 2.5533650000
O -1.7350210000 5.4234360000 3.8208330000
N 1.0911760000 6.1496830000 -0.6132120000
C -1.0997830000 5.6253260000 2.8637790000
C -2.2730780000 8.8709080000 2.2172080000
C -4.1215890000 7.1932040000 1.9439860000
C -3.0354940000 7.9928030000 1.2383150000
C -4.1000070000 6.9340180000 -1.5990020000
C -2.5081070000 5.0147400000 -1.8113870000
C -3.1330690000 6.0245080000 -0.8555850000
C 3.9551940000 4.4972240000 3.8551750000
C 1.6447860000 3.5115460000 3.9373520000
C 2.6220220000 4.3621820000 3.1382690000
C 1.0040190000 6.8889200000 5.0682620000
C 2.0057170000 8.5884550000 3.5392790000
C 2.0365460000 7.1331550000 3.9801370000
C 0.4817960000 6.3948820000 -1.8430410000
C 2.2945910000 5.4420980000 -0.6950440000
C -1.0499280000 8.1523390000 -0.8520350000
C -0.4463210000 7.4521860000 -2.0203410000
C -0.9007680000 7.7782140000 -3.2939830000
C -0.5099150000 7.0732060000 -4.4197900000
C 0.3060310000 5.9659810000 -4.2428860000
C 0.7675590000 5.6110200000 -2.9870240000
C 1.5013110000 4.3323100000 -2.7600730000
C 2.5757130000 4.5455400000 -1.7505010000
C 3.7946760000 3.8929490000 -1.8383420000
C 4.7794690000 4.0799570000 -0.8813440000
C 4.5265070000 4.9604970000 0.1567100000
C 3.3233120000 5.6511760000 0.2553250000
C 3.1228010000 6.6057110000 1.3758860000
H -1.8379000000 8.2846580000 3.0264090000
H -1.4654480000 9.4259610000 1.7394850000
H -2.9512200000 9.5984000000 2.6665370000
H -4.7028280000 7.8475370000 2.5955930000
H -4.8205260000 6.7296980000 1.2492030000
H -3.7071690000 6.4025990000 2.5694550000

H -3.5111290000 8.6537560000 0.5057220000
H -4.8096270000 6.3366360000 -2.1744070000
H -4.6819710000 7.5741870000 -0.9377910000
H -3.5715100000 7.5710690000 -2.3098440000
H -2.1721290000 5.4859850000 -2.7339670000
H -1.6443030000 4.5059900000 -1.3832670000
H -3.2437900000 4.2546170000 -2.0819720000
H -3.6968800000 5.4578760000 -0.1049450000
H 4.4233370000 3.5184590000 3.9735820000
H 4.6613590000 5.1297030000 3.3173880000
H 3.8287960000 4.9117030000 4.8564410000
H 1.5531190000 3.8503140000 4.9691640000
H 0.6401920000 3.5158740000 3.5129780000
H 1.9860920000 2.4748250000 3.9779240000
H 2.8017000000 3.8586350000 2.1799030000
H 0.0122910000 7.1975950000 4.7391100000
H 0.9369230000 5.8466970000 5.3754570000
H 1.2472510000 7.4773910000 5.9543910000
H 2.0779690000 9.2385040000 4.4127220000
H 2.8267970000 8.8476040000 2.8735220000
H 1.0713250000 8.8315570000 3.0305090000
H 3.0330930000 6.9256830000 4.3839810000
H -0.3014020000 8.6263220000 -0.2131930000
H -1.7472910000 8.9230150000 -1.1879690000
H -1.5879930000 8.6123810000 -3.3899880000
H -0.8592920000 7.3591580000 -5.4029940000
H 0.5748060000 5.3428860000 -5.0896400000
H 0.7908010000 3.5791450000 -2.3846870000
H 1.9026420000 3.9290030000 -3.6913110000
H 3.9638940000 3.2209140000 -2.6731000000
H 5.7278790000 3.5635920000 -0.9499770000
H 5.2897920000 5.1505150000 0.9040920000
H 4.0643200000 6.8229130000 1.8849160000
H 2.7032610000 7.5462200000 1.0110690000
H 0.0255600000 7.4609090000 1.7126290000
S -0.1446860000 3.4892540000 0.5548740000
H 0.5228560000 2.6945090000 1.4163520000
C -1.7634160000 2.7249150000 0.8785740000
H -1.8887030000 1.8684310000 0.2121360000
H -2.5038070000 3.4711370000 0.6003280000
C -1.9835840000 2.3178360000 2.3238700000
H -1.7385500000 3.1537070000 2.9839780000
C -3.4737080000 2.0791970000 2.5540490000
O -4.3142070000 2.8803630000 2.2301280000

N -1.1211410000 1.2285820000 2.7030500000
H -1.0429310000 0.4519330000 2.0651250000
C -0.7967130000 1.0086800000 4.0167410000
O -1.0714170000 1.8070130000 4.8914100000
C -0.0805930000 -0.2833320000 4.2817780000
H 0.6631590000 -0.1348960000 5.0601950000
H -0.7999770000 -1.0138130000 4.6543110000
H 0.3980870000 -0.7008140000 3.3977140000
O -3.7197130000 0.9198410000 3.1590460000
C -5.1053310000 0.6683990000 3.4907430000
H -5.6833690000 0.6466030000 2.5651740000
H -5.4725720000 1.5070250000 4.0838680000
C -5.1645600000 -0.6291520000 4.2358640000
H -4.5769230000 -0.5844630000 5.1518250000
H -4.7914130000 -1.4536330000 3.6296470000
H -6.1947100000 -0.8525140000 4.5079170000

Ru-4 (NACET)(gas)

Ru -0.1134730000 5.8721300000 1.3524990000
P -1.9124610000 6.8691920000 0.2238880000
P 1.8763820000 5.9502620000 2.5494350000
O -1.7347070000 5.3175010000 3.7938940000
N 1.0965050000 6.1702820000 -0.6019440000
C -1.0970050000 5.5432890000 2.8444860000
C -2.3113740000 8.7787820000 2.2493370000
C -4.1336600000 7.0777300000 1.9546790000
C -3.0601880000 7.9021110000 1.2572090000
C -4.1024080000 6.8682660000 -1.5971950000
C -2.4731100000 4.9835540000 -1.8425110000
C -3.1189900000 5.9635290000 -0.8678640000
C 3.9898280000 4.5159950000 3.8270040000
C 1.7011840000 3.4734890000 3.8831100000
C 2.6607670000 4.3626900000 3.1049880000
C 0.9626490000 6.7967640000 5.0816250000
C 1.9110450000 8.5595720000 3.5895990000
C 1.9915520000 7.0972730000 4.0030380000
C 0.4831180000 6.4094680000 -1.8317620000
C 2.3120870000 5.4836510000 -0.6872360000
C -1.0748180000 8.1301520000 -0.8272250000
C -0.4622180000 7.4508430000 -2.0026810000
C -0.9234670000 7.7781310000 -3.2730910000
C -0.5216320000 7.0861330000 -4.4022930000
C 0.3144240000 5.9934880000 -4.2320910000
C 0.7837780000 5.6387920000 -2.9795030000

C 1.5467010000 4.3750150000 -2.7645830000
C 2.6111230000 4.5988900000 -1.7464830000
C 3.8420230000 3.9698650000 -1.8333520000
C 4.8216970000 4.1711240000 -0.8747220000
C 4.5517860000 5.0428740000 0.1656660000
C 3.3350480000 5.7081820000 0.2643630000
C 3.1070800000 6.6522470000 1.3886440000
H -1.8630670000 8.1837890000 3.0446100000
H -1.5135550000 9.3535650000 1.7790440000
H -3.0008000000 9.4857410000 2.7130320000
H -4.7457170000 7.7230830000 2.5861860000
H -4.8039440000 6.5817150000 1.2547790000
H -3.7022600000 6.3121930000 2.5982470000
H -3.5463690000 8.5650750000 0.5329730000
H -4.7991160000 6.2688260000 -2.1851480000
H -4.6955920000 7.4853300000 -0.9244400000
H -3.5811230000 7.5259120000 -2.2941330000
H -2.1477100000 5.4783140000 -2.7561010000
H -1.5958820000 4.4896300000 -1.4248310000
H -3.1929220000 4.2125450000 -2.1237170000
H -3.6719230000 5.3736800000 -0.1263680000
H 4.4760570000 3.5457800000 3.9385440000
H 4.6835330000 5.1659260000 3.2945080000
H 3.8523870000 4.9199810000 4.8309270000
H 1.5936520000 3.7899550000 4.9200840000
H 0.6990680000 3.4639190000 3.4537740000
H 2.0644190000 2.4438530000 3.9076570000
H 2.8554770000 3.8872560000 2.1350330000
H -0.0370640000 7.0736820000 4.7497120000
H 0.9293850000 5.7470040000 5.3667150000
H 1.1792240000 7.3774280000 5.9792280000
H 1.9666590000 9.1959600000 4.4736980000
H 2.7180590000 8.8551340000 2.9222950000
H 0.9654770000 8.7759330000 3.0901910000
H 2.9935440000 6.9161800000 4.4065740000
H -0.3322980000 8.6026330000 -0.1805520000
H -1.7841550000 8.8942210000 -1.1533020000
H -1.6229630000 8.6026200000 -3.3642260000
H -0.8755080000 7.3717990000 -5.3834800000
H 0.5951480000 5.3818340000 -5.0829650000
H 0.8529150000 3.5985180000 -2.4056750000
H 1.9634560000 3.9953840000 -3.6991480000
H 4.0260830000 3.3057630000 -2.6710170000
H 5.7796350000 3.6737790000 -0.9448640000

H 5.3116770000 5.2464450000 0.9129900000
H 4.0398270000 6.8945500000 1.9027610000
H 2.6552630000 7.5789040000 1.0270400000
H -0.0047350000 7.4177380000 1.7266520000
S -0.0649770000 3.4674910000 0.4895880000
H 0.6118690000 2.6587970000 1.3304240000
C -1.6708530000 2.6680800000 0.7794970000
H -1.7598060000 1.8003730000 0.1215160000
H -2.4189530000 3.3936380000 0.4696200000
C -1.9262130000 2.2775380000 2.2225220000
H -1.6397490000 3.0990740000 2.8846160000
C -3.4269490000 2.1268330000 2.4549030000
O -4.2332970000 2.9180110000 2.0382590000
N -1.1245490000 1.1472820000 2.6201950000
H -1.1376960000 0.3379700000 2.0205250000
C -0.7872160000 0.9534680000 3.9390170000
O -0.9693730000 1.8048630000 4.7825950000
C -0.1729780000 -0.3839620000 4.2444110000
H 0.6222290000 -0.2568250000 4.9735640000
H -0.9314010000 -1.0216130000 4.6987910000
H 0.2187470000 -0.8930740000 3.3658240000
O -3.7219550000 1.0558810000 3.1932710000
C -5.1126880000 0.9193060000 3.5563260000
H -5.6996310000 0.8093300000 2.6426870000
H -5.4336670000 1.8461230000 4.0335750000
C -5.2269470000 -0.2674990000 4.4653760000
H -4.6230570000 -0.1326370000 5.3610480000
H -4.9006060000 -1.1797630000 3.9685810000
H -6.2618280000 -0.4042230000 4.7727580000

Ru-4-NACET (NACET)(gas)

Ru -0.6987130000 4.5340550000 0.8897610000
P -2.6954400000 5.6342130000 0.3399920000
P 1.5057780000 4.4573820000 1.6086610000
O -1.6230760000 3.6280830000 3.5749580000
N 0.0493020000 5.1284490000 -1.2378470000
C -1.2759250000 3.9820860000 2.5211710000
C -2.6793760000 7.1720150000 2.6907160000
C -4.4719740000 5.4209610000 2.5108210000
C -3.6085520000 6.4197650000 1.7505520000
C -5.2256200000 5.8311530000 -0.9486120000
C -3.6541930000 4.1211510000 -1.8794670000
C -4.0903990000 4.8701420000 -0.6245530000
C 3.7902950000 2.8552640000 2.2379930000

C 1.5399030000 1.9596380000 2.8993240000
C 2.3097330000 2.8070590000 1.8950070000
C 1.0729870000 5.2702080000 4.2849130000
C 2.0147200000 6.9764970000 2.7383250000
C 1.9743640000 5.4930610000 3.0803330000
C -0.8085280000 5.5882770000 -2.2355040000
C 1.1455330000 4.3939590000 -1.6971160000
C -2.0849560000 7.0824840000 -0.6278220000
C -1.7337610000 6.6384940000 -2.0078190000
C -2.4402120000 7.1891440000 -3.0722810000
C -2.3127780000 6.7167250000 -4.3667810000
C -1.5160700000 5.6013560000 -4.5740980000
C -0.8108910000 5.0237610000 -3.5335220000
C -0.1383890000 3.7022000000 -3.6951460000
C 1.1239940000 3.6670840000 -2.9076070000
C 2.2222280000 2.9377220000 -3.3310880000
C 3.3862820000 2.8875250000 -2.5801360000
C 3.4415510000 3.6245480000 -1.4098140000
C 2.3625900000 4.3859870000 -0.9747020000
C 2.4915850000 5.2012200000 0.2598960000
H -2.0543740000 6.4857330000 3.2611210000
H -2.0173800000 7.8587680000 2.1630020000
H -3.2642510000 7.7552780000 3.4032330000
H -4.9622630000 5.9152090000 3.3504330000
H -5.2542300000 4.9827020000 1.8930600000
H -3.8811770000 4.6038110000 2.9252470000
H -4.2640540000 7.1560130000 1.2726350000
H -6.0154860000 5.3065400000 -1.4878700000
H -5.6787750000 6.2717500000 -0.0621930000
H -4.8789200000 6.6395760000 -1.5944600000
H -3.5752510000 4.7840480000 -2.7399570000
H -2.6812620000 3.6425210000 -1.7627620000
H -4.3892160000 3.3503920000 -2.1196510000
H -4.4589710000 4.1195660000 0.0849910000
H 4.2181810000 1.8519130000 2.2069400000
H 4.3626790000 3.4725610000 1.5470660000
H 3.9552640000 3.2384150000 3.2458080000
H 1.6611690000 2.3357230000 3.9160030000
H 0.4708520000 1.9322150000 2.6831250000
H 1.9008730000 0.9305960000 2.8880100000
H 2.2064000000 2.3423220000 0.9049250000
H 0.0810610000 5.6816110000 4.0995600000
H 0.9441470000 4.2222340000 4.5461220000
H 1.4809150000 5.7836540000 5.1566360000

H 2.2224470000 7.5557910000 3.6387450000
H 2.7800140000 7.2233190000 2.0058570000
H 1.0523020000 7.3149030000 2.3487010000
H 2.9914150000 5.1740160000 3.3315860000
H -1.2259690000 7.4634280000 -0.0720010000
H -2.8433920000 7.8679510000 -0.6655950000
H -3.1165200000 8.0110570000 -2.8617630000
H -2.8535530000 7.1779230000 -5.1818530000
H -1.4620040000 5.1418080000 -5.5554410000
H -0.8186630000 2.9161350000 -3.3246500000
H 0.0397260000 3.4678740000 -4.7464410000
H 2.1539350000 2.3971390000 -4.2693080000
H 4.2405390000 2.3136080000 -2.9136760000
H 4.3568980000 3.6481310000 -0.8275640000
H 3.5362830000 5.3262620000 0.5524240000
H 2.0500970000 6.1885590000 0.1068580000
H -0.4713800000 6.0101750000 1.4601240000
S -0.7937450000 2.2899680000 -0.2876920000
H -0.0467810000 1.4551080000 0.4584430000
C -2.3363820000 1.3386500000 -0.0993600000
H -2.1229000000 0.2995150000 -0.3603440000
H -3.0144200000 1.7259520000 -0.8563360000
C -2.9989040000 1.4327390000 1.2619910000
H -3.1772260000 2.4900650000 1.4941850000
C -4.3885580000 0.8188650000 1.1593190000
O -5.3303040000 1.4244280000 0.7194490000
N -2.1689360000 0.8363920000 2.2710180000
H -1.2834650000 0.4266970000 2.0033440000
C -2.5921790000 0.7739280000 3.5629960000
O -3.6854020000 1.1888770000 3.8996790000
C -1.6021440000 0.1727900000 4.5221760000
H -1.1982480000 0.9679040000 5.1483140000
H -2.1099760000 -0.5350180000 5.1733340000
H -0.7714860000 -0.3217240000 4.0171600000
O -4.4018480000 -0.4635600000 1.5449700000
C -5.6858970000 -1.1192600000 1.5884940000
H -5.8021290000 -1.6777850000 0.6550970000
H -6.4619710000 -0.3550730000 1.6082210000
C -5.7105070000 -2.0091420000 2.7977600000
H -5.6126980000 -1.4192550000 3.7073740000
H -4.8969790000 -2.7335840000 2.7816230000
H -6.6528050000 -2.5529590000 2.8424010000
S -3.4804520000 -2.2151840000 -1.3096000000
H -2.3673870000 -2.2532360000 -2.0546600000

C -2.8315640000 -3.1364690000 0.1056870000
H -2.5272670000 -4.1381630000 -0.2037930000
H -3.6728600000 -3.2612670000 0.7844290000
C -1.7199160000 -2.4312810000 0.8614260000
H -2.0732170000 -1.4261450000 1.1160010000
H -0.3035300000 -3.0130590000 -0.6006150000
N -0.5318500000 -2.2807360000 0.0506640000
C 0.3891990000 -1.3152700000 0.2859700000
C 1.5837430000 -1.2837300000 -0.6161300000
H 1.6024920000 -2.0845520000 -1.3515330000
H 1.6050870000 -0.3261430000 -1.1374220000
H 2.4867470000 -1.3339340000 -0.0106110000
C -1.4861140000 -3.0891060000 2.2184830000
O -2.3872790000 -3.4561420000 2.9267720000
O -0.1926370000 -3.1398730000 2.5333800000
C 0.1256910000 -3.5721360000 3.8745210000
H -0.6193100000 -3.1607400000 4.5560550000
H 0.0347140000 -4.6592520000 3.9143060000
C 1.5159550000 -3.0958390000 4.1753290000
H 2.2400870000 -3.5213080000 3.4825390000
H 1.8029250000 -3.3870690000 5.1837280000
H 1.5762990000 -2.0104380000 4.1038950000
O 0.2396580000 -0.4891980000 1.1796250000

Ru-6

Ru 0.5945290000 -0.2289610000 -1.4621400000
P 2.2027750000 -0.0362680000 0.1503910000
P -1.3485830000 -1.0776280000 -0.3170400000
N -0.1795550000 1.7884980000 -0.8216150000
C 1.9826840000 1.6274250000 0.8990760000
H 1.0422240000 1.5612350000 1.4555490000
H 2.7836530000 1.8077070000 1.6159680000
C 1.8948500000 2.7369630000 -0.0949800000
C 2.8747200000 3.7166470000 -0.1968500000
H 3.7632370000 3.6417240000 0.4214880000
C 2.7090280000 4.8036200000 -1.0427150000
H 3.4749110000 5.5651070000 -1.1089070000
C 1.5378170000 4.9191670000 -1.7813710000
H 1.3869720000 5.7789910000 -2.4266070000
C 0.5576970000 3.9407620000 -1.7234220000
C 0.7446040000 2.8162170000 -0.9035780000
C -0.6941610000 3.9864680000 -2.5474780000
H -0.5105200000 3.4944910000 -3.5150270000
H -0.9695090000 5.0141550000 -2.7953940000

C -1.8202320000 3.2723450000 -1.8627590000
C -3.1444540000 3.6501620000 -2.0167950000
H -3.3743380000 4.5232030000 -2.6190350000
C -4.1721850000 2.9168940000 -1.4362870000
H -5.2040900000 3.2133990000 -1.5722130000
C -3.8555250000 1.8104000000 -0.6616730000
H -4.6443840000 1.2522840000 -0.1684230000
C -2.5337360000 1.4347820000 -0.4474880000
C -1.4906110000 2.1503510000 -1.0735470000
C -2.1887200000 0.3373500000 0.5021110000
H -3.0771690000 -0.0090870000 1.0318670000
H -1.4770240000 0.7033600000 1.2491020000
C 2.1422160000 -1.2087180000 1.5868240000
H 1.0769220000 -1.4383900000 1.6199280000
C 2.8802240000 -2.5123220000 1.3186930000
H 3.9596380000 -2.3868430000 1.3920810000
H 2.6628130000 -2.9291220000 0.3355040000
H 2.5959650000 -3.2592470000 2.0621780000
C 2.5240710000 -0.6246560000 2.9394590000
H 2.3790340000 -1.3814460000 3.7130430000
H 1.9125140000 0.2339560000 3.2119820000
H 3.5685070000 -0.3191100000 2.9832660000
C 3.9649890000 0.0410470000 -0.4307550000
H 3.9353950000 0.9416550000 -1.0535700000
C 4.4059590000 -1.1070240000 -1.3245650000
H 5.3787560000 -0.8705380000 -1.7590500000
H 3.7169950000 -1.2645810000 -2.1503980000
H 4.5183320000 -2.0427470000 -0.7791920000
C 4.9796510000 0.2797380000 0.6808810000
H 5.1252490000 -0.6011150000 1.3058870000
H 4.7236220000 1.1141550000 1.3317470000
H 5.9480800000 0.5113910000 0.2343370000
C -2.6323270000 -1.5750880000 -1.5711170000
H -2.7269260000 -0.6504920000 -2.1430470000
C -4.0037600000 -1.9051910000 -1.0017990000
H -4.7354030000 -1.9268800000 -1.8121960000
H -4.3570060000 -1.1738270000 -0.2763150000
H -4.0334680000 -2.8883340000 -0.5303720000
C -2.1644140000 -2.6675040000 -2.5179130000
H -2.0613920000 -3.6286920000 -2.0117330000
H -1.2113200000 -2.4313400000 -2.9876550000
H -2.8961100000 -2.8112430000 -3.3156030000
C -1.4744880000 -2.4533970000 0.9408120000
H -2.4882540000 -2.8337570000 0.7900920000

C -0.5107850000 -3.5877770000 0.6321260000
H 0.5279960000 -3.2828290000 0.7360580000
H -0.6288950000 -3.9670560000 -0.3817260000
H -0.6767660000 -4.4226590000 1.3152520000
C -1.4011170000 -1.9979290000 2.3923420000
H -1.4469620000 -2.8668950000 3.0508580000
H -2.2317710000 -1.3475540000 2.6603870000
H -0.4839480000 -1.4632770000 2.6379730000
C 1.1762040000 -1.8938230000 -1.9303000000
O 1.5297970000 -2.9587520000 -2.2373720000
S 2.1511650000 0.8811370000 -3.0087410000
C 1.6592510000 0.0877900000 -4.5672730000
H 2.4952640000 0.1616420000 -5.2636690000
C 0.4583550000 0.7670560000 -5.1930070000
H 0.2367110000 0.3671100000 -6.1878190000
C -0.7971930000 0.5786020000 -4.3586570000
O -0.8662390000 0.0759870000 -3.2472800000
H 1.4559460000 -0.9733420000 -4.4165980000
O -1.8625100000 1.0162960000 -4.9974360000
C -3.1416760000 0.9228590000 -4.3188500000
H -3.0121710000 1.2437780000 -3.2839440000
H -3.4362990000 -0.1299160000 -4.3140980000
C -4.1110830000 1.7825530000 -5.0678340000
H -4.2203840000 1.4570900000 -6.1011420000
H -3.7874480000 2.8226870000 -5.0693400000
H -5.0875640000 1.7350540000 -4.5886680000
N 0.7139910000 2.1771950000 -5.3713960000
H 1.2046490000 2.6149240000 -4.6008500000
C 0.4172130000 2.8718030000 -6.5006530000
O -0.1013910000 2.3603460000 -7.4786980000
C 0.7557000000 4.3353540000 -6.4332910000
H -0.1719650000 4.9064030000 -6.3902200000
H 1.3615790000 4.6017630000 -5.5686070000
H 1.2742420000 4.6333780000 -7.3417090000

Ru-6 (gas)

Ru 0.5945250000 -0.2409970000 -1.4627660000
P 2.1991700000 -0.0297700000 0.1441380000
P -1.3462440000 -1.0802480000 -0.3137980000
N -0.1804540000 1.7745380000 -0.8337330000
C 1.9752910000 1.6372270000 0.8850920000
H 1.0285390000 1.5747410000 1.4315490000
H 2.7711930000 1.8246350000 1.6059900000

C 1.8937580000 2.7333480000 -0.1241370000
C 2.8760400000 3.7085230000 -0.2402900000
H 3.7644300000 3.6414810000 0.3791500000
C 2.7105300000 4.7830150000 -1.1006910000
H 3.4766150000 5.5425590000 -1.1782060000
C 1.5384880000 4.8900560000 -1.8384350000
H 1.3866050000 5.7432430000 -2.4917850000
C 0.5572730000 3.9142730000 -1.7665890000
C 0.7441180000 2.8022670000 -0.9320220000
C -0.7007600000 3.9555660000 -2.5814320000
H -0.5284440000 3.4554370000 -3.5470610000
H -0.9756440000 4.9822620000 -2.8348240000
C -1.8232290000 3.2507550000 -1.8814810000
C -3.1467640000 3.6322520000 -2.0271870000
H -3.3776840000 4.5034040000 -2.6315770000
C -4.1732370000 2.9059500000 -1.4365360000
H -5.2045290000 3.2064970000 -1.5648450000
C -3.8549930000 1.8038670000 -0.6572570000
H -4.6427340000 1.2543940000 -0.1526640000
C -2.5330880000 1.4260220000 -0.4495040000
C -1.4915760000 2.1344910000 -1.0852530000
C -2.1830020000 0.3364720000 0.5079210000
H -3.0688730000 -0.0067230000 1.0444570000
H -1.4642060000 0.7083930000 1.2454500000
C 2.1383860000 -1.1976800000 1.5862790000
H 1.0729950000 -1.4292840000 1.6159730000
C 2.8782830000 -2.5025910000 1.3255710000
H 3.9572540000 -2.3731740000 1.3955020000
H 2.6595710000 -2.9236190000 0.3445490000
H 2.5968550000 -3.2454670000 2.0739050000
C 2.5150100000 -0.6066960000 2.9381920000
H 2.3741620000 -1.3599480000 3.7155320000
H 1.8986460000 0.2504040000 3.2035220000
H 3.5570240000 -0.2938070000 2.9798450000
C 3.9629720000 0.0469410000 -0.4316810000
H 3.9331350000 0.9460630000 -1.0570660000
C 4.4067260000 -1.1031370000 -1.3228530000
H 5.3863190000 -0.8730060000 -1.7438200000
H 3.7250870000 -1.2507140000 -2.1559690000
H 4.5036340000 -2.0408400000 -0.7784570000
C 4.9732800000 0.2891420000 0.6839220000
H 5.1072990000 -0.5869240000 1.3181460000
H 4.7173890000 1.1314000000 1.3244960000
H 5.9461980000 0.5087600000 0.2423580000

C -2.6360100000 -1.5792130000 -1.5611890000
H -2.7185960000 -0.6615070000 -2.1455760000
C -4.0122940000 -1.8857530000 -0.9895510000
H -4.7402910000 -1.9289920000 -1.8016800000
H -4.3620770000 -1.1297900000 -0.2887590000
H -4.0487990000 -2.8536400000 -0.4882320000
C -2.1750590000 -2.6900120000 -2.4910110000
H -2.1022480000 -3.6476020000 -1.9730630000
H -1.2084360000 -2.4769430000 -2.9430750000
H -2.8953240000 -2.8255460000 -3.2999240000
C -1.4718810000 -2.4632040000 0.9389110000
H -2.4834910000 -2.8480070000 0.7847420000
C -0.5012130000 -3.5916900000 0.6260500000
H 0.5345330000 -3.2785760000 0.7327330000
H -0.6123050000 -3.9618910000 -0.3915960000
H -0.6627630000 -4.4319100000 1.3030530000
C -1.4031470000 -2.0150740000 2.3935840000
H -1.4572200000 -2.8856340000 3.0487140000
H -2.2294470000 -1.3585350000 2.6586400000
H -0.4834020000 -1.4873830000 2.6434760000
C 1.1868550000 -1.9094930000 -1.9161170000
O 1.5483430000 -2.9766830000 -2.2017480000
S 2.1496280000 0.8673210000 -2.9988360000
C 1.6703920000 0.0686130000 -4.5578770000
H 2.5107870000 0.1433400000 -5.2481800000
C 0.4732780000 0.7529420000 -5.1857010000
H 0.2473030000 0.3586090000 -6.1827560000
C -0.7844050000 0.5658600000 -4.3535720000
O -0.8615510000 0.0412310000 -3.2522940000
H 1.4649400000 -0.9915470000 -4.4049800000
O -1.8423800000 1.0334310000 -4.9829910000
C -3.1223860000 0.9571260000 -4.3081920000
H -2.9862690000 1.2356070000 -3.2619890000
H -3.4514560000 -0.0851760000 -4.3426040000
C -4.0592650000 1.8801830000 -5.0251420000
H -4.1761980000 1.6009620000 -6.0704060000
H -3.6916110000 2.9043470000 -4.9827250000
H -5.0359760000 1.8554320000 -4.5456820000
N 0.7352420000 2.1612730000 -5.3646140000
H 1.2310540000 2.5970810000 -4.5961920000
C 0.3850220000 2.8575000000 -6.4817020000
O -0.1637730000 2.3419230000 -7.4368040000
C 0.7121460000 4.3263190000 -6.4248050000
H -0.2194200000 4.8864780000 -6.3498220000

H 1.3444360000 4.5984310000 -5.5813290000
H 1.1942690000 4.6271290000 -7.3513490000

Ru-6-NACET(gas)

Ru 0.1284450000 -0.8201360000 -2.1942900000
P 1.7218830000 -0.8153600000 -0.5643950000
P -1.8537870000 -1.6810410000 -1.1418260000
N -0.5737740000 1.1634320000 -1.4046300000
C 1.5495140000 0.7859080000 0.3202490000
H 0.5932970000 0.7134460000 0.8487990000
H 2.3416500000 0.8773350000 1.0635050000
C 1.5260020000 1.9685490000 -0.5893710000
C 2.5485300000 2.9081230000 -0.6077530000
H 3.4246550000 2.7492050000 0.0123560000
C 2.4383090000 4.0622300000 -1.3682000000
H 3.2348350000 4.7938710000 -1.3678420000
C 1.2811180000 4.2844160000 -2.1036490000
H 1.1713150000 5.2004730000 -2.6753310000
C 0.2608570000 3.3470320000 -2.1315880000
C 0.3915070000 2.1567730000 -1.3999940000
C -0.9836560000 3.5136980000 -2.9517090000
H -0.8187670000 3.1020000000 -3.9596840000
H -1.2159190000 4.5698810000 -3.1088750000
C -2.1428650000 2.7906490000 -2.3341890000
C -3.4486080000 3.2357120000 -2.4594770000
H -3.6373410000 4.1663090000 -2.9848290000
C -4.5102660000 2.5018030000 -1.9457210000
H -5.5272640000 2.8532560000 -2.0573700000
C -4.2457630000 1.3230860000 -1.2642210000
H -5.0610100000 0.7627690000 -0.8188310000
C -2.9426520000 0.8756010000 -1.0779880000
C -1.8662570000 1.5959060000 -1.6375320000
C -2.6465410000 -0.3046260000 -0.2147070000
H -3.5511480000 -0.6569240000 0.2832490000
H -1.9225480000 -0.0243920000 0.5574480000
C 1.6122830000 -2.1041360000 0.7675080000
H 0.5398480000 -2.3016130000 0.7738120000
C 2.3092280000 -3.4053960000 0.3947280000
H 3.3917380000 -3.3191520000 0.4753130000
H 2.0781170000 -3.7329230000 -0.6185910000
H 2.0010750000 -4.1999930000 1.0763240000
C 1.9999840000 -1.6463530000 2.1672770000
H 1.8341720000 -2.4612560000 2.8742050000
H 1.4069690000 -0.7988590000 2.5064860000

H 3.0502320000 -1.3682560000 2.2386960000
C 3.4913550000 -0.7461350000 -1.1238390000
H 3.4945530000 0.2056770000 -1.6665580000
C 3.9046770000 -1.8270240000 -2.1109210000
H 4.8938340000 -1.5922840000 -2.5061280000
H 3.2244170000 -1.8798770000 -2.9565570000
H 3.9683580000 -2.8112890000 -1.6504920000
C 4.5008680000 -0.6349470000 0.0129550000
H 4.6038670000 -1.5672980000 0.5676020000
H 4.2661720000 0.1551100000 0.7242540000
H 5.4830090000 -0.4073610000 -0.4034550000
C -3.1414590000 -2.0319890000 -2.4396260000
H -3.1894540000 -1.0673550000 -2.9472760000
C -4.5329920000 -2.3393620000 -1.9062920000
H -5.2526260000 -2.2868890000 -2.7250210000
H -4.8648400000 -1.6360710000 -1.1443250000
H -4.6072370000 -3.3457530000 -1.4928780000
C -2.7050430000 -3.0754650000 -3.4550630000
H -2.6541540000 -4.0728570000 -3.0154290000
H -1.7355600000 -2.8461200000 -3.8933610000
H -3.4244080000 -3.1267090000 -4.2735760000
C -2.0430930000 -3.1502810000 -0.0017280000
H -3.0682980000 -3.4791210000 -0.1915570000
C -1.1185400000 -4.2924360000 -0.3948440000
H -0.0721850000 -4.0436020000 -0.2357640000
H -1.2202470000 -4.5639420000 -1.4440040000
H -1.3381480000 -5.1806020000 0.1994450000
C -1.9694620000 -2.8171340000 1.4833550000
H -2.0532790000 -3.7329930000 2.0699590000
H -2.7774870000 -2.1580220000 1.7943630000
H -1.0362700000 -2.3372280000 1.7763320000
C 0.6625690000 -2.4651650000 -2.7879920000
O 0.9903280000 -3.5146940000 -3.1628370000
S 1.7411210000 0.3570560000 -3.6256410000
C 1.2326930000 -0.2919560000 -5.2426300000
H 2.0782380000 -0.2155910000 -5.9271900000
C 0.0786730000 0.5072150000 -5.8095220000
H -0.1746540000 0.1814430000 -6.8243270000
C -1.1885510000 0.3289570000 -4.9869530000
O -1.3016350000 -0.3134660000 -3.9567330000
H 0.9571190000 -1.3457240000 -5.1754360000
O -2.2035570000 0.9594650000 -5.5438340000
C -3.5141470000 0.7715290000 -4.9508760000
H -3.4358440000 0.9204420000 -3.8730360000

H -3.7988230000 -0.2691520000 -5.1356570000
C -4.4437000000 1.7516930000 -5.5977400000
H -4.5071890000 1.5988060000 -6.6740400000
H -4.1142570000 2.7728830000 -5.4124550000
H -5.4409850000 1.6416800000 -5.1763480000
N 0.4286310000 1.9078380000 -5.8924810000
H 0.9136950000 2.2576570000 -5.0727220000
C 0.1431000000 2.7159550000 -6.9365550000
O -0.3956890000 2.3236080000 -7.9661630000
C 0.5157120000 4.1577620000 -6.7419950000
H -0.4002780000 4.7395840000 -6.6435190000
H 1.1287650000 4.3303220000 -5.8598100000
H 1.0349050000 4.5225830000 -7.6244460000
C -1.8713470000 -0.6140070000 -9.5864250000
N -0.5086390000 -0.2561080000 -9.3028990000
H -0.3521010000 0.6653940000 -8.9042840000
H -1.8304000000 -1.5766900000 -10.1082930000
H -3.7341320000 -0.9107870000 -8.5567030000
C -2.6889940000 -0.7380640000 -8.3084320000
H -2.6293930000 0.2009420000 -7.7554130000
S -2.1793910000 -2.1134470000 -7.2426190000
H -0.8602860000 -1.9834800000 -7.4557070000
H 1.8380180000 0.5303350000 -8.8696130000
C 1.8963240000 -0.4107250000 -9.4149850000
H 2.4550980000 -1.1335090000 -8.8229220000
H 2.4580870000 -0.2515650000 -10.3330230000
C 0.5479020000 -0.9893500000 -9.7553710000
O 0.4201520000 -2.0386260000 -10.3579590000
O -3.7600140000 0.5087150000 -10.5779870000
C -2.5644430000 0.3712020000 -10.5196010000
O -1.6895040000 1.0426160000 -11.2714970000
C -2.2643710000 1.9921440000 -12.1900090000
H -2.9273960000 2.6549950000 -11.6317170000
H -2.8844590000 1.4500890000 -12.9066690000
C -1.1332690000 2.7267020000 -12.8457650000
H -0.5300770000 3.2523260000 -12.1074580000
H -0.4834440000 2.0444280000 -13.3907410000
H -1.5217540000 3.4594890000 -13.5504990000

EtSH

C -9.9554910000 -0.0975310000 0.0300910000
C -8.4485490000 -0.0703670000 -0.0257910000
H -10.3938670000 0.3513080000 -0.8613150000
H -10.3330390000 0.4479390000 0.8937850000

H -10.3182120000 -1.1247050000 0.0933020000
S -7.7328230000 1.6024450000 -0.0821120000
H -8.0698120000 -0.6430580000 -0.8719550000
H -8.0141170000 -0.5158590000 0.8692850000
H -8.3251710000 1.9807280000 -1.2235780000

NACET

S 2.0671240000 2.3839040000 0.7947710000
C 2.3138630000 0.9667990000 1.8922630000
C 3.7311660000 0.4167680000 1.8705570000
C 3.9286560000 -0.5640580000 3.0210050000
N 4.0840340000 -0.1177620000 0.5809610000
O 4.7523840000 -1.5607550000 2.6944930000
O 3.4222830000 -0.4237070000 4.1056410000
C 5.0653490000 -2.4868130000 3.7600160000
C 6.0121110000 -3.5086550000 3.2100230000
C 5.3084270000 0.1029280000 0.0140050000
O 6.1185720000 0.8747050000 0.4922080000
C 5.5743980000 -0.6804840000 -1.2386970000
H 2.5469610000 1.7894650000 -0.3080310000
H 1.5947770000 0.1797250000 1.6568460000
H 2.0794250000 1.3180630000 2.8946670000
H 4.4422250000 1.2290970000 2.0535350000
H 3.5214800000 -0.8683550000 0.2136500000
H 4.1345800000 -2.9297960000 4.1187090000
H 5.4963250000 -1.9236660000 4.5893940000
H 5.5647040000 -4.0611750000 2.3848000000
H 6.2773900000 -4.2230910000 3.9874460000
H 6.9298870000 -3.0445520000 2.8518950000
H 4.6809930000 -1.1315880000 -1.6664270000
H 6.2835050000 -1.4763550000 -1.0102940000
H 6.0415020000 -0.0362660000 -1.9794330000

NACET (gas)

S 2.0210200000 2.4085260000 0.8393730000
C 2.2833780000 0.9688290000 1.9002020000
C 3.7048840000 0.4317670000 1.8589900000
C 3.9297780000 -0.5460250000 3.0066990000
N 4.0513000000 -0.0974330000 0.5636740000
O 4.7400480000 -1.5525600000 2.6577100000
O 3.4563160000 -0.4035720000 4.1035920000
C 5.0771070000 -2.4677950000 3.7211660000
C 6.0210920000 -3.4880350000 3.1584360000

C 5.3058930000 0.0877820000 0.0358860000
O 6.1060400000 0.8580540000 0.5225610000
C 5.6048640000 -0.7387450000 -1.1839600000
H 2.5319130000 1.8526570000 -0.2685460000
H 1.5696840000 0.1799360000 1.6529750000
H 2.0565880000 1.2900260000 2.9142780000
H 4.4098980000 1.2521420000 2.0298880000
H 3.5170860000 -0.8814780000 0.2261250000
H 4.1556610000 -2.9117490000 4.1020620000
H 5.5170530000 -1.8965330000 4.5400280000
H 5.5609240000 -4.0465260000 2.3448970000
H 6.3088940000 -4.1973040000 3.9319480000
H 6.9259200000 -3.0174020000 2.7780290000
H 4.7127510000 -1.1064660000 -1.6876170000
H 6.2043550000 -1.5983440000 -0.8838310000
H 6.1996710000 -0.1553790000 -1.8805610000

Hydrogen

H -5.8397280000 -0.7819140000 0.0000000000
H -5.0989870000 -0.8555830000 0.0000000000

Supplemental References

1. Zou, Y.-Q.; von Wolff, N.; Anaby, A.; Xie, Y.; Milstein, D. Ethylene Glycol as an Efficient and Reversible Liquid Organic Hydrogen Carrier. **2019**, *Nat. Catal. 2*, 415-422.
2. Luo, J.; Rauch, M.; Avram, L.; Ben-David, Y.; Milstein, D. Formation of Thioesters by Dehydrogenative Coupling of Thiols and Alcohols with H_2 Evolution. *Nat. Catal.* **2020**, *3*, 887-892.
3. Akdemir, N., Gümrükcüoğlu, İ E. and Ağar, E. Synthesis and Characterization of Novel Phthalocyanines Containing N-(n-octyl)mercapto Acetamid Substituents. *Synth. React. Inorg. Met. Org. Chem.* **2005**, *35*, 819-824.
4. Tsikas, D.; Dehnert, S.; Urban, K.; Surdacki, A.; Meyer, H. H. (2009). GC-MS Analysis of S-Nitrosothiols after Conversion to S-Nitroso-N-acetylcysteineEthyl Ester and In-Injector Nitrosation of Ethyl Acetate. *J. Chromatogr. B* **2009**, *877*, 3442-3455.
5. Rühling, A.; Galla, H.-J.; Glorius, F. A Remarkably Simple Hybrid Surfactant–NHC Ligand, Its Gold-Complex, and Application in Micellar Catalysis. *Chem. Eur. J.* **2015**, *21*, 12291-12294.
6. Tang, S.; Rauch, M.; Montag, M.; Diskin-Posner, Y.; Ben-David, Y.; Milstein, D. Catalytic Oxidative Deamination by Water with H_2 Liberation. *J. Am. Chem. Soc.* **2020**, *142*, 20875-20882.
7. Sheldrick, G. Crystal Structure Refinement with SHELXL. *Acta Crystallogr. A* **2015**, *71*, 3-8.
8. Sheldrick, G. Crystal Structure Refinement with SHELXL. *Acta Crystallogr. C* **2015**, *71*, 3-8.
9. Dolomanov, O. V.; Bourhis, L. J.; Gildea, R. J.; Howard, J. A. K.; Puschmann, H. OLEX2: A Complete Structure Solution, Refinement and Analysis Program. *J. Appl. Cryst.* **2009**, *42*, 339-341.
10. Frisch, M. J. et al. *Gaussian 16, Revision C.01*; (Gaussian, Inc., Wallingford CT, 2016).

11. Zhao, Y.; Truhlar, D. G. A New Local Density Functional for Main-Group Thermochemistry, Transition Metal Bonding, Thermochemical Kinetics and Noncovalent Interactions. *J. Chem. Phys.* **2006**, *125*, 194101/1-18.
12. Weigend, F.; Ahlrichs, R. Balanced Basis Sets of Split Valence, Triple Zeta Valence and Quadruple Zeta Valence Quality for H to Rn: Design and Assessment of Accuracy. *Phys. Chem. Chem. Phys.* **2005**, *7*, 3297-3305.
13. Weigend, F. Accurate Coulomb-Fitting Basis Sets for H to Rn. *Phys. Chem. Chem. Phys.* **2006**, *8*, 1057-1065.
14. Grimme, S.; Antony, J.; Ehrlich, S.; Krieg, H. A Consistent and Accurate Ab Initio Parametrization of Density Functional Dispersion Correction (DFT-D) for the 94 Elements HPu. *J. Chem. Phys.* **2010**, *132*, 154104/1-19.
15. Neese, F. Software Update: The ORCA Program System, Version 4.0. *WIREs Comput. Mol. Sci.* **2018**, *8*, e1327-e1332.
16. Mardirossian, N.; Head-Gordon, M. ωB97X-V: A 10-Parameter, Range-Separated Hybrid, Generalized Gradient Approximation Density Functional with Nonlocal Correlation, Designed by a Survival-Of-The-Fittest Strategy. *Phys. Chem. Chem.* Phys. **2014**, *16*, 9904-9924.
17. Vydrova, O. A., and Voorhis, T. V. (2010). Nonlocal van der Waals density functional: The simpler the better. J. Chem. Phys. **2017**, *133*, 244103/1-9.
18. Hujo, W., and Grimme, S. (2011). Performance of the van der Waals Density Functional VV10 and (hybrid)GGA Variants for Thermochemistry and Noncovalent Interactions. J. Chem. Theory Comput. **2017**, *7*, 3866-3871.
19. Hellweg, A.; Hattig, C.; Hoefener, S.; Klopper, W. Optimized Accurate Auxiliary Basis Sets for RI-MP2 and RI-CC2 Calculations for the Atoms Rb to Rn. *Theor. Chem. Acc.* **2007**, *117*, 587-597.
20. Iron, M. A.; Janes, T. Evaluating Transition Metal Barrier Heights with the Latest Density Functional Theory Exchange-Correlation Functionals: The MOBH35 Benchmark Database. *J. Phys. Chem. A* **2019**, *123*, 3761-3781.
21. Marenich, A. V.; Cramer, C. J.; Truhlar, D. G. Universal Solvation Model Based on Solute Electron Density and on a Continuum Model of the Solvent Defined by

the Bulk Dielectric Constant and Atomic Surface Tensions. *J. Phys. Chem. B* **2009**, *113*, 6378-6396.

22. Cramer, C. J. Essentials of Computational Chemistry: *Theories and Models in 2nd Edition.* (John Wiley, and Sons Ltd: West Sussex, England, 2014).
23. Sparta, M.; Riplinger, C.; Neese, F. Mechanism of Olefin Asymmetric Hydrogenation Catalyzed by Iridium Phosphino-Oxazoline: A Pair Natural Orbital Coupled Cluster Study. *J. Chem. Theory and Computation* **2014**, *10,* 1099-1108.
24. Hopmann, K. H. How Accurate is DFT for Iridium-Mediated Chemistry?. *Organometallics* **2016**, *35*, 3795-3807.
25. Gusev, D. G. Revised Mechanisms of the Catalytic Alcohol Dehydrogenation and Ester Reduction with the Milstein PNN Complex of Ruthenium. *Organometallics* **2020**, *39*, 258-270.
